# Chinese herbal medicine for the treatment of chronic fatigue syndrome: A systematic review and meta-analysis

**DOI:** 10.3389/fphar.2022.958005

**Published:** 2022-09-29

**Authors:** Yang Zhang, Fangfang Jin, Xing Wei, Qiuyu Jin, Jingri Xie, Yujia Pan, Wenjuan Shen

**Affiliations:** ^1^ Department of Internal Medicine, First Affiliated Hospital of Heilongjiang University of Chinese Medicine, Harbin, China; ^2^ Department of Internal Medicine, Heilongjiang University of Chinese Medicine, Harbin, China; ^3^ Department of Obstetrics and Gynecology, Heilongjiang University of Chinese Medicine, Harbin, China; ^4^ Department of Obstetrics and Gynecology, First Affiliated Hospital of Heilongjiang University of Chinese Medicine, Harbin, China

**Keywords:** herbal medicine, chronic fatigue syndrome, treatment, systematic review, meta-analysis

## Abstract

**Objectives:** This meta-analysis aimed to assess the effectiveness and safety of Chinese herbal medicine (CHM) in treating chronic fatigue syndrome (CFS).

**Methods:** Nine electronic databases were searched from inception to May 2022. Two reviewers screened studies, extracted the data, and assessed the risk of bias independently. The meta-analysis was performed using the Stata 12.0 software.

**Results:** Eighty-four RCTs that explored the efficacy of 69 kinds of Chinese herbal formulas with various dosage forms (decoction, granule, oral liquid, pill, ointment, capsule, and herbal porridge), involving 6,944 participants were identified. This meta-analysis showed that the application of CHM for CFS can decrease Fatigue Scale scores (WMD: –1.77; 95%CI: –1.96 to –1.57; *p* < 0.001), Fatigue Assessment Instrument scores (WMD: –15.75; 95%CI: –26.89 to –4.61; *p <* 0.01), Self-Rating Scale of mental state scores (WMD: –9.72; 95%CI:–12.26 to –7.18; *p <* 0.001), Self-Rating Anxiety Scale scores (WMD: –7.07; 95%CI: –9.96 to –4.19; *p* < 0.001), Self-Rating Depression Scale scores (WMD: –5.45; 95%CI: –6.82 to –4.08; *p* < 0.001), and clinical symptom scores (WMD: –5.37; 95%CI: –6.13 to –4.60; *p* < 0.001) and improve IGA (WMD: 0.30; 95%CI: 0.20–0.41; *p <* 0.001), IGG (WMD: 1.74; 95%CI: 0.87–2.62; *p <* 0.001), IGM (WMD: 0.21; 95%CI: 0.14–0.29; *p <* 0.001), and the effective rate (RR = 1.41; 95%CI: 1.33–1.49; *p <* 0.001). However, natural killer cell levels did not change significantly. The included studies did not report any serious adverse events. In addition, the methodology quality of the included RCTs was generally not high.

**Conclusion:** Our study showed that CHM seems to be effective and safe in the treatment of CFS. However, given the poor quality of reports from these studies, the results should be interpreted cautiously. More international multi-centered, double-blinded, well-designed, randomized controlled trials are needed in future research.

**Systematic Review Registration**: [https://www.crd.york.ac.uk/prospero/display_record.php?ID=CRD42022319680], identifier [CRD42022319680].

## Introduction

Chronic fatigue syndrome (CFS) is a medically unexplained and debilitating mental and physical condition characterized by persistent fatigue (lasting for at least 6 months) and several other symptoms, including sleep disorders, lengthy malaise after exertion, sore throat, muscle pain, multi-joint pain, tender lymph nodes, headache, impairment of concentration or short-term memory, anxiety, and depression, which lead to severe disability and suffering in patients. Studies have shown that the prevalence of CFS is 0.006%–3% in the general population (Cleare et al., 2015), and 836,000–2.5 million people suffer from CFS in the US alone (Clayton, 2015). In addition, a meta-analysis showed that the overall incidence of CFS is 0.77% and 0.76% in Korea and Japan, respectively (Lim and Son, 2021). If there is no effective treatment, CFS will cause a decline in multi-system function and cause systemic diseases such as immune system, circulatory system, nervous system, digestive system, and visceral dysfunction, thus posing a serious threat to human health.

Although the cause of CFS remains uncertain, popular hypotheses include triggers (viral infections, physical trauma, physical and mental stress, vaccinations, and environmental toxins), microbiome disruption, dysregulated immune response, chronic low-grade inflammation, neuroendocrine abnormalities, oxidative stress, metabolic dysfunction, mitochondrial dysfunction, and genetic predisposition (Brinth et al., 2019; Gregorowski et al., 2019; Noor et al., 2021). These factors can also interact to promote the occurrence and development of CFS. Some studies have suggested that infectious triggers can trigger systemic inflammation by activating the antiviral immune response (Kennedy et al., 2010; Maes et al., 2012; Glassford, 2017; Cortes Rivera et al., 2019). The composition of gut microbes is altered in CFS patients, which might lead to increased intestinal permeability that allows bacterial translocation into the bloodstream, thus increasing systemic inflammation (Deumer et al., 2021). The hypothalamic-pituitary-adrenal (HPA) axis is impaired in patients with CFS, which may result in neuroendocrine abnormalities and metabolic and inflammatory changes (Deumer et al., 2021). In addition, genetic predisposition is associated with autoimmunity (Deumer et al., 2021).

Currently, the treatment of CFS remains suboptimal because there is a lack of an adequate understanding of the mechanisms and etiology of the disease. Current recommendations for the treatment of CFS include cognitive behavioral therapy (CBT), graded exercise therapy (GET), western conventional medicine (WCM), complementary or alternative medicine, and nutritional support therapy. CBT challenges patients’ thoughts to relieve patients’ psychological stress, and this may provide short-term benefits but does not permanently reduce symptoms (Fernie et al., 2016; Geraghty and Blease, 2018). Exercise therapy, including aerobic exercises (e.g., walking, jogging, swimming, and cycling) and anaerobic exercises (e.g., strength and stability exercises), could improve physical function and reduce fatigue (Marques et al., 2015; Larun et al., 2017). However, some patients have expressed disappointment with GET because it can interfere with the outcome of alternative treatments and may indirectly exacerbate symptoms in patients (Goudsmit and Howes, 2017; Geraghty and Blease, 2019). Western conventional medicines such as immune modulators, antivirals, antidepressants, antibiotics, and medications to treat specific symptoms that are used for treating CFS have insufficient evidence for their efficacy and may cause serious adverse effects (Mücke et al., 2015; Smith et al., 2015; Yang et al., 2017). In addition, alternative medicine (e.g., meditation and relaxation response, warm baths, massages, stretching, acupuncture, hydrotherapy, chiropractic, yoga, and Tai Chi), nutritional support therapy, transcutaneous electrical nerve stimulation, physiotherapy, and nerve blocks have all been proposed, but the evidence regarding these treatments is limited and their efficacy is uncertain (Bested and Marshall, 2015; Noor et al., 2021).

Chinese herbal medicine (CHM) has been widely used to treat CFS in China and other parts of the world, such as South Korea and Japan (Wang et al., 2014; Joung et al., 2019; Shin et al., 2021). First, according to the dialectical treatment theory of traditional Chinese medicine, specific formulas consisting of different Chinese herbs are used to treat CFS patients with different symptoms. Such treatment tailored to the patient’s specific needs is urgently needed given the obvious heterogeneity in CFS symptoms. The pathogenesis of CFS in traditional Chinese medicine is the deficiency of qi, blood, and yin and yang, accompanied by the stagnation of qi, fire, phlegm, and blood. The treatment is focused on tonifying deficiencies and relieving bruising. CHM such as *Panax ginseng* C.A.Mey., *Codonopsis pilosula* (Franch.) Nannf., and *Astragalus mongholicus* Bunge can nourish deficiency and improve fatigue and lengthy malaise after exertion in CFS patients, whereas *Bupleurum falcatum* L. and *Citrus* × *aurantium* L., among others, can resolve stagnation and relieve pain, insomnia, swollen lymph nodes, and other symptoms. Therefore, CHM can not only improve the main symptoms, but also relieve the accompanying symptoms in CFS. Second, modern pharmacological research has demonstrated that the modern use of CHM in treating CFS mainly focuses on adjusting immune dysfunction, acting as an antioxidant, improving the energy metabolism disorder, and regulating abnormal activity in the HPA axis (Chen et al., 2010; Chi et al., 2016). Buzhong Yiqi decoction, Kuibi decoction, Danggui Buxue decoction, Young Yum pill, and Renshen Yangrong decoction can regulate the immune function of patients with CFS and relieve fatigue symptoms (Ogawa et al., 1992; Shin et al., 2004; Chen et al., 2010; Yin et al., 2021; Miao et al., 2022). Ginsenoside, Jujube polysaccharide conjugate, Quercetin, *Withania somnifera* (L.) Dunal, *Hypericum perforatum* L., and *Ginkgo biloba* L. can be antioxidants (Logan and Wong, 2001; Singh et al., 2002; Chi et al., 2015). Schisandra Chinensis Polysaccharide (SCP), HEP2-a extracted from *Epimedium brevicornum* Maxim., can improve energy metabolism and can regulate the abnormal activity of the HPA axis (Chi et al., 2016; Chi et al., 2017). Additionally, multiple randomized controlled trials (RCTs) have reported that CHM significantly improves fatigue, insomnia, and other concomitant symptoms; reduces negative emotions such as anxiety and depression; and clearly improves treatment effectiveness and quality of life compared to exercise therapy and alternative therapy (Wang et al., 2011; Kong, 2012; Wang, 2021). Systematic reviews and meta-analyses comparing CHM with western medicine also confirmed the above views (Peng et al., 2013; Wang et al., 2014). These studies demonstrate the remarkable efficacy and comprehensiveness of CHM for CFS, which is consistent with treatment guidelines emphasizing a holistic, patient-centered approach that considers the patient’s physical, mental, and social well-being (Baker and Shaw, 2007). Finally, CHM has no serious side effects and is relatively safe to treat CFS.

A previous meta-analysis and another systematic review indicated the beneficial role of CHM as a complementary approach for CFS (Peng et al., 2013; Wang et al., 2014). However, those studies were limited in terms of sample size and outcome indicators because the systematic review only assessed 10 RCTs (including 919 patients), and the meta-analysis of 11 RCTs (including 1,049 patients) only assessed clinical efficacy rates and lacked sufficient evidence. In addition, nearly 50 new trials assessing the effects of CHM for CFS have been published since the previous systematic reviews and meta-analyses were published. Therefore, we conducted a larger systematic review and meta-analysis including more outcome indicators (FS-14, FAI, SCL-90, SAS, SDS, clinical symptom scores, IGA, IGG, IGM, NK cell levels, effective rate, and adverse events) to provide a comprehensive update of previously published studies and stronger evidence for the effectiveness of CHM for CFS.

## Methods

### Protocol and registration

This meta-analysis was reported in compliance with the PRISMA statement, and the protocol was registered on PROSPERO (CRD42022319680). [https://www.crd.york.ac.uk/prospero/display_record.php?ID=CRD42022319680]. The full details of the protocol are available on request.

### Search strategy

Electronic databases including PubMed, Embase, Cochrane Library, Web of Science, the Chinese National Knowledge Infrastructure (CNKI), Wanfang Database, Chinese VIP Database, the US Clinical Trials Registry, and the Chinese Clinical Trials Registry were systematically searched from their inception to May 2022. There was no restriction on language. The search terms used included “Fatigue Syndrome, Chronic”, “CFS”, “Chronic Fatigue Syndrome”, “Myalgic Encephalomyelitis”, “ME”, “Encephalomyelitis, Myalgic”, “Chronic Fatigue Disorder”, “Fatigue Disorder, Chronic”, “Systemic Exertion Intolerance Disease”, “Chinese herbal medicine”, “Chinese traditional”, “Oriental traditional”, “traditional Chinese medicine”, “traditional Chinese medicinal materials”, “Chinese herb”, “herbal medicine”, “herbal”, “decoction”, “tang”, “pill”, “wan”, “powder”, “formula”, “granule”, “capsule”, “particles”, “ointment”, “prescription”, “receipt”, “placebo”, “random controlled trial”, “random”, and “RCT”. The full details of the search strategy are available (Additional file 1). In addition, we performed manual searches in the reference lists of previously published systematic reviews and meta-analyses on the subject to further look for potentially eligible studies. The search was conducted independently by two authors (YZ and WS).

### Eligibility criteria

#### Types of studies

RCTs assessing the efficacy and safety of CHM in the treatment of CFS were included in our review. We only extracted data from the CHM and control groups when we found relevant studies with three treatment groups.

### Types of participants

Trials of participants over the age of 16 were included regardless of gender, culture, or setting. CFS was diagnosed using the Center for Disease Control criteria (1987, 1994, or 1998), the Guiding Principles for Clinical Research of New Chinese Medicines (2002), Chinese medicine internal disease diagnosis and treatment routines, the clinical research guidelines for new Chinese medicines for CFS, Chinese internal medicine diagnoses, or the diagnostic efficacy criteria for Chinese medical evidence. All patients had the primary symptom of unexplained fatigue that lasted at least 6 months accompanied by four or more of the following symptoms: unrefreshing sleep, lengthy malaise after exertion, impairment of concentration or short-term memory, sore throat, tender lymph nodes, multi-joint pain, and headaches.

### Types of interventions

The formulations of CHM were included. CHM is defined as medicinal raw materials derived from medicinal plants, minerals, and animal sources, according to the Chinese Pharmacopoeia edited in 2020 (Chinese Pharmacopoeia Commission, 2020). A formulation of CHM is usually made up of two or more herbs to produce a synergistic effect on specific illnesses. These materials are prescribed by doctors based on the individual characteristics of the patient according to the dialectical treatment theory of traditional Chinese medicine (Xiong et al., 2019; Chinese Pharmacopoeia Commission, 2020).

Participants were treated with CHM alone or combined with WCM, GET, or health guidance. We did not place any limits on the formulation of CHM or the duration of treatment, but CHM was required to be taken orally. We did not include experiments combining Chinese herbal medicine with other traditional Chinese medicine treatments.

### Types of controls

Patients in the control group used WCM, GET, health guidance, or placebo, with no limit on the duration of treatment. We did not include experiments combining any Chinese medicine therapy.

### Types of outcome measures

The primary outcome measures were Fatigue Scale (FS-14) and Fatigue Assessment Instrument (FAI) scores. The secondary outcome measures were Self-Rating Scale of mental state (SCL-90) scores, Self-Rating Anxiety Scale (SAS) scores, Self-Rating Depression Scale (SDS) scores, clinical symptom scores, immunological indicators (IGA, IGG, IGM, and NK cell levels), effective rate, and adverse events.

The clinical symptom scores are used to assess the severity of fatigue. The main symptoms and other symptoms of CFS are scored according to their severity, and a higher cumulative score of all symptoms indicates more severe fatigue symptoms. The effective rate is a measure to assess clinical efficacy. It is assessed at the end of treatment using four grades: clinical cure (the patients’ clinical symptoms were basically cured, and they could live and work normally), markedly effective (the cure rate for major and concomitant clinical symptoms up to 2/3), effective (the cure rate for major and concomitant clinical symptoms is 1/3 to 2/3), and invalid (the cure rate for major and concomitant clinical symptoms <1/3).

### Study selection

Study selection was performed independently by two authors (YZ and FJ) according to the inclusion criteria. After eliminating duplicates, they independently scanned the title/abstract and full text to identify eligible studies. Any disagreements were settled by a discussion with a third evaluator (WS).

### Data extraction

Two investigators (YZ and XW) independently reviewed and extracted the following information: general information (first author, year of publication, region, and types), characteristics of the participants (sample size, age, gender, and course of disease), details of the intervention and the comparison (type of intervention and duration), and outcomes. Any discrepancies were resolved by discussions or adjudication by a third reviewer (YP).

### Quality assessment

The risk of bias in the included studies was evaluated independently by two authors (XW and FJ) using the Cochrane collaboration tool with the following seven domains: random sequence generation (selection bias), allocation concealment (selection bias), blinding of participants and personnel (performance bias), blinding of outcome assessment (detection bias), incomplete outcome data (attrition bias), selective reporting (reporting bias), and other bias. Each domain can be classified as “low-risk,” “high-risk,” or “uncertain risk.” Any differences were resolved by discussion with a third evaluator.

### Statistical analysis

Statistical analysis was performed using the Stata software (version 12.0; StataCorp, College Station, TX). The weighted mean difference (WMD) for continuous variables and the risk ratio (RR) for dichotomous data with 95% confidence intervals (Cl) were used. Heterogeneity was assessed by the Q test and the I^2^ statistic. When *p* ≥ 0.10 and I^2^ ≤ 50%, the fixed-effect model was used; otherwise, the random effects model was used. *p* ≤ 0.05 was considered statistically significant. The publication bias was assessed by funnel plots and Egger’s test if the number of trials was sufficient. When heterogeneity was detected, the sensitivity analysis was conducted to assess the stability of the results by excluding individual studies one by one. Subgroup analysis was performed to explore the sources of heterogeneity according to treatment method (CHM vs. WCM, CHM plus WCM vs. WCM, CHM vs. GET, CHM plus GET vs. GET, CHM vs. health guidance, and CHM vs. placebo) and duration of the intervention (≤30 days vs. 31–60 days vs. > 60 days) based on different treatment methods.

## Results

### Literature search

**FIGURE 1:**
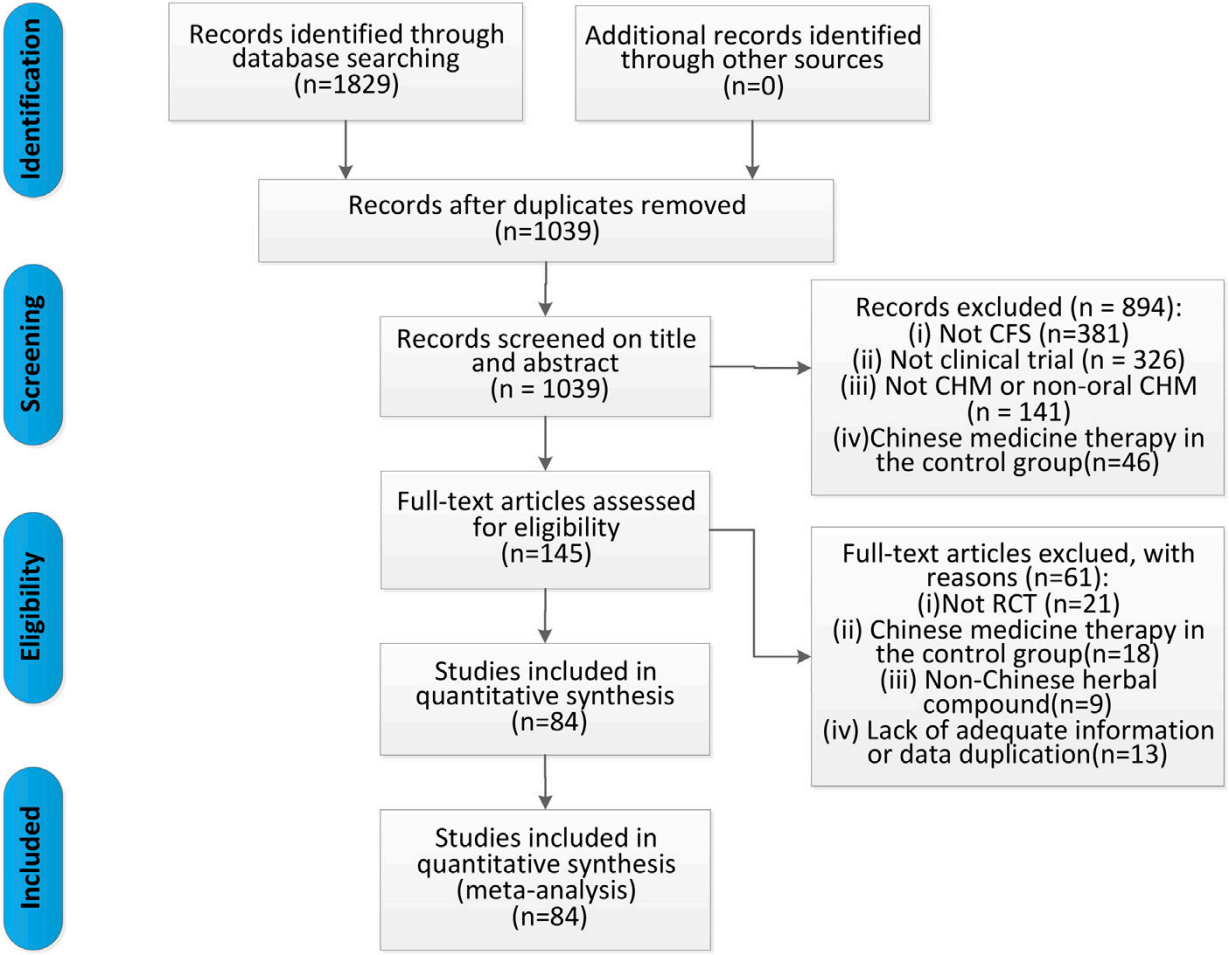
Flow diagram of the studies selection process.

We identified 1,829 articles in the original screening. After eliminating duplicates, 1,039 remained, 894 of which were excluded because they did not meet the inclusion criteria after scanning the titles and abstracts. Moreover, we reviewed the full text of the remaining 145 articles and deleted 61 articles due to the following reasons: 1) non-RCTs, 2) Chinese medicine therapy used in the control group, 3) non-Chinese herbal compounds used, 4) published using repeated data, and 5) missing data. Finally, 84 articles were included in the meta-analysis (Figure 1).

### Study characteristics and quality assessment

A total of 84 RCTs were included, published from 2002 to 2022, and all studies were conducted in China. The sample sizes in the studies varied from 38 to 230 patients, with a total sample size of 3,552 patients in the treatment groups and 3,392 patients in the control groups. The duration of diseases lasted from 0.5 to 24.27 years. Of the 84 studies, five trials (Li, 2009; Liu J. et al., 2019; Wang, 2020; Liu et al., 2021; Sheng et al., 2022) compared CHM with placebo, whereas comparisons of CHM alone vs. WCM were performed in 63 studies (Ning and Li, 2002; Yang et al., 2004; Zhang et al., 2004; Zhang and Zhou, 2004; Wei, 2005; Yao and Qiu, 2005; Liang, 2006; Zhao et al., 2006; Fang et al., 2007; Gong, 2007; Lin, 2007; Sun et al., 2007; Wang et al., 2007; Fang et al., 2008; Cheng, 2009; Ma, 2009; Zhang et al., 2009; Hu et al., 2010; Chen et al., 2011; Li et al., 2011; Liu et al., 2011; Zhang et al., 2011; Zhang Z. X. et al., 2012; Zhang L. P. et al., 2012; Jiang, 2012; Tian and Wang, 2012; Wang, 2012; Wu et al., 2012; Zhao, 2012; Lai and Lei, 2013; Pang and Liu, 2013; Xu et al., 2013; Zhao, 2013; Zhao et al., 2013; Teng et al., 2014; Xu, 2014; Li, 2015; Li and Zao, 2015; Liu et al., 2015; Niu et al., 2015; Tan et al., 2015; Zhang et al., 2015; Shi and Wu, 2016; Wu et al., 2016; Du, 2018; Luo, 2018; Ou et al., 2018; Weng, 2018; Wu et al., 2018; Liu F. et al., 2019; Liu Y. et al., 2019; Ding, 2019; He, 2019; Hu, 2019; Ma et al., 2019; Shi, 2019; Wang, 2019; Yang, 2019; Dong, 2020; Li, 2020; Mao, 2020; Chen, 2021; Zhang, 2021). CHM plus WCM vs. WCM was compared in 12 studies (Guo et al., 2007; Jie and Wang, 2009; Ren and Yu, 2012; Xu and Wang, 2013; Gao and Pang, 2015; Gao and Pang, 2016; Sun et al., 2016; Wang, 2017; Zheng et al., 2017; Li et al., 2018; Liu and Cai, 2018; Li et al., 2021); CHM vs. GET was compared in one study (Wang, 2021); CHM plus GET vs. GET was compared in two studies (Wang et al., 2011; Kong, 2012); and CHM vs. health guidance was compared in one study (Ye, 2017). The course of treatment ranged from 7 to 120 days. In the outcome indicators, 26 studies (Jie and Wang, 2009; Chen et al., 2011; Li et al., 2011; Liu et al., 2011; Zhang et al., 2011; Zhang L. P. et al., 2012; Xu et al., 2013; Xu and Wang, 2013; Liu et al., 2015; Niu et al., 2015; Tan et al., 2015; Zhang et al., 2015; Ye, 2017; Zheng et al., 2017; Du, 2018; Luo, 2018; Liu F. et al., 2019; Liu J. et al., 2019; He, 2019; Shi, 2019; Mao, 2020; Wang, 2020; Chen, 2021; Li et al., 2021; Liu et al., 2021; Wang, 2021) reported FS-14 scores; nine studies (Zhao et al., 2006; Zhang et al., 2009; Liu et al., 2011; Wu et al., 2012; Liu et al., 2015; Luo, 2018; Wang, 2019; Li et al., 2021; Wang, 2021) reported FAI scores; five studies (Zhang et al., 2009; Zhang Z. X. et al., 2012; Wu et al., 2012; Niu et al., 2015; Sheng et al., 2022) reported SCL-90 scores; seven studies (Jie and Wang, 2009; Xu and Wang, 2013; Sun et al., 2016; Ye, 2017; Yang, 2019; Liu et al., 2021; Zhang, 2021) reported SAS scores; six studies (Jie and Wang, 2009; Xu and Wang, 2013; Sun et al., 2016; Ye, 2017; Liu et al., 2021; Zhang, 2021) reported SDS scores; 24 studies (Zhang et al., 2004; Yao and Qiu, 2005; Fang et al., 2007; Wang et al., 2007; Fang et al., 2008; Li, 2009; Hu et al., 2010; Wang, 2012; Zhao, 2012; Li, 2015; Li and Zao, 2015; Sun et al., 2016; Wu et al., 2016; Ye, 2017; Du, 2018; Liu and Cai, 2018; Luo, 2018; Liu J. et al., 2019; He, 2019; Hu, 2019; Shi, 2019; Dong, 2020; Wang, 2020; Liu et al., 2021) reported clinical symptom scores; eight studies (Zhang et al., 2009; Liu et al., 2011; Jiang, 2012; Wu et al., 2012; Du, 2018; Wu et al., 2018; Liu J. et al., 2019; He, 2019) reported the level of IGA, IGG, and IGM; three studies (Zhang et al., 2009; Wu et al., 2012; Sheng et al., 2022) reported the NK cell levels; and 79 studies (Ning and Li, 2002; Yang et al., 2004; Zhang et al., 2004; Zhang and Zhou, 2004; Wei, 2005; Yao and Qiu, 2005; Liang, 2006; Zhao et al., 2006; Fang et al., 2007; Gong, 2007; Guo et al., 2007; Lin, 2007; Sun et al., 2007; Wang et al., 2007; Fang et al., 2008; Cheng, 2009; Jie and Wang, 2009; Li, 2009; Ma, 2009; Zhang et al., 2009; Hu et al., 2010; Chen et al., 2011; Li et al., 2011; Liu et al., 2011; Wang et al., 2011; Zhang et al., 2011; Jiang, 2012; Kong, 2012; Ren and Yu, 2012; Tian and Wang, 2012; Wang, 2012; Zhang Z. X. et al., 2012; Zhang L. P. et al., 2012; Zhao, 2012; Lai and Lei, 2013; Pang and Liu, 2013; Xu et al., 2013; Xu and Wang, 2013; Zhao, 2013; Zhao et al., 2013; Teng et al., 2014; Xu, 2014; Gao and Pang, 2015; Li and Zao, 2015; Li, 2015; Tan et al., 2015; Zhang et al., 2015; Gao and Pang, 2016; Shi and Wu, 2016; Sun et al., 2016; Wu et al., 2016; Wang, 2017; Ye, 2017; Zheng et al., 2017; Du, 2018; Li et al., 2018; Liu and Cai, 2018; Ou et al., 2018; Weng, 2018; Wu et al., 2018; Luo, 2018; Ding, 2019; He, 2019; Hu, 2019; Liu F. et al., 2019; Liu J. et al., 2019; Liu Y. et al., 2019; Ma et al., 2019; Shi, 2019; Wang, 2019; Yang, 2019; Dong, 2020; Li, 2020; Mao, 2020; Wang, 2020; Chen, 2021; Li et al., 2021; Liu et al., 2021; Wang, 2021) reported effective rate. The occurrence of adverse effects was reported in 14 studies (Liang, 2006; Gong, 2007; Lin, 2007; Wang et al., 2007; Jie and Wang, 2009; Li, 2009; Li et al., 2011; Xu and Wang, 2013; Zhang et al., 2015; Sun et al., 2016; Wu et al., 2016; Ye, 2017; Li et al., 2018; Liu and Cai, 2018). The basic characteristics of the included studies are summarized in Table 1, and components of CHM used in the included studies are presented in Table 2.

**TABLE 1:** Characteristics of the included studies.

Study	Region	Types	Sample size (TG/CG)	Age (Y)		Gender (M/F)		Course of disease		Interventions		Duration (days)	Outcomes
				TG	CG	TG	CG	TG	CG	TG	CG		
Ning and Li (2002)	China	RCT	43 (23/20)	42	41	7/16	5/15	0.5–5 years	0.5–5 years	Sijunzi decoction (1 package, bid)	Oryzanol + antipsychotic + vitamin	30	⑪
Yang et al. (2004)	China	RCT	72 (38/34)	36.4	36.4	NR	NR	2.5 years	2.5 years	Buzhong Yiqi decoction and Xiaochaihu decoction (qd)	ATP (2 tablets, tid)	30	⑪
Zhang et al. (2004)	China	RCT	100 (60/40)	36.2 ± 7.82	34.92 ± 10.28	26/34	18/22	0.5–5 years	0.5–5 years	Self-designed Shenqi Fuyuan decoction (200 ml, bid)	Oryzanol + multivitamin	30	⑥⑪
Zhang and Zhou (2004)	China	RCT	68 (40/28)	37	37	NR	NR	NR	NR	Buzhong Yiqi decoction	Vitamins B, B1, B6 + oryzanol + estazolam + ibuprofen sustained release capsule	42	⑪
Wei (2005)	China	RCT	72 (37/35)	29.5	28.9	12/25	10/25	2.5 years	2.4 years	Xiaochaihu decoction (1 package, bid)	Vitamin C (bid) + vitamin B (bid) + diclofenac sodium (25 mg)	21	⑪
Yao and Qiu (2005)	China	RCT	56 (31/25)	36.2 ± 7.82	35.92 ± 10.28	10/21	10/15	0.6–5 years	0.6–5 years	Self-designed Xianshen decoction (200 ml, bid)	Centrum vitaminamin A–Z (1 granule, qd)	30	⑥⑪
Liang (2006)	China	RCT	121 (63/58)	37.2	35.3	36/27	34/24	0.58–6 years	0.67–8 years	Shengmai pulvis and Xuefu Zhuyu decoction (200 ml, bid)	Vitamin C (0.1 g, tid) + vitamin B (0.2 g, tid) + ATP (20 mg, tid) + oryzanol (20 mg, tid)	28	⑪⑫
Zhao et al. (2006)	China	RCT	58 (30/28)	44	43	11/19	11/17	1.8 a	1.6 a	Yiqi Yangyin Jichu prescription (tid)	ATP (40 mg, tid) + vitamin C (0.2 g, tid)	90	②⑪
Fang et al. (2007)	China	RCT	70 (35/35)	42.15 ± 9.31	42.15 ± 9.31	NR	NR	16.58 ± 7.69 years	16.58 ± 7.69 years	Xiaopiling granule (20 g, tid)	Oryzanol (20 mg, tid)	28	⑥⑪
Gong (2007)	China	RCT	58 (30/28)	36.3	36.8	10/20	12/16	0.5–5 years	0.5–5 years	Guipi decoction (200 ml, bid)	ATP (40 mg, tid)	45	⑪⑫
Guo et al. (2007)	China	RCT	120 (60/60)	51.07 ± 7.57	49.75 ± 7.78	29/31	32/28	2.63 ± 1.16 years	2.65 ± 1.39 years	Qi and blood proral solution (1 package, 10 ml, tid) + oryzanol (20 mg, tid)	Oryzanol (20 mg, tid)	30	⑪
Lin (2007)	China	RCT	88 (50/38)	28–60	25–65	15/35	21/17	1–6 years	0.7–5 years	Shenling Baizhu powder (60–90 g, bid/tid)	Oryzanol + vitamin B1 + vitamin B6 + deanxit + amino acid	56	⑪⑫
Sun et al. (2007)	China	RCT	64 (34/30)	35.8	35.8	NR	NR	2.2 a	2.2 a	Shuyu decoction (200 ml, bid)	Vitamin C (0.1 g, tid) + vitamin B (0.2 g, tid) + oryzanol (20 mg, tid)	40	⑪
Wang et al. (2007)	China	RCT	105 (53/52)	41.37 ± 8.52	41.37 ± 8.52	NR	NR	17.51 ± 6.39 m	17.51 ± 6.39 m	Fuzheng Jieyu prescription (1 package, bid)	Oryzanol (20 mg, tid) + ATP (40 mg, tid)	28	⑥⑪⑫
Fang et al. (2008)	China	RCT	230 (120/110)	40	40	NR	NR	2.5 years	2.5 years	Fufang Shenqi ointment (10 g, bid)	ATP (3 tablets, tid) + vitamin C (2 tablets, tid)	60	⑥⑪
Cheng (2009)	China	RCT	60 (30/30)	NR	NR	13/17	12/18	0.5–2 years	0.4–2 years	Bainian Le oral liquid (15 ml, bid)	Vitamin B solution (15 ml, bid)	14	⑪
Jie and Wang (2009)	China	RCT	70 (35/35)	34.55 ± 7.45	33.95 ± 6.54	14/21	16/19	14.50 ± 4.50 m	14.20 ± 4.15 m	Xiaoyao pill (24 pills, tid) + Paroxetine (20–40 mg/d)	Paroxetine (20–40 mg/d)	56	①④⑤⑪⑫
Li (2009)	China	RCT	38 (19/19)	32.75	31.45	6/13	12/7	0.5–10 years	0.5–10 years	Anti-fatigue no. 2 decoction granule (tid)	Placebo (tid)	21	⑥⑪⑫
Ma (2009)	China	RCT	118 (78/40)	52.5	50.2	35/43	18/22	NR	NR	Guipi decoction (150 ml, tid)	Vitamin (2 tablets, tid) + oryzanol (20 mg, tid) + estazolam (1–2 mg, qn) + nimesulide (0.1 g, bid)	30	⑪
Zhang et al. (2009)	China	RCT	75 (40/35)	38.63 ± 11.49	38.66 ± 10.94	19/21	14/21	10.95 ± 3.73 m	10.80 ± 2.95 m	Lixu Jieyu prescription (200 ml, bid)	Vitaeamphor (10 mg, bid) + ATP (20 mg, tid) + oryzanol (20 mg, tid)	90	②③⑦⑧⑨⑩⑪
Hu et al. (2010)	China	RCT	120 (60/60)	41.23	41.23	NR	NR	NR	NR	Buqi Tongluo prescription (1 package, tid)	Vitamin B complex (5 ml, tid)	NR	⑥⑪
Chen et al. (2011)	China	RCT	80 (40/40)	44.7 ± 5.6	47.4 ± 3.4	16/24	18/22	0.5–4 years	0.5–3.5 years	Qixue Liangxu prescription + Ganyu Pixu prescription + Ganshen Kuixu prescription	Multidimensional tablet (10 mg, bid) + meloxicam (1 tablet, qd) + estazolam tablet (1 mg, qd) + flupentixton melitoxin (1 tablet, bid)	90	①⑪
Li et al. (2011)	China	RCT	71 (36/35)	38.36 ± 7.16	39.22 ± 6.85	16/20	17/18	0.5–3 years	0.6–3 years	Chaihu Shugan pulvis and Guipi decoction (1 package, bid)	Oryzanol (20 mg, tid) + ATP (20 mg, tid)	56	①⑪⑫
Liu et al. (2011)	China	RCT	80 (42/38)	42.3 ± 10.6	41.2 ± 9.5	20/22	19/19	0.5–3 years	0.67–3 years	Shugan Yangxue prescription (1 package, bid)	Vitamin C (0.1 g) + vitamin B (0.2 g) + ATP (20 mg) + oryzanol (20 mg, tid)	56	①②⑦⑧⑨⑪
Wang et al. (2011)	China	RCT	96 (48/48)	39	38.5	27/21	25/23	5 years	5 years	Yiqi Ziyin Buyang prescription (300 ml, bid) + GET	GET	60	⑪
Zhang et al. (2011)	China	RCT	64 (32/32)	37.97 ± 10.35	38.66 ± 11.03	15/17	14/18	11.30 ± 4.73 m	10.98 ± 4.26 m	Zhenqi Jiepi decoction (100 ml, bid)	Gold theragran (1 tablet, tid) + ATP (20 mg, tid) + oryzanol (20 mg, tid)	60	①⑪
Jiang (2012)	China	RCT	70 (35/35)	37	38	17/18	18/17	0.67 years	1.67 years	Buzhong Yiqi decoction and Guipi decoction (1 package, bid)	Vitamin C + vitamin B complex + oryzanol	56	⑦⑧⑨⑪
Kong (2012)	China	RCT	60 (30/30)	NR	NR	NR	NR	0.5–3 years	0.5–3 years	Self-designed anti-fatigue decoction (200 ml, tid) + GET	GET	45	⑪
Ren and Yu. (2012)	China	RCT	80 (40/40)	42	44	22/18	24/16	5 a	5.6 a	Self-designed Zhongyao Buxu decoction (1 package) + oryzanol (2 tablets, tid)	Oryzanol (2 tablets, tid)	21	⑪
Tian and Wang. (2012)	China	RCT	64 (32/32)	28–60	25–65	15/17	21/11	1–6 years	0.6–3 years	Buzhong Yiqi decoction (1 package, bid)	Oryzanol + vitamin B1 + vitamin B6 + deanxit + amino acid	56	⑪
Wang. (2012)	China	RCT	84 (42/42)	40.65 ± 12.25	40.65 ± 12.25	NR	NR	2.14士1.07 years	2.14士1.07 years	Buzhong Yiqi decoction and Xiaochaihu decoction	ATP (2 tablets, tid)	28	⑥⑪
Wu et al. (2012)	China	RCT	120 (60/60)	NR	NR	24/36	22/38	≥6 m	≥6 m	Lixu Jieyu prescription (150 ml, bid)	Vitaeamphor (1 tablet, tid) + ATP (20 mg, bid) + oryzanol (20 mg, tid)	84	②③⑦⑧⑨⑩
Zhang et al. (2012a)	China	RCT	66 (33/33)	38.74 ± 11.39	39.45 ± 10.97	NR	NR	10.94 ± 3.72 years	10.81 ± 2.97 years	Lixu Jieyu prescription (200 ml, bid)	Vitaeamphor (10 mg, bid) + ATP (20 mg, tid) + oryzanol (20 mg, tid)	84	③⑪
Zhang et al. (2012b)	China	RCT	60 (30/30)	37.16+-9.93	37.77+-11.48	13/17	12/18	12.52 ± 5.18 m	13.35 ± 5.17 m	Yaoyao Xiaopi prescription (100 ml, bid)	Gold theragran (1 tablet, tid) + ATP (20 mg, tid) + oryzanol (20 mg, tid)	60	①⑪
Zhao. (2012)	China	RCT	84 (42/42)	40.65 ± 12.25	40.65 ± 12.25	NR	NR	2.14 ± 1.07 years	2.14 ± 1.07 years	Buzhong Yiqi decoction and Xiaochaihu decoction	ATP (2 tablets, tid)	NR	⑥⑪
Lai and Lei. (2013)	China	RCT	68 (34/34)	46.8	47.6	15/19	14/20	0.6–3 years	0.5–3.5 years	Baiyu Jianpi decoction (200 ml, bid)	Oryzanol (20 mg, tid) + ATP (20 mg, tid)	56	⑪
Pang and Liu. (2013)	China	RCT	60 (32/28)	28–53	24–55	9/23	11/17	0.5–4 years	0.58- y	Shengmai pulvis and Xiaoyao pulvis	Vitamin C (0.2 g, tid) + vitamin Bco (2 tablets, tid) + ATP (20 mg, tid) + oryzanol (20 mg, tid)	28	⑪
Xu et al. (2013)	China	RCT	68 (40/28)	33.24 ± 1.56	30.24 ± 1.28	22/18	16/12	24.24 ± 4.30 m	22.20 ± 3.24 m	Jiawei Naoxin Kang (100 ml, bid)	ATP (1 tablet, bid)	10	①⑪
Xu and Wang. (2013)	China	RCT	84 (42/42)	35.29 ± 6.18	34.87 ± 7.08	17/25	19/23	15.06 ± 4.80 m	14.75 ± 5.02 m	Chaihu Jia Longgu Muli decoction (200 ml, bid) + paroxetine (20–40 mg/d)	Paroxetine (20–40 mg/d)	NR	①④⑤⑪⑫
Zhao (2013)	China	RCT	176 (88/88)	52.5	50.2	35/53	29/59	≥6 m	≥6 m	Self-designed Baihe Yangxin Jianpi decoction (175 ml, bid)	Vitamina (NR) + oryzanol (NR) + vitamin B (NR)	NR	⑪
Zhao et al. (2013)	China	RCT	90 (45/45)	36.5	35.6	NR	NR	9.35 ± 2.13 m	9.05 ± 3.13 m	Compound of Fufangteng mixture (15 ml, bid)	Vitaeamphor (10 mg, bid) + ATP (20 mg, tid) + oryzanol (20 mg, tid)	90	⑪
Teng et al. (2014)	China	RCT	60 (30/30)	43	43	12/18	11/19	2.4 years	2.7 years	Buzhong JiePi decoction (1 package, bid)	Oryzanol (10 mg, tid) + vitamin B1 tablet (10 mg, tid)	56	⑪
Xu (2014)	China	RCT	63 (32/31)	NR	NR	18/14	16/15	NR	NR	Qingshu Yiqi decoction (1 package)	Oryzanol diazepam tablet (NR) + poly methamphetamine tablet (NR)	7	⑪
Gao and Pang. (2015)	China	RCT	70 (35/35)	32.8 ± 10.5	33.6 ± 12.7	12/23	15/20	0.75–4 years	0.7–4.2 years	Wendan decoction and Sini decoction (1 dose/d, bid) + fluoxetine hydrochloride capsules (20–40 mg, qod)	Fluoxetine hydrochloride capsule (20–40 mg, qod)	28	⑪
Li and Zao. (2015)	China	RCT	74 (37/37)	55.3 ± 6.2	54.7 ± 6.9	NR	NR	≥6 m	≥6 m	Jianpi Wenshen Shugan prescription (1 dose/d, tid)	Multivitamin tablet (1 tablet, tid) + oryzanol (1 tablet, tid)	30	⑥⑪
Liu et al. (2015)	China	RCT	100 (51/49)	43.2 ± 12.6	42.6 ± 10.5	20/31	19/30	0.5–3 years	0.8–3 years	Shugan Yangxue decoction (1 dose/d, bid)	Vitamin C (0.1 g, tid) + vitamin B (0.2 g, tid) + ATP (20 mg, tid) + oryzanol (20 mg, tid)	42	①②
Li (2015)	China	RCT	68 (34/34)	41.3 ± 2.3	42.3 ± 2.4	18/16	19/15	2.4 ± 1.1 y	2.5 ± 1.2 years	Buzhong Yiqi decoction and Xiaochaihu decoction (1 dose/d, bid)	ATP (2 tablets, tid)	28	⑥⑪
Niu et al. (2015)	China	RCT	132 (66/66)	44.18 ± 8.66	46.34 ± 9.39	26/40	22/44	≥6 m	≥6 m	Bushen Shugan decoction (1 dose/d, bid)	ATP (20 mg, bid) + oryzanol (20 mg, tid)	56	①③
Tan et al. (2015)	China	RCT	60 (30/30)	35.6 ± 9.7	35.0 ± 10.4	14/16	13/17	1.4 ± 0.7 years	1.1 ± 0.5 years	Sini decoction and Wulin powder (1 dose/d, 100 ml, bid)	Vitamin B1 tablet (10 mg, tid) + vitamin B6 tablet (20 mg, tid) + oryzanol tablet (20 mg, tid)	28	①⑪
Zhang et al. (2015)	China	RCT	172 (88/84)	32 ± 6.38	33 ± 7.26	34/54	31/53	NR	NR	Wenzhen Yunqi prescription (1 package, bid)	Deanxit (2 tablets, bid)	NR	①⑪⑫
Gao and Pang. (2016)	China	RCT	70 (35/35)	32.8 ± 10.5	33.6 ± 12.7	12/23	15/20	0.75–4 years	0.7–4.2 years	Wendan decoction and Sini powder (1 dose/d, 150 ml, bid) + fluoxetine hydrochloride capsule (20–40 mg, qod)	Fluoxetine hydrochloride capsule (20–40 mg, qod)	28	⑪
Shi and Wu. (2016)	China	RCT	120 (60/60)	45.25 ± 9.81	43.14 ± 8.35	22/38	25/35	1.20 ± 0.45 years	1.15 ± 0.50 years	Suanzaoren decoction (1 dose/d)	Oryzanol (30 mg, tid)	14	⑪
Sun et al. (2016)	China	RCT	80 (40/40)	36.58 ± 5.48	36.87 ± 6.58	NR	NR	18.56 ± 6.45 m	17.75 ± 5.92 m	Shugan Yiyang capsule (0.75 g, tid) + paroxetine hydrochloride tablets (20 mg, 1 dose/d)	Paroxetine hydrochloride tablet (20 mg, 1 dose/d)	NR	④⑤⑥⑪⑫
Wu et al. (2016)	China	RCT	80 (42/38)	40.15 ± 8.51	41.46 ± 7.94	28/14	25/13	1.32 ± 0.67 years	1.28 ± 0.59 years	Xiaopi Yin (1 dose/d, 200 ml, tid)	Vitamin B6 (2 tablets, 1 dose/d)	42	⑥⑪⑫
Wang (2017)	China	RCT	140 (70/70)	42.47 ± 12.46	42.33 ± 17.40	38/32	39/31	12.63 ± 4.11 m	12.78 ± 4.24 m	Bupi Yishen decoction (1 dose/d, bid) + ATP (60 mg, tid) + oryzanol (20 mg, tid)	ATP (60 mg, tid) + oryzanol (20 mg, tid)	30	⑪
Ye (2017)	China	RCT	76 (37/39)	40.19 ± 8.05	37.67 ± 7.30	12/25	9/30	13.46 ± 4.25 m	15.13 ± 4.60 m	Shenxian congee (1 dose/d, qd) + health guidance	Health guidance	56	①④⑤⑥⑪⑫
Zheng et al. (2017)	China	RCT	90 (45/45)	35.8 ± 7.6	34.9 ± 8.1	21/24	18/27	15.4 ± 3.8 m	16.2 ± 3.5 m	Shugan Jianpi Yishen prescription (1 dose/d, bid) + paroxetine hydrochloride tablet (20 mg, qd)	Paroxetine hydrochloride tablet (20 mg, qd)	56	①⑪
Du (2018)	China	RCT	108 (54/54)	40.59 ± 5.60	40.64 ± 5.81	26/29	26/28	2.08 ± 0.57 years	2.10 ± 0.5 years	Self-designed Yishen Buxue ointment (150–200 ml, bid)	Vitamin C (0.1 g, tid) + vitamin B (0.2 g, tid) + oryzanol (20 mg, tid) + ATP (20 mg, tid)	42	①⑥⑦⑧⑨⑪
Li et al. (2018)	China	RCT	60 (30/30)	42.65 ± 8.42	42.12 ± 7.86	18/12	17/13	2.26 ± 0.67 years	2.12 ± 0.76 years	Yiqi Yangxue Bupi Hegan prescription (150 ml, bid) + paroxetine hydrochloride tablet (20–40 mg, 1 dose/d)	Paroxetine hydrochloride tablet (20–40 mg, 1 dose/d)	15	⑪⑫
Liu and Cai. (2018)	China	RCT	82 (41/41)	34.65 ± 6.98	32.99 ± 6.47	17/24	15/26	14.24 ± 4.66 m	16.01 ± 5.23 m	Bupiwei Xieyinhuo Shengyang decoction (1 dose/d, bid) + fluoxetine hydrochloride capsule (20 mg, qd)	Fluoxetine hydrochloride capsule (20 mg, qd)	28	⑥⑪⑫
Ou et al. (2018)	China	RCT	80 (40/40)	50.3 ± 11.35	49.8 ± 10.45	18/22	19/21	2–5 years	2–6 years	Guipi decoction Jiawei (1 dose/d, bid)	Fluoxetine hydrochloride capsule (20–40 mg, qod)	90	⑪
Weng (2018)	China	RCT	150 (75/75)	40.9 ± 8.9	41.7 ± 9.2	28/47	26/49	≥6 m	≥6 m	Liujunzi decoction	Oryzanol (10–20 mg, tid) + Vitamin B1 (20 mg, tid)	90	⑪
Wu et al. (2018)	China	RCT	86 (43/43)	39.7 ± 6.9	40.3 ± 7.5	16/27	18/25	0.5–5 years	0.5–7 years	Guipi decoction (1 dose/d, bid)	Vitamin C + Vitamin B	84	⑦⑧⑨⑪
Luo (2018)	China	RCT	56 (28/28)	28.14	28.86	15/13	14/14	NR	NR	Qingre Qushi prescription (1 package, bid)	Oryzanol tablet (2 tablets, tid) + multivitamin B tablet (2 tablets, tid)	14	①②⑥⑪
Ding (2019)	China	RCT	60 (30/30)	39.21 ± 1.25	41.15 ± 1.29	15/15	16/14	NR	NR	Guipi decoction (1 dose/d, bid)	Vitamin C + vitamin B	84	⑪
He (2019)	China	RCT	65 (33/32)	33.84 ± 4.98	33.70 ± 4.02	9/23	8/22	12.66 ± 3.16 m	12.57 ± 3.35 m	Shugan Jianpi Huoxue prescription (1 dose/d, bid)	Oryzanol (20 mg, tid)	28	①⑥⑦⑧⑨⑪
Hu (2019)	China	RCT	66 (33/33)	55.14 ± 1.26	55.11 ± 1.22	16/17	17/16	3.15 ± 1.14 years	3.11 ± 1.11 years	Buzhong Yiqi and Xiaochaihu decoction (1 dose/d, bid)	ATP (2 tablets, tid)	NR	⑥⑪
Liu et al. (2019a)	China	RCT	60 (30/30)	42	42	NR	NR	1 year	1 year	Jiawei Lingzhi pill (1 dose/d, bid)	Fluoxetine tablet (20 mg, qd)	30	①⑪
Liu et al. (2019b)	China	RCT	72 (36/36)	NR	NR	9/27	11/25	0.58–2 years	0.58–2.2 years	Chaihu Guizhi decoction grain (1 package, bid)	placebo (1 package, bid)	28	①⑥⑦⑧⑨⑪
Liu et al. (2019c)	China	RCT	60 (30/30)	43.3 ± 12.6	42.9 ± 10.6	10/20	11/19	15.0 ± 5.6 m	16.0 ± 6.3 m	Jianpi Yishen decoction (1 dose/d, bid)	Vitamin C (0.1 g, tid) + vitamin B (0.2 g, tid) + vitamin E (0.1 g, tid)	42	⑪
Ma et al. (2019)	China	RCT	80 (40/40)	45.2	43	12/28	10/30	NR	NR	Self-designed Jiawei Erxian decoction (1 dose/d, bid)	Vitamin B1 (20 mg) + oryzanol (20 mg) + Bailemen (4 tablets, tid)	60	⑪
Shi (2019)	China	RCT	160 (78/82)	41.51 ± 9.347	40.55 ± 9.775	35/43	32/50	0.9–3 years	0.7–2 years	Xiaoyao pulvis Jiawei (1 dose/d, bid)	Multivitamin B tablet (2 tablets, tid) + oryzanol (20 mg, tid)	21	①⑥⑪
Wang (2019)	China	RCT	69 (35/34)	34.67	35.34	18/17	19/15	NR	NR	Sanren decoction and Sijunzi decoction (1 dose/d, bid)	oryzanol (20 mg, tid) + multivitamin B tablet (20 mg, tid)	14	②⑪
Yang (2019)	China	RCT	40 (20/20)	38.45 ± 5.36	39.12 ± 5.21	11/9	10/10	0.5–1.5 years	0.5–1.5 years	Zuogui pill (9 g, bid)	Symptomatic treatment + anti-virus + improve immunity + anti-depression + psychotherapy	120	④⑪
Dong (2020)	China	RCT	80 (40/40)	37.68 ± 3.41	37.72 ± 3.34	26/14	23/17	1.24 ± 0.17 years	1.21 ± 0.15 years	Qingshu Yiqi decoction grain (200 ml, bid)	Nuodikang capsule (2 tablets, tid)	90	⑥⑪
Li (2020)	China	RCT	72 (36/36)	37.82 ± 6.03	39.11 ± 5.94	19/17	21/15	NR	NR	Buzhong Yiqi decoction and Xiaochaihu decoction	ATP (40 mg, tid)	30	⑪
Mao (2020)	China	RCT	59 (30/29)	39.58 ± 0.46	39.40 ± 0.37	17/13	18/11	1.26 ± 0.38 years	1.37 ± 0.22 years	Yishen Tiaodu method (1 dose/d, qod)	Oryzanol (20 mg, tid)	56	①⑪
Wang (2020)	China	RCT	90 (45/45)	47.5 ± 7.3	48.1 ± 7.6	16/29	17/28	17.2 ± 3.5 m	17.7 ± 3.8 m	Chaihu Guizhi decoction (1 dose/d, bid)	Placebo (12 g, bid)	28	①⑥⑪
Chen (2021)	China	RCT	63 (33/30)	33.8 ± 13.1	33.6 ± 13.2	15/18	14/16	18.51 ± 9.03 m	16.32 ± 8.94 m	Xiaoyao powder (1 dose/d, bid)	Oryzanol (20 mg, tid) + vitamin B1 (20 mg, tid) + ATP (20 mg, tid)	60	①⑪
Li et al. (2021)	China	RCT	79 (40/39)	41.60 ± 9.29	39.51 ± 9.79	22/18	19/20	10.98 ± 3.03 m	11.49 ± 3.60 m	Jiannao Yizhi ointment (bid) + oryzanol (20 mg, tid) + vitamin B1 (10 mg, tid)	Oryzanol (20 mg, tid) + vitamin B1 (10 mg, tid)	56	①②⑪
Liu et al. (2021)	China	RCT	72 (36/36)	NR	NR	NR	NR	NR	NR	Chaihu Guizhi decoction grain (1 package, bid)	Placebo (1 package, bid)	28	①④⑤⑥⑪
Wang (2021)	China	RCT	60 (30/30)	40.06 ± 11.51	41.23 ± 8.47	10/20	12/18	NR	NR	Shenling Baizhu powder (1 dose/d, bid)	GET	30	①②⑪
Zhang (2021)	China	RCT	60 (30/30)	45.2 ± 3.1	46.8 ± 3.4	14/16	16/14	NR	NR	Jiawei Guizhi Xinjia decoction (1 dose/d, bid)	Fluoxetine hydrochloride capsule (20–60 mg, qd)	84	④⑤
Sheng et al. (2022)	China	RCT	69 (35/34)	NR	NR	16/19	14/20	NR	NR	Wenshen Lipi prescription (1 package, bid)	Placebo (1 package, bid)	28	③⑩

RCT: randomized controlled trial; TG: trial group; CG: control group; F: female; M: male; NR: not reported; ATP: adenosine triphosphate; Y: year; GET: graded exercise therapy; ①: Fatigue Scale scores; ②: Fatigue Assessment Instrument scores; ③: Self-Rating Scale of mental state scores; ④: Self-Rating Anxiety Scale scores; ⑤: Self-Rating Depression Scale scores; ⑥: clinical symptom scores; ⑦: Immunoglobulin A; ⑧: Immunoglobulin G; ⑨: Immunoglobulin M; ⑩: natural killer cell level; ⑪: effective rate; ⑫: adverse events.

**TABLE 2:** Components of Chinese herbal medicine used in the included studies.

Study	Prescription name	Ingredients of herb prescription	Preparation
Ning and Li. (2002)	Sijunzi decoction	*Codonopsis pilosula* (Franch.) Nannf. 10 g, *Atractylodes macrocephala* Koidz. 12 g, *Poria cocos* (Schw.) Wolf 12 g, Glycyrrhiza *glabra* L. 6 g, *Astragalus mongholicus* Bunge 20 g, *Acorusgramineus* Aiton 10 g, *Polygala tenuifolia* Willd. 10 g, *Dimocarpus Longan* Lour. 10 g	Decoction
Yang et al. (2004)	Buzhong Yiqi decoction and Xiaochaihu decoction	*Codonopsis pilosula* (Franch.) Nannf. 25 g, *Astragalus mongholicus* Bunge 30 g, *Bupleurum falcatum* L. 15 g, *Agrimonia Pilosa* Ledeb 25 g, *Atractylodes macrocephala* Koidz. 20 g, *Pinellia ternata* (Thunb.) Makino 15 g, *Poria cocos* (Schw.) Wolf 20 g, *Curcuma aromatica* Salisb. 20 g, *Platycodon grandiflorus* (Jacq.) A.DC. 6 g, *Citrus × aurantium* L. 15 g, *Scutellaria baicalensis* Georgi 12 g, Glycyrrhiza *glabra* L. 12 g	Granule
Zhang et al. (2004)	Self-designed Shenqi Fuyuan decoction	*Astragalus mongholicus* Bunge, *Panax ginseng* C.A.Mey., *Atractylodes macrocephala* Koidz., *Angelica sinensis* (Oliv.) Diels, *Actaea cimicifuga* L., *Bupleurum falcatum* L., *Citrus × aurantium* L., Glycyrrhiza *glabra* L.	Decoction
Zhang and Zhou. (2004)	Buzhong Yiqi decoction	*Panax ginseng* C.A.Mey. 10 g, *Astragalus mongholicus* Bunge 12 g, *Atractylodes macrocephala* Koidz. 10 g, *Poria cocos* (Schw.) Wolf 10 g, *Angelica sinensis* (Oliv.) Diels 9 g, *Polygala tenuifolia* Willd. 9 g, Glycyrrhiza *glabra* L. 9 g, *Bupleurum falcatum* L. 9 g, *Paeonia lactiflora* Pall. 9 g, *Spatholobus suberectus* Dunn 12 g, *Citrus × aurantium* L. 10 g, *Rehmannia glutinosa* (Gaertn.) DC. 2 g	Decoction
Wei (2005)	Xiaochaihu decoction	*Bupleurum falcatum* L. 12 g, *Scutellaria baicalensis* Georgi 12 g, *Pinellia ternata* (Thunb.) Makino 12 g, *Zingiber officinale* Roscoe 10 g, *Panax ginseng* C.A.Mey. 10 g, Glycyrrhiza *glabra* L. 6 g, *Ziziphus Jujuba* Mill. 5 pieces	Decoction
Yao and Qiu. (2005)	Self-designed Xianshen decoction	*Panax ginseng* C.A.Mey. 10 g, *Paeonia lactiflora* Pall. 12 g, *Agrimonia Pilosa* Ledeb 30 g, *Panax notoginseng* (Burkill) F.H.Chen 6 g	Decoction
Liang (2006)	Shengmai pulvis and Xuefu Zhuyu decoction	*Panax ginseng C.A.Mey*. 12 g, *Ophiopogon japonicus* (Thunb.) Kergawl. 15 g, *Schisandra chinensis* (Turcz.) Baill. 10 g, *Rehmannia glutinosa* (Gaertn.) DC. 20 g, *Paeonia lactiflora* Pall. 15 g, *Angelica sinensis* (Oliv.) Diels 8 g, *Conioselinum anthriscoides* “Chuanxiong” 6 g, *Prunus Persica* (L.) Batsch 10 g, *Carthamus tinctorius* L. 10 g, *Citrus × aurantium* L. 8 g, *Platycodon grandiflorus* (Jacq.) A.DC. 10 g, *Bupleurum falcatum* L. 10 g, *Achyranthes bidentata* Blume 15 g, *Curcuma aromatica* Salisb. 20 g, Glycyrrhiza *glabra* L. 6 g	Decoction
Zhao et al. (2006)	Yiqi Yangyin Jichu prescription	*Astragalus mongholicus* Bunge 20 g, *Pseudostellaria Heterophylla* (Miq.) Pax 10 g, *Atractylodes macrocephala* Koidz. 15 g, *Poria cocos* (Schw.) Wolf 10 g, *Angelica sinensis* (Oliv.) Diels 15 g, *Paeonia lactiflora* Pall. 20 g, *Rehmannia glutinosa* (Gaertn.) DC. 10 g, *Ophiopogon japonicus* (Thunb.) Kergawl. 15 g, *Lycium chinense* Mill. 15 g, *Cornus Officinalis* Siebold & Zucc. 20 g, *Anemarrhena asphodeloides* Bunge 10 g	Decoction
Fang et al. (2007)	Xiaopiling granule	*Panax ginseng* C.A.Mey., *Astragalus mongholicus* Bunge, Equus Asinus L., *Ophiopogon japonicus* (Thunb.) Kergawl., *Dimocarpus Longan* Lour., *Angelica sinensis* (Oliv.) Diels, *Salvia miltiorrhiza* Bunge, *Ganoderma lucidum* (Leyss. ex Fr.) Karst., *Ziziphi Spinosae Semen*, *Poria cocos* (Schw.) Wolf, *Schisandra chinensis* (Turcz.) Baill., *Crataegus Pinnatifida* Bunge, *Spatholobus suberectus* Dunn	Granule
Gong (2007)	Guipi decoction	*Astragalus mongholicus* Bunge, *Codonopsis pilosula* (Franch.) Nannf., *Poria cocos* (Schw.) Wolf, *Atractylodes macrocephala* Koidz., *Polygala tenuifolia* Willd., *Dimocarpus Longan* Lour., *Dolomiaea Costus* (Falc.) Kasana and A.K.Pandey, *Agrimonia Pilosa* Ledeb, *Ziziphus Jujuba* Mill., *Ziziphi Spinosae Semen*, *Matricaria Chamomilla* L., *Strobilanthes Cusia* (Nees) Kuntze, Glycyrrhiza *glabra* L.	Decoction
Guo et al. (2007)	Qi and Blood Proral Solution	*Codonopsis pilosula* (Franch.) Nannf., *Astragalus mongholicus* Bunge, *Epimedium brevicornum* Maxim., *Atractylodes macrocephala* Koidz., *Rehmannia glutinosa* (Gaertn.) DC., *Lycium chinense* Mill., *Poria cocos* (Schw.) Wolf, *Curculigo Orchioides*gaertn., *Paeonia lactiflora* Pall., *Angelica sinensis* (Oliv.) Diels	Oral liquids
Lin (2007)	Shenling Baizhu powder	*Pseudostellaria Heterophylla* (Miq.) Pax 90 g, *Poria cocos* (Schw.) Wolf 90 g, *Euryale Ferox* Salisb. 90 g, *Nelumbo Nucifera*gaertn. 90 g, *Lablab Purpureus* Subsp. *Purpureus* 90 g, g*lycine Max* (L.) Merr. 90 g, *Lycium chinense* Mill. 90 g, *Polygonum multiflorum* Thunb. 90 g, *Dioscorea oppositifolia* L. 150 g, *Coix lacryma-jobi* L. 60 g, *Astragalus mongholicus* Bunge 60 g, *Paeonia lactiflora* Pall. 40 g, *Citrus × aurantium* L. 25 g, *Placenta Hominis* 50 g, *Ligustrum Lucidum* W.T.Aiton 50 g, *Cornus Officinalis* Siebold & Zucc. 50 g, *Oryza sativa* L. 1250 g	Decoction
Sun et al. (2007)	Shuyu decoction	*Lilium Lancifolium* Thunb. 30 g, *Anemarrhena asphodeloides* Bunge 10 g, *Triticum aestivum* L. 30 g, *Ziziphus Jujuba* Mill. 30 g, *Bupleurum falcatum* L. 10 g, *Paeonia lactiflora* Pall. 20 g, *Citrus × aurantium* L. 10 g, *Glycyrrhiza glabra* L. 10 g, *Albiziae Cortex* 30 g, *Curcuma aromatica* Salisb. 15 g, *Ziziphi Spinosae Semen* 30 g, *Codonopsis pilosula* (Franch.) Nannf. 20 g, *Atractylodes macrocephala* Koidz. 10 g	Decoction

Study	Prescription name	Ingredients of herb prescription	Preparation
Wang et al. (2007)	Fuzheng Jieyu prescription	*Angelica sinensis* (Oliv.) Diels 10 g, *Conioselinum anthriscoides* “Chuanxiong” 6 g, *Paeonia lactiflora* Pall. 12 g, *Rehmannia glutinosa* (Gaertn.) DC. 15 g, *Panax ginseng* C.A.Mey. 5 g, *Atractylodes macrocephala* Koidz. 10 g, *Poria cocos* (Schw.) Wolf 8 g, Glycyrrhiza *glabra* L. 5 g, *Dioscorea oppositifolia* L. 12 g, *Lycium chinense* Mill. 12 g, *Cornus Officinalis* Siebold & Zucc. 12 g, *Achyranthes bidentata* Blume 9 g, *Cuscuta chinensis* Lam. 12 g, *Cervus nippon* Temminck 12 g, *Colla Carapacis et Plastri Testudinis* 12 g, *Citrus × aurantium* L. 6 g, *Bupleurum falcatum* L. 6 g, *Citrus × aurantium* L. 9 g, *Atractylodes lancea* (Thunb.) DC. 6 g, *Cyperus rotundus* L. 6 g, *Hyssopus officinalis* L. 9 g, g*ardenia Jasminoides* J.Ellis 6 g	Decoction
Fang et al. (2008)	Fufang Shenqi ointment	*Panax ginseng* C.A.Mey., *Astragalus mongholicus* Bunge, *Atractylodes macrocephala* Koidz., *Poria cocos* (Schw.) Wolf, Glycyrrhiza *glabra* L., *Paeonia lactiflora* Pall., *Conioselinum anthriscoides* “Chuanxiong,” *Angelica sinensis* (Oliv.) Diels, *Rehmannia glutinosa* (Gaertn.) DC., *Curcuma aromatica* Salisb., *Bupleurum falcatum* L.	Ointment
Cheng (2009)	Bainian Le oral liquid	*Euonymus fortunei* var. *fortunei*, *Panax ginseng* C.A.Mey., *Astragalus mongholicus* Bunge, *Saccharum officinarum* L.talis (L.) Franco	Oral liquids
Jie and Wang. (2009)	Xiaoyao pill	*Bupleurum falcatum* L., *Angelica sinensis* (Oliv.) Diels, *Paeonia lactiflora* Pall., *Atractylodes macrocephala* Koidz., *Poria cocos* (Schw.) Wolf	Pill
Li (2009)	Anti-fatigue no. 2 decoction granule	*Astragalus mongholicus* Bunge, *Angelica sinensis* (Oliv.) Diels, *Platycladus orientalis* (L.) Franco, *Polygala tenuifolia* Willd., *Cyperus rotundus* L	Granule
Ma (2009)	Guipi decoction	*Panax ginseng* C.A.Mey. 10 g, *Poria cocos* (Schw.) Wolf 20 g, *Astragalus mongholicus* Bunge 30 g, *Neolitsea cassia* (L.) Kosterm. 10 g, *Atractylodes macrocephala* Koidz. 15 g, *Ziziphi Spinosae Semen* 30 g, *Dolomiaea Costus* (Falc.) Kasana and A.K.Pandey 10 g, *Angelica sinensis* (Oliv.) Dielslog 10 g, *Polygala tenuifolia* Willd. 10 g, *Actaea cimicifuga* L. 8 g, *Bupleurum falcatum* L. 10 g	Decoction
Zhang et al. (2009)	Lixu Jieyu prescription	*Astragalus mongholicus* Bunge 30 g, *Pueraria montana* var. 30 g, *Codonopsis pilosula* (Franch.) Nannf. 15 g, *Salvia miltiorrhiza* Bunge 10 g, *Panax notoginseng* (Burkill) F.H.Chen 15 g, *Epimedium brevicornu* Maxim. 10 g, *Curcuma aromatica* Salisb. 10 g, *Acorus gramineus* Aiton 10 g	Decoction
Hu et al. (2010)	Buqi Tongluo prescription	*Astragalus mongholicus* Bunge 30 g, *Panax ginseng* C.A.Mey. 5 g, *Citrus × aurantium* L. 15 g, *Bupleurum falcatum* L. 10 g, *Pheretima vulgaris* Chen 10 g, *Conioselinum anthriscoides* “Chuanxiong” 10 g	Decoction
Chen et al. (2011)	1. Qixue Liangxu prescription. 2. Ganyu Pixu prescription. 3. Ganshen Kuixu prescription	1. *Codonopsis pilosula* (Franch.) Nannf. 15 g, *Atractylodes macrocephala* Koidz. 15 g, *Astragalus mongholicus* Bunge 15 g, *Poria cocos* (Schw.) Wolf 15 g, *Angelica sinensis* (Oliv.) Diels 15 g, *Conioselinum anthriscoides* “Chuanxiong” 10 g, *Rehmannia glutinosa* (Gaertn.) DC. 15 g, *Paeonia lactiflora* Pall. 15 g, *Platycladus orientalis* (L.) Franco 12 g, *Poria cocos* (Schw.) Wolf 12 g, *Albiziae Cortex* 15 g, Glycyrrhiza *glabra* L. 6 g. 2. *Bupleurum falcatum* L. 10 g, *Citrus × aurantium* L. 10 g, *Cyperus rotundus* L. 10 g, *Angelica sinensis* (Oliv.) Diels 15 g, *Conioselinum anthriscoides* “Chuanxiong” 10 g, *Paeonia lactiflora* Pall. 15 g, *Atractylodes macrocephala* Koidz. 15 g, *Dioscorea oppositifolia* L. 15 g, *Poria cocos* (Schw.) Wolf 15 g, *Poria cocos* (Schw.) Wolf 12 g, Glycyrrhiza *glabra* L. 6 g. 3. *Anemarrhena asphodeloides* Bunge 9 g, Phellodendron amurense Rupr 9 g, *Rehmannia glutinosa* (Gaertn.) DC. 15 g, *Rehmannia glutinosa* (Gaertn.) DC. 15 g, *Dioscorea oppositifolia* L. 15 g, *Cornus Officinalis* Siebold & Zucc. 15 g, *Lycium chinense* Mill. 15 g, *Cuscuta chinensis* Lam. 15 g, *Achyranthes bidentata* Blume 15 g, *Trionyx sinensis* Wiegmann 15 g, *Salvia miltiorrhiza* Bunge 30 g, *Ziziphi Spinosae Semen* 12 g, *Reynoutria Multiflora* (Thunb.) Moldenke 30 g	Decoction
Li et al. (2011)	Chaihu Shugan pulvis and Guipi decoction	*Bupleurum falcatum* L. 12 g, *Citrus × aurantium* L. 12 g, *Conioselinum anthriscoides* “Chuanxiong” 10 g, *Cyperus rotundus* L. 12 g, *Citrus medica* L. 10 g, *Astragalus mongholicus* Bunge 15 g, *Angelica sinensis* (Oliv.) Diels 10 g, *Panax ginseng* C.A.Mey. 6 g, *Atractylodes macrocephala* Koidz. 10 g, *Poria cocos* (Schw.) Wolf 12 g, *Polygala tenuifolia* Willd. 15 g, *Ziziphi Spinosae Semen* 15 g, Glycyrrhiza *glabra* L. 10 g	Decoction
Liu et al. (2011)	Shugan Yangxue prescription	*Bupleurum falcatum* L. 10 g, *Rehmannia glutinosa* (Gaertn.) DC. 15 g, *Citrus medica* L. 10 g, *Curcuma aromatica* Salisb. 15 g, *Paeonia lactiflora* Pall. 10 g, *Conioselinum anthriscoides* “Chuanxiong” 10 g, *Angelica sinensis* (Oliv.) Diels 10 g, *Albiziae Cortex* 20 g	Decoction
Wang et al. (2011)	Yiqi Ziyin Buyang prescription	1. Tonifying qi: *Codonopsis pilosula* (Franch.) Nannf. 20 g, *Astragalus mongholicus* Bunge 30 g, *Atractylodes macrocephala* Koidz. 15 g, *Poria cocos* (Schw.) Wolf 12 g, *Angelica sinensis* (Oliv.) Diels 15 g, *Rehmannia glutinosa* (Gaertn.) DC. 15 g, *Schisandra chinensis* (Turcz.) Baill. 6 g, *Citrus × aurantium* L. 6 g, Glycyrrhiza *glabra* L. 3 g. 2. Nourishing the blood: *Angelica sinensis* (Oliv.) Diels 15 g, *Rehmannia glutinosa* (Gaertn.) DC. 15 g, *Conioselinum anthriscoides* “Chuanxiong” 12 g, *Paeonia anomala* subsp. *veitchii *(Lynch) D.Y.Hong and K.Y.Pan 12 g, *Codonopsis pilosula* (Franch.) Nannf. 15 g, *Astragalus mongholicus* Bunge 15 g, *Lycium chinense* Mill. 12 g, *Spatholobus suberectus* Dunn 15 g, Glycyrrhiza *glabra* L. 3 g. 3. Nourishing Yin: *Adenophora triphylla* (Thunb.) A.DC. 12 g, *Ophiopogon japonicus* (Thunb.) Kergawl. 12 g, *Polygonatum odoratum* (Mill.) Druce 12 g, *Rehmannia glutinosa* (Gaertn.)	Decoction

Study	Prescription name	Ingredients of herb prescription	Preparation
		DC. 12 g, *Pseudostellaria Heterophylla* (Miq.) Pax 15 g, *Schisandra chinensis* (Turcz.) Baill. 6 g, *Angelica sinensis* (Oliv.) Diels 12 g, Glycyrrhiza *glabra* L. 3 g. 4. Tonifying yang: *Rehmannia glutinosa* (Gaertn.) DC. 15 g, *Dioscorea oppositifolia* L. 12 g, *Cornus Officinalis* Siebold & Zucc. 10 g, *Lycium chinense* Mill. 12 g, *Angelica sinensis* (Oliv.) Diels 12 g, *Eucommia ulmoides* Oliv. 12 g, *Cuscuta chinensis* Lam. 12 g, *Cyperus rotundus* L. 12 g, *Neolitsea cassia* (L.) Kosterm. 3 g	
Zhang et al. (2011)	Zhenqi Jiepi decoction	*Polygala fallax* Hemsl 20 g, *Ardisia gigantifolia* Stapf 10 g, *Astragalus mongholicus* Bunge 30 g, *Codonopsis pilosula* (Franch.) Nannf. 15 g, *Atractylodes macrocephala* Koidz. 10 g, *Pueraria montana* var. 30 g, *Salvia miltiorrhiza* Bunge 15 g, *Epimedium sagittatum* (Siebold & Zucc.) Maxim. 15 g	Decoction
Jiang. (2012)	Buzhong Yiqi decoction and Guipi decoction	*Codonopsis pilosula* (Franch.) Nannf. 20 g, *Astragalus mongholicus* Bunge 20 g, *Dimocarpus Longan* Lour. 12 g, *Ziziphi Spinosae Semen* 12 g, *Atractylodes macrocephala* Koidz. 9 g, *Poria cocos* (Schw.) Wolf 9 g, *Angelica sinensis* (Oliv.) Diels 9 g, *Dolomiaea Costus* (Falc.) Kasana and A.K.Pandey 6 g, *Polygala tenuifolia* Willd. 6 g, *Citrus × aurantium* L. 6 g, *Actaea cimicifuga* L. 6 g, *Bupleurum falcatum* L. 6 g, Glycyrrhiza *glabra* L. 6 g	Decoction
Kong (2012)	Self-designed anti-fatigue decoction	*Bupleurum falcatum* L. 12 g, *Astragalus mongholicus* Bunge 25 g, *Eleutherococcus Nodiflorus* (Dunn) S.Y.Hu 18 g, *Citrus × aurantium* L. 12 g, *Paeonia lactiflora* Pall. 15 g, *Angelica sinensis* (Oliv.) Diels 15 g, *Phyllolobium Chinense* Fisch. 12 g, *Cyperus rotundus* L. 12 g, *Lycium chinense* Mill. 20 g, *Epimedium sagittatum* (Siebold & Zucc.) Maxim. 12 g, *Atractylodes macrocephala* Koidz. 15 g, *Lonicera Japonica* Thunb. 20 g	Decoction
Ren and Yu. (2012)	Self-designed Zhongyao Buxu decoction	*Astragalus mongholicus* Bunge 30 g, *Angelica sinensis* (Oliv.) Diels 20 g, *Codonopsis pilosula* (Franch.) Nannf. 20 g, *Bupleurum falcatum* L. 10 g, *Citrus × aurantium* L. 10 g, *Schisandra chinensis* (Turcz.) Baill. 10 g, *Rehmannia glutinosa* (Gaertn.) DC. 10 g, *Paeonia anomala* subsp. *veitchii* (Lynch) D.Y.Hong and K.Y.Pan 10 g, Glycyrrhiza *glabra* L. 6 g	Decoction
Tian and Wang. (2012)	Buzhong Yiqi decoction	*Astragalus mongholicus* Bunge 30 g, *Codonopsis pilosula* (Franch.) Nannf. 20 g, *Atractylodes macrocephala* Koidz. 20 g, *Poria cocos* (Schw.) Wolf 20 g, *Angelica sinensis* (Oliv.) Diels 15 g, *Curcuma aromatica* Salisb. 15 g, *Citrus × aurantium* L. 15 g, Actaea cimicifuga L.10 g, *Bupleurum falcatum* L. 15 g, *Paeonia lactiflora* Pall. 10 g, *Neolitsea cassia* (L.) Kosterm. 10 g, *Ziziphus Jujuba* Mill. 6 pieces, *Ophiopogon japonicus* (Thunb.) Kergawl. 15 g, *Schisandra chinensis* (Turcz.) Baill. 15 g, Glycyrrhiza *glabra* L. 6 g	Decoction
Wang. (2012)	Buzhong Yiqi decoction and Xiaochaihu decoction	*Codonopsis pilosula* (Franch.) Nannf. 30 g, *Astragalus mongholicus* Bunge 30 g, *Atractylodes macrocephala* Koidz. 15 g, *Poria cocos* (Schw.) Wolf 15 g, *Bupleurum falcatum* L. 12 g, *Agrimonia Pilosa* Ledeb 20 g, *Curcuma aromatica* Salisb. 15 g, *Pinellia ternata* (Thunb.) Makino 12 g, *Citrus × aurantium* L. 10 g, *Scutellaria baicalensis* Georgi 10 g, *Platycodon grandiflorus* (Jacq.) A.DC. 5 g, Glycyrrhiza *glabra* L. 15 g	Decoction
Wu et al. (2012)	Lixu Jieyu prescription	Not reported	Decoction
Zhang et al. (2012a)	Lixu Jieyu prescription	*Astragalus mongholicus* Bunge, *Pueraria montana* var., *Codonopsis pilosula* (Franch.) Nannf., *Salvia miltiorrhiza* Bunge, *Rhodiola crenulata* (Hook.f. and Thomson) H.Ohba, *Panax notoginseng* (Burkill) F.H.Chen, *Epimedium brevicornu* Maxim., *Curcuma aromatica* Salisb., *Acorusgramineus* Aiton	Decoction
Zhang et al. (2012b)	Yaoyao Xiaopi prescription	*Polygala fallax* Hemsl 25 g, *Radix fici simplicissimae* 30 g, *Codonopsis pilosula* (Franch.) Nannf. 15 g, *Atractylodes macrocephala* Koidz. 10 g, *Salvia miltiorrhiza* Bunge 15 g, *Pueraria montana* var. 15 g, *Dimocarpus Longan* Lour. 15 g	Decoction
Zhao. (2012)	Buzhong Yiqi decoction and Xiaochaihu decoction	*Codonopsis pilosula* (Franch.) Nannf. 30 g, *Astragalus mongholicus* Bunge 30 g, *Atractylodes macrocephala* Koidz. 15 g, *Poria cocos* (Schw.) Wolf 15 g, *Bupleurum falcatum* L. 12 g, *Agrimonia Pilosa* Ledeb 20 g, *Curcuma aromatica* Salisb. 15 g, *Pinellia ternata* (Thunb.) Makino 12 g, *Citrus × aurantium* L. 10 g, *Scutellaria baicalensis* Georgi 10 g, *Platycodon grandiflorus* (Jacq.) A.DC. 5 g, Glycyrrhiza *glabra* L. 15 g	Decoction
Lai and Lei. (2013)	Baiyu Jianpi decoction	*Lilium Lancifolium *Thunb. 15 g, *Curcuma aromatica* Salisb. 10 g, *Bupleurum falcatum* L. 10 g, *Paeonia lactiflora* Pall. 10 g, *Astragalus mongholicus* Bunge 10 g, *Angelica sinensis* (Oliv.) Diels 10 g, *Cyperus rotundus* L. 10 g, *Citrus × aurantium* L. 6 g, *Atractylodes macrocephala* Koidz. 10 g, *Paeonia × Suffruticosa* Andrews 10 g, *Mentha Canadensis* L. 6 g	Decoction
Pang and Liu. (2013)	Shengmai pulvis and Xiaoyao pulvis	*Codonopsis pilosula* (Franch.) Nannf. 20 g, *Poria cocos* (Schw.) Wolf 15 g, *Atractylodes macrocephala* Koidz. 15 g, *Paeonia lactiflora *Pall. 15 g, *Angelica sinensis* (Oliv.) Diels 10 g, *Conioselinum anthriscoides* “Chuanxiong” 10 g, *Albiziae Cortex* 10 g, *Bupleurum falcatum* L. 10 g, *Astragalus mongholicus* Bunge 15 g, *Ophiopogon japonicus* (Thunb.) Ker Gawl. 10 g	Decoction

Study	Prescription name	Ingredients of herb prescription	Preparation
		*Schisandra chinensis* (Turcz.) Baill. 10 g, *Glycyrrhiza glabra* L. 6 g	
Xu et al. (2013)	Jiawei Naoxin kang	*Panax ginseng* C.A.Mey. 20 g, *Paeonia anomala subsp. veitchii* (Lynch) D.Y.Hong and K.Y.Pan 20 g, *Spatholobus suberectus* Dunn 20 g, *Polygonum multiflorum* Thunb. 20 g, *Conioselinum anthriscoides* “Chuanxiong” 20 g, *Angelica sinensis* (Oliv.) Diels 15 g, *Pheretima vulgaris* Chen 15 g, *Astragalus mongholicus* Bunge 30 g, *Lycopodium japonicum* Thunb. 25 g	Decoction
Xu and Wang. (2013)	Chaihu Jia Longgu Muli decoction	*Bupleurum falcatum* L. 15 g, *Scutellaria baicalensis* Georgi 12 g, *Pinellia ternata* (Thunb.) Makino 10 g, *Panax ginseng* C.A.Mey. 10 g, *Neolitsea cassia* (L.) Kosterm. 6 g, *Rheum Palmatum* L. 6 g, *Os Draconis* 30 g, *Ostrea gigas* Thunberg 30 g, *Succinum* 3 g, *Zingiber officinale* Roscoe 5 g, *Ziziphus Jujuba* Mill. 6 pieces	Decoction
Zhao. (2013)	Self-designed Baihe Yangxin Jianpi decoction	*Lilium Lancifolium* Thunb. 30 g, *Panax ginseng C.A.Mey*. 10 g, *Poria cocos* (Schw.) Wolf 20 g, *Astragalus mongholicus* Bunge 30 g, *Schisandra chinensis* (Turcz.) Baill. 12 g, *Dimocarpus Longan* Lour. 12 g, *Atractylodes macrocephala* Koidz. 15 g, *Ziziphi Spinosae Semen* 30 g, *Rehmannia glutinosa* (Gaertn.) DC. 30 g, *Cyperus rotundus* L. 12 g, *Eucommia ulmoides* Oliv. 30 g, *Albiziae Cortex* 12 g	Decoction
Zhao et al. (2013)	Compound of Fufangteng Mixture	*Euonymus fortunei* var. *fortunei*, *Astragalus mongholicus* Bunge, *Panax ginseng C.A.Mey*	Oral liquids
Teng et al. (2014)	Buzhong Jiepi decoction	*Codonopsis pilosula* (Franch.) Nannf. 20–30 g, *Astragalus mongholicus* Bunge 20–30 g, *Atractylodes macrocephala* Koidz. 15 g, *Glycyrrhiza glabra* L. 9 g, *Citrus × aurantium* L. 9 g, *Angelica sinensis* (Oliv.) Diels 9 g, *Actaea cimicifuga* L. 5 g, *Bupleurum falcatum* L. 5 g, *Pueraria montana* var*. lobata* (Willd.) Maesen and S.M.Almeida ex Sanjappa & Predeep. 15 g, Os Draconis 30 g, *Ostrea gigas* Thunberg 30 g	Decoction
Xu. (2014)	Qingshu Yiqi decoction	*Angelica sinensis* (Oliv.) Diels 1 g, *Astragalus mongholicus* Bunge 30 g, *Panax ginseng* C.A.Mey. 10 g, *Ophiopogon japonicus* (Thunb.) Ker Gawl. 10 g, *Schisandra chinensis* (Turcz.) Baill. 9 g, *Atractylodes lancea* (Thunb.) DC. 9 g, *Phellodendron amurense* Rupr 10 g, *Citrus × aurantium* L. 10 g, *Atractylodes macrocephala* Koidz. 12 g, *Alisma plantago-aquatica* subsp. 9 g, *Hyssopus officinalis* L. 12 g, *Pueraria montana* var*. lobata* (Willd.) Maesen and S.M.Almeida ex Sanjappa & Predeep. 9 g, *Actaea cimicifuga* L. 9 g, *Glycyrrhiza glabra* L. 6 g	Decoction
Gao and Pang. (2015)	Wendan decoction and Sini decoction	*Pinellia ternata* (Thunb.) Makino, *Bambusa tuldoides* Munro, *Glycyrrhiza glabra* L., *Bupleurum falcatum* L., *Citrus × aurantium* L., *Paeonia lactiflora *Pall., *Zingiber officinale* Roscoe, *Citrus × aurantium* L	Decoction
Li and Zao. (2015)	Jianpi Wenshen Shugan prescription	*Codonopsis pilosula* (Franch.) Nannf. 15 g, *Atractylodes macrocephala* Koidz. 15 g, *Poria cocos* (Schw.) Wolf 15 g, *Angelica sinensis* (Oliv.) Diels 15 g, *Conioselinum anthriscoides* “Chuanxiong” 15 g, *Paeonia lactiflora* Pall. 15 g, *Rehmannia glutinosa* (Gaertn.) DC. 15 g, *Curculigo Orchioides* Gaertn. 5 g, *Epimedium sagittatum* (Siebold & Zucc.) Maxim. 5 g, *Bupleurum falcatum* L. 10 g, *Coptis chinensis* Franch 10 g	Decoction
		*Gardenia Jasminoides* J.Ellis 10 g, *Curcuma aromatica* Salisb. 10 g, *Zingiber officinale* Roscoe 6 g, *Glycyrrhiza glabra* L. 6 g, Ziziphus Jujuba Mill. 6 pieces	
Liu et al. (2015)	Shugan Yangxue decoction	*Bupleurum falcatum* L. 10 g, *Rehmannia glutinosa* (Gaertn.) DC. 15 g, *Citrus medica* L. 10 g, *Curcuma aromatica* Salisb. 15 g, *Paeonia lactiflora* Pall. 10 g, *Conioselinum anthriscoides* ‘Chuanxiong’ 10 g, *Angelica sinensis* (Oliv.) Diels 10 g, *Albiziae Cortex* 20 g	Decoction
Li. (2015)	Buzhong Yiqi decoction and Xiaochaihu decoction	*Codonopsis pilosula* (Franch.) Nannf. 25g, *Astragalus mongholicus* Bunge 30 g, *Bupleurum falcatum* L. 15 g, *Agrimonia Pilosa* Ledeb 25g, *Atractylodes macrocephala* Koidz. 20 g, *Pinellia ternata* (Thunb.) Makino 15 g, P*oria cocos* (Schw.) Wolf 20 g, *Curcuma aromatica* Salisb. 20 g, *Platycodon grandiflorus* (Jacq.) A.DC. 6 g, *Citrus × aurantium* L. 15 g, *Scutellaria baicalensis* Georgi 12 g, *Glycyrrhiza glabra* L. 12 g, *Ziziphus Jujuba* Mill. 12 g	Decoction
Niu et al. (2015)	Bushen Shugan decoction	*Rehmannia glutinosa* (Gaertn.) DC. 20 g, *Lycium chinense* Mill. 15 g, *Rehmannia glutinosa* (Gaertn.) DC. 20 g, *Scrophularia ningpoensis* Hemsl 15 g, *Ophiopogon japonicus* (Thunb.) Ker Gawl. 15 g, *Angelica sinensis* (Oliv.) Diels 12 g, *Conioselinum anthriscoides* “Chuanxiong” 10 g, B*upleurum falcatum* L. 10 g, *Citrus × aurantium* L. 10 g, *Scutellaria baicalensis* Georgi 12 g, *Coptis chinensis* Franch 6 g, *Glycyrrhiza glabra* L. 6 g	Decoction
Tan et al. (2015)	Sini Decoction and Wulin powder	*Cyperus rotundus* L. 9 g, *Zingiber officinale* Roscoe 9 g, *Glycyrrhiza glabra* L. 9 g, *Neolitsea cassia* (L.) Kosterm. 10 g, *Poria cocos* (Schw.) Wolf 15 g, Polyporus umbellatus (Pers)Fr. 15 g, *Atractylodes macrocephala* Koidz. 10 g, *Alisma plantago-aquatica* subsp. 15 g, *Plantago Asiatica* L. 15 g, *Bupleurum falcatum* L. 9 g, *Asarum Heterotropoides* F.Schmidt 3 g, *Brassica Juncea* (L.) Czern. 6 g	Decoction

Study	Prescription name	Ingredients of herb prescription	Preparation
Zhang et al. (2015)	Wenzhen Yunqi prescription	*Astragalus mongholicus* Bunge, *Pueraria montana* var*. lobata* (Willd.) Maesen and S.M.Almeida ex Sanjappa & Predeep., *Curcuma aromatica* Salisb., *Acorus Gramineus* Aiton, *Actinolitum*	Decoction
Gao and Pang. (2016)	Wendan decoction and Sini powder	*Pinellia ternata* (Thunb.) Makino 6 g, *Bambusa tuldoides* Munro 6 g, *Glycyrrhiza glabra* L. 6 g, *Bupleurum falcatum* L. 6 g, *Citrus × aurantium* L. 6 g, *Paeonia lactiflora *Pall. 6 g, *Zingiber officinale* Roscoe 12 g, *Citrus × aurantium* L. 9 g	Decoction
Shi and Wu. (2016)	Suanzaoren decoction	*Ziziphi Spinosae Semen* 15 g, *Glycyrrhiza glabra* L. 3 g, *Anemarrhena asphodeloides* Bunge 6 g, *Poria cocos* (Schw.) Wolf 6 g, *Conioselinum anthriscoides* “Chuanxiong” 6 g	Decoction
Sun et al. (2016)	Shugan Yiyang capsule	*Bupleurum falcatum* L., *Tribulus Terrestris* L., *Aspongopus chinensis* Dallas, *Polistes mandarinus* Saussure, *Cnidium Monnieri* (L.) Cusson, *Cistanche Deserticola* Ma, *Cuscuta chinensis* Lam, *Schisandra chinensis* (Turcz.) Baill., *Gynochthodes Officinalis* (F.C.How) Razafim. and B.Bremer, *Polygala tenuifolia* Willd., Acorus Gramineus Aiton, *Pheretima vulgaris* Chen, *Whitmania pigra* Whitman, *Scolopendra subspinipes mutilans* L. Koch	Capsule
Wu et al. (2016)	Xiaopi Yin	*Panax ginseng* C.A.Mey. 20 g, *Atractylodes macrocephala* Koidz. 15 g, *Poria cocos* (Schw.) Wolf 15 g, *Cuscuta chinensis* Lam. 10 g, *Lycium chinense* Mill. 10 g, *Epimedium brevicornu* Maxim. 10 g, *Dioscorea oppositifolia* L. 20 g, *Psoralea corylifolia* L. 10 g, *Angelica sinensis* (Oliv.) Diels 10 g, *Alisma plantago-aquatica* subsp. 6 g, *Astragalus mongholicus* Bunge 20 g, *Matricaria Chamomilla* L. 10 g, *Glycyrrhiza glabra* L. 6 g	Decoction
Wang. (2017)	Bupi Yishen decoction	*Panax ginseng* C.A.Mey. 10 g, *Atractylodes macrocephala* Koidz. 30 g, *Rhodiola Crenulata* (Hook.F. and Thomson) H.Ohba 15 g, *Poria cocos* (Schw.) Wolf 12 g, *Cuscuta chinensis* Lam. 15 g, *Psoralea corylifolia* L. 20 g, *Citrus × aurantium* L. 12 g, *Cornus Officinalis* Siebold & Zucc. 15 g, *Dioscorea oppositifolia* L. 15 g, *Pinellia ternata* (Thunb.) Makino 10 g, *Glycyrrhiza glabra* L. 10 g, *Bupleurum falcatum* L. 10 g	Decoction
Ye. (2017)	Shenxian congee	*Dioscorea oppositifolia* L. 10 g, *Euryale ferox* Salisb. 10 g, *Allium tuberosum* Rottler ex Spreng. 10 g, *Zea mays* L. 50 g	Herbal porridge
Zheng et al. (2017)	Shugan Jianpi Yishen prescription	*Astragalus mongholicus* Bunge 30 g, *Codonopsis pilosula* (Franch.) Nannf. 12 g, *Atractylodes macrocephala* Koidz. 12 g, *Anemarrhena asphodeloides* Bunge 10 g, *Citrus × aurantium* L. 10 g, *Bupleurum falcatum* L. 10 g, *Actaea cimicifuga* L. 10 g, *Paeonia lactiflora* Pall. 10 g, *Cuscuta chinensis* Lam. 10 g, *Epimedium sagittatum* (Siebold & Zucc.) Maxim. 10 g, *Lycium chinense* Mill. 10 g, *Glycyrrhiza glabra* L. 6 g	Decoction
Du (2018)	Self-designed Yishen Buxue ointment	*Angelica sinensis* (Oliv.) Diels 10 g, *Rehmannia glutinosa* (Gaertn.) DC. 15 g, *Paeonia lactiflora* Pall. 10 g, *Conioselinum anthriscoides* “Chuanxiong” 10 g, *Cuscuta chinensis* Lam. 15 g, *Epimedium sagittatum* (Siebold & Zucc.) Maxim.12 g, *Psoralea corylifolia* L. 10 g, *Lycium chinense *Mill. 10 g	Decoction
Li et al. (2018)	Yiqi Yangxue Bupi Hegan prescription	*Astragalus mongholicus* Bunge 40 g, *Atractylodes macrocephala* Koidz. 15 g, *Paeonia lactiflora* Pall. 15 g, *Poria cocos* (Schw.) Wolf 15 g, *Dioscorea oppositifolia* L. 15 g, *Panax ginseng* C.A.Mey. 10 g, *Rehmannia glutinosa* (Gaertn.) DC. 10 g, *Angelica sinensis* (Oliv.) Diels 10 g, *Conioselinum anthriscoides* “Chuanxiong” 10 g, *Bupleurum falcatum* L. 10 g, *Cyperus rotundus* L. 10 g, *Corydalis yanhusuo* (Y.H.Chou & Chun C.Hsu) W.T.Wang ex Z.Y.Su and C.Y.Wu 10 g, *Poria cocos* (Schw.) Wolf 10 g, *Gardenia Jasminoides* J.Ellis 10 g, *Glycyrrhiza glabra* L. 10 g	Decoction
Liu and Cai. (2018)	Bupiwei Xieyinhuo Shengyang decoction	*Bupleurum falcatum* L. 15 g, *Astragalus mongholicus* Bunge 10 g, *Atractylodes lancea* (Thunb.) DC. 10 g, *Hansenia Weberbaueriana* (Fedde Ex H.Wolff) Pimenov & Kljuykov 10 g, *Glycyrrhiza glabra* L. 10 g, *Actaea cimicifuga* L. 8 g, *Panax ginseng* C.A.Mey. 7 g, *Scutellaria baicalensis* Georgi 7 g, *Coptis chinensis* Franch 5 g	Decoction
Ou et al. (2018)	Guipi decoction	*Astragalus mongholicus* Bunge 30 g, *Ziziphi Spinosae Semen* 25 g, *Codonopsis pilosula* (Franch.) Nannf. 15 g, *Poria cocos* (Schw.) Wolf 15 g, *Dimocarpus Longan* Lour. 15 g, *Atractylodes macrocephala* Koidz. 15 g, *Polygala tenuifolia *Willd. 15 g, *Angelica sinensis* (Oliv.) Diels 15 g, *Glycyrrhiza glabra* L. 10 g, *Dolomiaea Costus* (Falc.) Kasana and A.K.Pandey 7 g	Decoction
Weng (2018)	Liujunzi decoction	A*tractylodes macrocephala* Koidz. 9 g, *Panax ginseng* C.A.Mey. 9 g, *Citrus × aurantium* L. 3 g, *Glycyrrhiza glabra* L. 6 g, *Poria cocos* (Schw.) Wolf 9 g, *Pinellia ternata* (Thunb.) Makino 4.5 g	Decoction

Study	Prescription name	Ingredients of herb prescription	Preparation
Wu et al. (2018)	Guipi decoction	*Codonopsis pilosula* (Franch.) Nannf. 20 g, *Astragalus mongholicus* Bunge 20 g, *Dimocarpus Longan* Lour. 15 g, *Ziziphi Spinosae Semen* 15 g, *Poria cocos* (Schw.) Wolf 15 g, *Atractylodes macrocephala* Koidz. 10 g, *Angelica sinensis* (Oliv.) Diels 10 g, *Conioselinum anthriscoides* “Chuanxiong” 10 g, *Paeonia lactiflora* Pall. 10 g, *Bupleurum falcatum* L. 10 g, *Citrus × aurantium* L. 10 g, *Curcuma aromatica* Salisb. 10 g, *Dolomiaea Costus* (Falc.) Kasana and A.K.Pandey 10 g, *Polygala tenuifolia* Willd. 10 g, *Citrus × aurantium* L. 10 g, *Actaea cimicifuga* L. 10 g, *Glycyrrhiza glabra* L. 6 g	Decoction
Luo (2018)	Qingre Qushi prescription	*Panax Quinquefolius *L. 10 g, *Morus alba* L. 10 g, *Prunus armeniaca* L. 10 g, *Pogostemon cablin* (Blanco) Benth. 10 g, *Atractylodes lancea* (Thunb.) DC. 10 g, *Magnolia officinalis* var. biloba Rehder and E.H.Wilson 10 g, *Citrus × aurantium* L. 10 g, *Artemisia Capillaris *Thunb. 10 g, *Coix lacryma-jobi* L. 50 g, *Coptis chinensis* Franch 5 g, *Zingiber officinale* Roscoe 5 g, *Saposhnikovia divaricata* (Turcz. ex Ledeb.) Schischk. 10 g, *Tetrapanax papyrifer* (Hook.) K.Koch 10 g, *Glycyrrhiza glabra* L. 5 g	Decoction
Ding (2019)	Guipi decoction	*Astragalus mongholicus* Bunge 30 g, *Ziziphi Spinosae Semen* 25 g, *Poria cocos* (Schw.) Wolf 15 g, *Dimocarpus Longan* Lour. 15 g, *Polygala tenuifolia* Willd. 15 g, *Angelica sinensis* (Oliv.) Diels 15 g, *Atractylodes macrocephala* Koidz. 15 g, *Codonopsis pilosula* (Franch.) Nannf. 15 g, *Dolomiaea Costus* (Falc.) Kasana and A.K.Pandey 7 g, *Glycyrrhiza glabra* L. 10 g	Decoction
He (2019)	Shugan Jianpi Huoxue prescription	*Bupleurum falcatum* L. 15 g, *Cyperus rotundus* L. 15 g, *Codonopsis pilosula* (Franch.) Nannf. 12 g, *Atractylodes macrocephala* Koidz. 12 g, *Poria cocos* (Schw.) Wolf 9 g, *Eleutherococcus Senticosus* (Rupr. and Maxim.) Maxim. 12 g, *Agrimonia Pilosa* Ledeb 20 g, *Angelica sinensis* (Oliv.) Diels 15 g, *Conioselinum anthriscoides* “Chuanxiong” 10 g, *Salvia miltiorrhiza* Bunge 10 g, *Glycyrrhiza glabra* L. 6 g	Decoction
Hu (2019)	Buzhong Yiqi and Xiaochaihu decoction	*Codonopsis pilosula* (Franch.) Nannf. 24 g, *Astragalus mongholicus* Bunge 29 g, *Bupleurum falcatum* L. 14 g, *Agrimonia Pilosa* Ledeb 24 g, *Atractylodes macrocephala* Koidz. 21 g, *Pinellia ternata* (Thunb.) Makino 14 g, *Poria cocos* (Schw.) Wolf 21 g, *Curcuma aromatica* Salisb. 21 g, *Platycodon grandiflorus* (Jacq.) A.DC. 7 g, *Citrus × aurantium* L. 14 g, *Scutellaria baicalensis* Georgi 14 g	Decoction
Liu et al. (2019a)	Jiawei Lingzhi pill	*Polygonum multiflorum* Thunb. 30 g, *Colla Carapacis et Plastri Testudinis* 12 g, *Ganoderma lucidum* (Leyss. ex Fr.) Karst. 10 g, *Astragalus mongholicus* Bunge 30 g, *Panax notoginseng* (Burkill) F.H.Chen 5 g, *Acorus Gramineus* Aiton 5 g, *Polygala tenuifolia* Willd. 10 g	Pill
Liu et al. (2019b)	Chaihu Guizhi decoction grain	*Bupleurum falcatum* L., *Neolitsea cassia* (L.) Kosterm., *Codonopsis pilosula* (Franch.) Nannf., *Scutellaria baicalensis* Georgi, *Pinellia ternata* (Thunb.) Makino, *Paeonia lactiflora* Pall., *Glycyrrhiza glabra* L., *Zingiber officinale* Roscoe, *Ziziphus Jujuba* Mill	Granule
Liu et al. (2019c)	Jianpi Yishen decoction	*Cuscuta chinensis* Lam. 10 g, *Lycium chinense* Mill. 15 g, *Pseudostellaria Heterophylla* (Miq.) Pax 15 g, *Atractylodes macrocephala* Koidz. 10 g, *Poria cocos* (Schw.) Wolf 10 g, *Dioscorea oppositifolia* L. 20 g, *Citrus × aurantium* L. 10 g, *Cistanche Deserticola* Ma 10 g, *Psoralea corylifolia* L. 10 g, *Poria cocos* (Schw.) Wolf 12 g, *Polygala tenuifolia* Willd. 12 g, *Glycyrrhiza glabra* L.6 g	Decoction
Ma et al. (2019)	Self-designed Jiawei Erxian decoction	*Epimedium brevicornu* Maxim. 15 g, *Curculigo Orchioides* Gaertn. 10 g, *Gynochthodes Officinalis* (F.C.How) Razafim. and B.Bremer 10 g, *Astragalus mongholicus* Bunge 30 g, *Codonopsis pilosula* (Franch.) Nannf. 15 g, *Angelica sinensis* (Oliv.) Diels 10 g, *Actaea cimicifuga* L. *Actaea heracleifolia* (Kom.) J.Compton 6 g, *Bupleurum falcatum* L. 6 g, *Phellodendron amurense* Rupr 5 g, *Anemarrhena asphodeloides* Bunge 10 g	Decoction
Shi (2019)	Xiaoyao pulvis Jiawei	*Angelica sinensis* (Oliv.) Diels 10 g, *Paeonia lactiflora* Pall. 10 g, *Bupleurum falcatum* L. 6 g, *Poria cocos* (Schw.) Wolf 20 g, *Atractylodes macrocephala* Koidz. 10 g, *Glycyrrhiza glabra* L. 6 g, *Mentha Canadensis* L. 6 g, *Codonopsis pilosula* (Franch.) Nannf. 15 g, *Dioscorea oppositifolia* L. 20 g	Decoction
Wang (2019)	Sanren decoction and Sijunzi decoction	*Prunus armeniaca* L. 10 g, Wurfbainia Vera (Blackw.) Skornick. and A.D.Poulsen 10 g, *Coix lacryma-jobi* L. 50 g, *Pinellia ternata* (Thunb.) Makino 10 g, *Magnolia officinalis* var. *biloba* Rehder and E.H.Wilson 10 g, *Panax ginseng* C.A.Mey. 10 g, *Atractylodes macrocephala* Koidz. 10 g, *Poria cocos* (Schw.) Wolf 20 g, *Tetrapanax papyrifer* (Hook.) K.Koch 10 g, *Glycyrrhiza glabra* L. 5 g	Decoction
Yang (2019)	Zuogui pill	*Rehmannia glutinosa* (Gaertn.) DC. 250 g, *Dioscorea oppositifolia* L. 120 g, *Cornus Officinalis* Siebold & Zucc. 90 g, *Lycium chinense* Mill. 20 g, *Cervus nippon* Temminck 120 g, *Cuscuta chinensis* Lam. 120 g, *Eucommia ulmoides* Oliv. 120 g, *Angelica sinensis* (Oliv.) Diels 90 g, *Neolitsea cassia* (L.) Kosterm. 60 g, *Cyperus Rotundus* L. 60 g	Pill
Dong (2020)	Qingshu Yiqi decoction grain	*Glycyrrhiza glabra* L. 6 g, Actaea cimicifuga L. 9 g, *Schisandra chinensis* (Turcz.) Baill. 9 g, *Amomum villosum *Lour. 10 g, *Citrus × aurantium* L. 10 g, *Phellodendron amurense* Rupr 12 g, *Citrus × aurantium* L. 12 g, *Atractylodes lancea* (Thunb.) DC. 15 g, *Poria cocos* (Schw.) Wolf 15 g, *Alisma plantago-aquatica* subsp. 15 g, *Angelica sinensis* (Oliv.) Diels 15 g, *Codonopsis pilosula* (Franch.) Nannf. 15 g, *Atractylodes macrocephala* Koidz. 15 g, *Mosla Chinensis* Maxim. 15 g, *Hyssopus officinalis* L. 20 g, *Astragalus mongholicus* Bunge 30 g	Decoction
Li (2020)	Buzhong Yiqi decoction and Xiaochaihu decoction	*Bupleurum falcatum* L. 15 g, *Atractylodes macrocephala* Koidz. 15 g, *Poria cocos* (Schw.) Wolf 15 g, *Codonopsis pilosula* (Franch.) Nannf. 15 g, *Curcuma aromatica* Salisb. 10 g, *Glycyrrhiza glabra* L. 12 g, *Ziziphus Jujuba* Mill. 5 pieces	Decoction
Mao (2020)	Yishen Tiaodu method	*Dioscorea oppositifolia* L. 10 g, *Zea mays* L. 50 g, *Euryale Ferox Salisb*. 10 g, *Allium tuberosum* Rottler ex Spreng. 10 g	Decoction
Wang (2020)	Chaihu Guizhi decoction	*Bupleurum falcatum* L. 12 g, *Neolitsea cassia* (L.) Kosterm. 9 g, *Scutellaria baicalensis* Georgi 9 g, *Paeonia lactiflora* Pall. 9 g, *Codonopsis pilosula* (Franch.) Nannf. 9 g, *Pinellia ternata* (Thunb.) Makino 9 g, *Glycyrrhiza glabra* L. 9 g, *Ziziphus Jujuba* Mill. 6 pieces, *Zingiber officinale* Roscoe 6 g	Decoction
Chen (2021)	Xiaoyao powder	*Poria cocos* (Schw.) Wolf 12 g, *Paeonia lactiflora* Pall. 12 g, *Atractylodes macrocephala* Koidz. 12 g, *Angelica sinensis* (Oliv.) Diels 10 g, *Glycyrrhiza glabra* L. 6 g, *Bupleurum falcatum* L. 6 g, *Mentha Canadensis* L. 5 g, *Zingiber officinale* Roscoe 3 g	Decoction
Li et al. (2021)	Jiannao Yizhi ointment	*Astragalus mongholicus* Bunge 30 g, *Ziziphi Spinosae Semen* 25 g, *Poria cocos* (Schw.) Wolf 15 g, *Dimocarpus Longan* Lour. 15 g, *Polygala tenuifolia* Willd. 15 g, *Angelica sinensis* (Oliv.) Diels 15 g, *Atractylodes macrocephala* Koidz. 15 g, *Codonopsis pilosula* (Franch.) Nannf. 15 g, *Dolomiaea Costus* (Falc.) Kasana and A.K.Pandey 7 g, *Glycyrrhiza glabra* L. 10 g	Ointment
Liu et al. (2021)	Chaihu Guizhi decoction grain	*Bupleurum falcatum* L. 15 g, *Cyperus rotundus* L. 15 g, *Codonopsis pilosula* (Franch.) Nannf. 12 g, *Atractylodes macrocephala* Koidz. 12 g, *Poria cocos* (Schw.) Wolf 9 g, *Eleutherococcus Senticosus* (Rupr. and Maxim.) Maxim. 12 g, *Agrimonia Pilosa* Ledeb 20 g, *Angelica sinensis* (Oliv.) Diels 15 g, *Conioselinum anthriscoides* “Chuanxiong” 10 g, *Salvia miltiorrhiza* Bunge 10 g, *Glycyrrhiza glabra* L. 6 g	Decoction
Wang (2021)	Shenling Baizhu powder	*Codonopsis pilosula* (Franch.) Nannf. 24 g, *Astragalus mongholicus* Bunge 29 g, *Bupleurum falcatum* L. 14 g, *Agrimonia Pilosa* Ledeb 24 g, *Atractylodes macrocephala* Koidz. 21 g, *Pinellia ternata* (Thunb.) Makino 14 g, *Poria cocos* (Schw.) Wolf 21 g, *Curcuma aromatica* Salisb. 21 g, *Platycodon grandiflorus* (Jacq.) A.DC. 7 g, *Citrus × aurantium* L. 14 g, *Scutellaria baicalensis* Georgi 14 g	Decoction
Zhang (2021)	Jiawei Guizhi Xinjia decoction	*Polygonum multiflorum* Thunb. 30 g, *Colla Carapacis et Plastri Testudinis* 12 g, *Ganoderma lucidum* (Leyss. ex Fr.) Karst. 10 g, *Astragalus mongholicus* Bunge 30 g, *Panax notoginseng* (Burkill) F.H.Chen 5 g, *Acorus Gramineus* Aiton 5 g, *Polygala tenuifolia* Willd. 10 g	Decoction
Sheng et al. (2022)	Wenshen Lipi prescription	*Bupleurum falcatum* L., *Neolitsea cassia* (L.) Kosterm., *Codonopsis pilosula* (Franch.) Nannf., *Scutellaria baicalensis* Georgi, *Pinellia ternata* (Thunb.) Makino, *Paeonia lactiflora* Pall., *Glycyrrhiza glabra* L., *Zingiber officinale* Roscoe, *Ziziphus Jujuba* Mill.	Granule

The quality assessment of the included studies is listed in Table 3. The Cochrane score ranged from 3 to 7, and three studies (Liu J. et al., 2019; Liu et al., 2021; Sheng et al., 2022) got 7 points; two studies (Li, 2009; Ye, 2017) got 6 points; two studies (Luo, 2018; Wang, 2019) got 5 points; 38 studies (Fang et al., 2007; Wang et al., 2007; Zhang et al., 2009; Li et al., 2011; Liu et al., 2011; Zhang et al., 2011; Jiang, 2012; Ren and Yu, 2012; Wang, 2012; Zhao, 2012; Lai and Lei, 2013; Pang and Liu, 2013; Xu and Wang, 2013; Zhao et al., 2013; Xu, 2014; Gao and Pang, 2015; Liu et al., 2015; Zhang et al., 2015; Gao and Pang, 2016; Sun et al., 2016; Wu et al., 2016; Wang, 2017; Zheng et al., 2017; Du, 2018; Liu and Cai, 2018; Ou et al., 2018; Weng, 2018; Liu Y. et al., 2019; Ding, 2019; He, 2019; Hu, 2019; Yang, 2019; Dong, 2020; Li, 2020; Mao, 2020; Li et al., 2021; Wang, 2021; Zhang, 2021) got 4 points; and 39 studies (Ning and Li, 2002; Yang et al., 2004; Zhang et al., 2004; Zhang and Zhou, 2004; Wei, 2005; Yao and Qiu, 2005; Liang, 2006; Zhao et al., 2006; Gong, 2007; Guo et al., 2007; Lin, 2007; Sun et al., 2007; Fang et al., 2008; Cheng, 2009; Jie and Wang, 2009; Ma, 2009; Hu et al., 2010; Chen et al., 2011; Wang et al., 2011; Kong, 2012; Tian and Wang, 2012; Wu et al., 2012; Zhang Z. X. et al., 2012; Zhang L. P. et al., 2012; Xu et al., 2013; Zhao, 2013; Teng et al., 2014; Li and Zao, 2015; Li, 2015; Niu et al., 2015; Tan et al., 2015; Shi and Wu, 2016; Li et al., 2018; Wu et al., 2018; Liu F. et al., 2019; Ma et al., 2019; Shi, 2019; Wang, 2020; Chen, 2021) got 3 points. All of the included studies reported random allocation, and 44 studies (Fang et al., 2007; Wang et al., 2007; Li, 2009; Zhang et al., 2009; Li et al., 2011; Liu et al., 2011; Zhang et al., 2011; Jiang, 2012; Ren and Yu, 2012; Wang, 2012; Zhao, 2012; Lai and Lei, 2013; Pang and Liu, 2013; Xu and Wang, 2013; Zhao et al., 2013; Xu, 2014; Gao and Pang, 2015; Liu et al., 2015; Zhang et al., 2015; Gao and Pang, 2016; Sun et al., 2016; Wu et al., 2016; Wang, 2017; Ye, 2017; Zheng et al., 2017; Du, 2018; Liu and Cai, 2018; Luo, 2018; Ou et al., 2018; Weng, 2018; Liu J. et al., 2019; Ding, 2019; He, 2019; Hu, 2019; Wang, 2019; Yang, 2019; Dong, 2020; Li, 2020; Mao, 2020; Li et al., 2021; Liu et al., 2021; Wang, 2021; Zhang, 2021; Sheng et al., 2022) described the method of random sequence generation, whereas the remaining 40 studies (Ning and Li, 2002; Yang et al., 2004; Zhang et al., 2004; Zhang and Zhou, 2004; Wei, 2005; Yao and Qiu, 2005; Liang, 2006; Zhao et al., 2006; Gong, 2007; Guo et al., 2007; Lin, 2007; Sun et al., 2007; Fang et al., 2008; Cheng, 2009; Jie and Wang, 2009; Ma, 2009; Hu et al., 2010; Chen et al., 2011; Wang et al., 2011; Kong, 2012; Tian and Wang, 2012; Wu et al., 2012; Zhang Z. X. et al., 2012; Zhang L. P. et al., 2012; Xu et al., 2013; Zhao, 2013; Teng et al., 2014; Li and Zao, 2015; Li, 2015; Niu et al., 2015; Tan et al., 2015; Shi and Wu, 2016; Li et al., 2018; Wu et al., 2018; Liu F. et al., 2019; Liu Y. et al., 2019; Ma et al., 2019; Shi, 2019; Wang, 2020; Chen, 2021) provided no details. Five studies (Li, 2009; Ye, 2017; Liu J. et al., 2019; Liu et al., 2021; Sheng et al., 2022) mentioned concealment allocation. Three trials (Liu J. et al., 2019; Liu et al., 2021; Sheng et al., 2022) reported double blinding of patients and physicians, and eight trials (Li, 2009; Ye, 2017; Luo, 2018; Liu J. et al., 2019; Liu Y. et al., 2019; Wang, 2019; Liu et al., 2021; Sheng et al., 2022) described blinding of participants. All studies met the criterion of incomplete outcome data as drop-out data, or no drop-out patients were reported specifically. Pre-designed outcomes were reported in all studies, detecting a low risk of reporting bias, and other biases were not found in all included studies.

**TABLE 3:** Risk of bias assessment of all included studies.

Study	Seven-item criteria							
	A	B	C	D	E	F	G	Total
Ning and Li. (2002)	?	?	?	?	?	?	?	3?
Yang et al. (2004)	?	?	?	?	?	?	?	3?
Zhang et al. (2004)	?	?	?	?	?	?	?	3?
Zhang and Zhou. (2004)	?	?	?	?	?	?	?	3?
Wei (2005)	?	?	?	?	?	?	?	3?
Yao and Qiu. (2005)	?	?	?	?	?	?	?	3?
Liang (2006)	?	?	?	?	?	?	?	3?
Zhao et al. (2006)	−	?	?	?	?	?	?	3?
Fang et al. (2007)	?	?	?	?	?	?	?	4?
Gong. (2007)	?	?	?	?	?	?	?	3?
Guo et al. (2007)	?	?	?	?	?	?	?	3?
Lin (2007)	?	?	?	?	?	?	?	3?
Sun et al. (2007)	?	?	?	?	?	?	?	3?
Wang et al. (2007)	?	?	?	?	?	?	?	4?
Fang et al. (2008)	?	?	?	?	?	?	?	3?
Cheng (2009)	?	?	?	?	?	?	?	3?
Jie and Wang (2009)	?	?	?	?	?	?	?	3?
Li (2009)	?	?	?	?	?	?	?	6?
Ma (2009)	?	?	?	?	?	?	?	3?
Zhang et al. (2009)	?	?	?	?	?	?	?	4?
Hu et al. (2010)	?	?	?	?	?	?	?	3?
Chen et al. (2011)	−	?	?	?	?	?	?	3?
Li et al. (2011)	?	?	?	?	?	?	?	4?
Liu et al. (2011)	?	?	?	?	?	?	?	4?
Wang et al. (2011)	−	?	?	?	?	?	?	3?
Zhang et al. (2011)	?	?	?	?	?	?	?	4?
Jiang (2012)	?	?	?	?	?	?	?	4?
Kong (2012)	?	?	?	?	?	?	?	3?
Ren and Yu (2012)	?	?	?	?	?	?	?	4?
Tian and Wang (2012)	?	?	?	?	?	?	?	3?
Wang (2012)	?	?	?	?	?	?	?	4?
Wu et al. (2012)	?	?	?	?	?	?	?	3?
Zhang et al. (2012a)	?	?	?	?	?	?	?	3?
Zhang et al. (2012b)	?	?	?	?	?	?	?	3?
Zhao (2012)	?	?	?	?	?	?	?	4?
Lai and Lei (2013)	?	?	?	?	?	?	?	4?
Pang and Liu (2013)	?	?	?	?	?	?	?	4?
Xu et al. (2013)	?	?	?	?	?	?	?	3?
Xu and Wang (2013)	?	?	?	?	?	?	?	4?
Zhao (2013)	?	?	?	?	?	?	?	3?
Zhao et al. (2013)	?	?	?	?	?	?	?	4?
Teng et al. (2014)	?	?	?	?	?	?	?	3?
Xu (2014)	?	?	?	?	?	?	?	4?
Gao and Pang (2015)	?	?	?	?	?	?	?	4?
Li and Zao (2015)	?	?	?	?	?	?	?	3?
Liu et al. (2015)	?	?	?	?	?	?	?	4?
Li (2015)	?	?	?	?	?	?	?	3?
Niu et al. (2015)	?	?	?	?	?	?	?	3?
Tan et al. (2015)	−	?	?	?	?	?	?	3?
Zhang et al. (2015)	?	?	?	?	?	?	?	4?
Gao and Pang (2016)	?	?	?	?	?	?	?	4?
Shi and Wu (2016)	?	?	?	?	?	?	?	3?
Sun et al. (2016)	?	?	?	?	?	?	?	4?
Wu et al. (2016)	?	?	?	?	?	?	?	4?
Wang (2017)	?	?	?	?	?	?	?	4?
Ye (2017)	?	?	?	?	?	?	?	6?
Zheng et al. (2017)	?	?	?	?	?	?	?	4?
Du (2018)	?	?	?	?	?	?	?	4?
Li et al. (2018)	?	?	?	?	?	?	?	3?
Liu and Cai (2018)	?	?	?	?	?	?	?	4?
Ou et al. (2018)	?	?	?	?	?	?	?	4?
Weng (2018)	?	?	?	?	?	?	?	4?
Wu et al. (2018)	?	?	?	?	?	?	?	3?
Luo (2018)	?	?	?	?	?	?	?	5?
Ding (2019)	?	?	?	?	?	?	?	4?
He (2019)	?	?	?	?	?	?	?	4?
Hu (2019)	?	?	?	?	?	?	?	4?
Liu et al. (2019a)	?	?	?	?	?	?	?	3?
Liu et al. (2019b)	?	?	?	?	?	?	?	7?
Liu et al. (2019c)	?	?	?	?	?	?	?	4?
Ma et al. (2019)	?	?	?	?	?	?	?	3?
Shi (2019)	?	?	?	?	?	?	?	3?
Wang (2019)	?	?	?	?	?	?	?	5?
Yang (2019)	?	?	?	?	?	?	?	4?
Dong (2020)	?	?	?	?	?	?	?	4?
Li (2020)	?	?	?	?	?	?	?	4?
Mao (2020)	?	?	?	?	?	?	?	4?
Wang (2020)	?	?	?	?	?	?	?	3?
Chen (2021)	−	?	?	?	?	?	?	3?
Li et al. (2021)	?	?	?	?	?	?	?	4?
Liu et al. (2021)	?	?	?	?	?	?	?	7?
Wang (2021)	?	?	?	?	?	?	?	4?
Zhang (2021)	?	?	?	?	?	?	?	4?
Sheng et al. (2022)	?	?	?	?	?	?	?	7?

## Results of meta-analysis

### Primary outcomes

#### FS-14 scores

**FIGURE 2:**
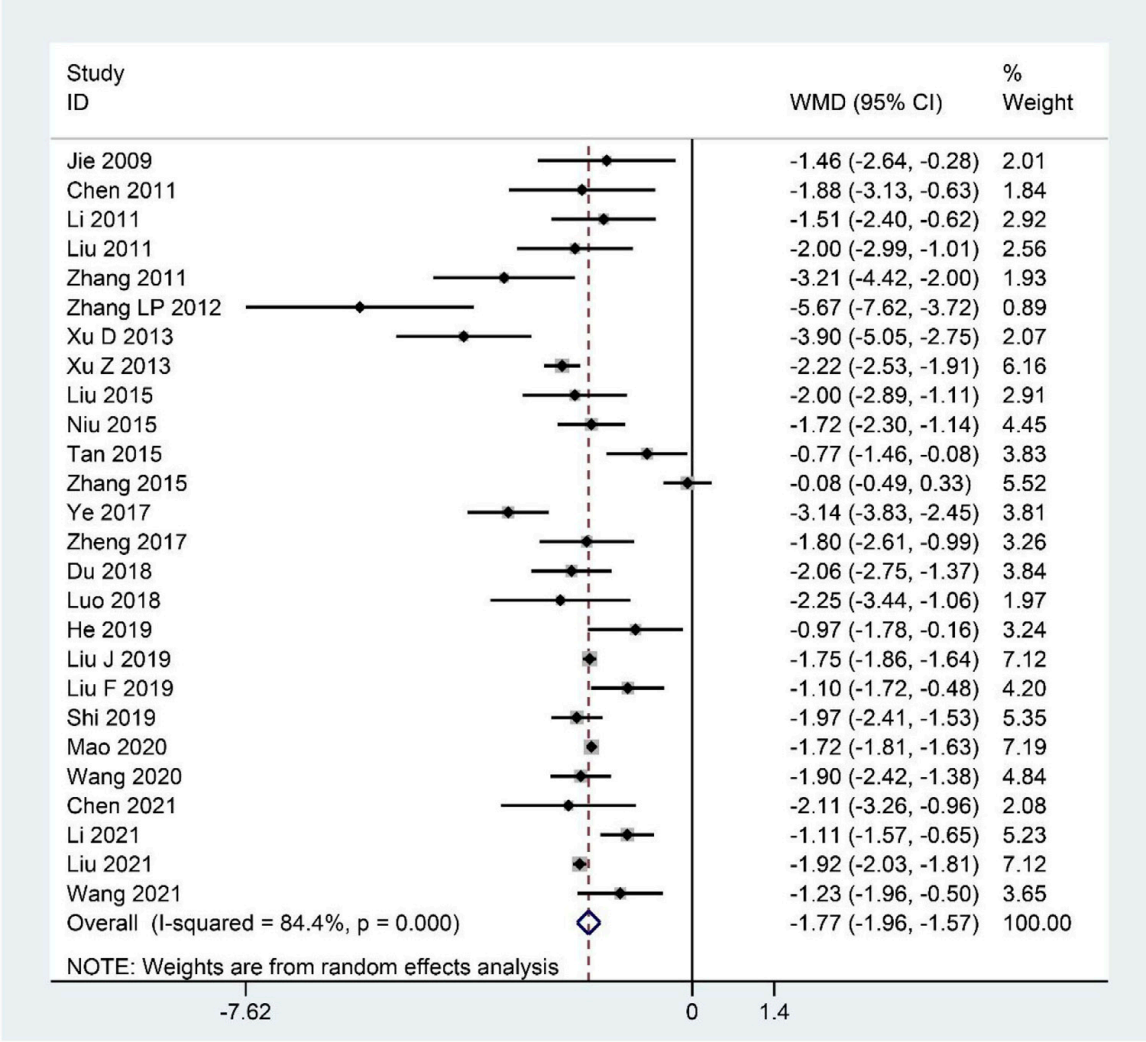
Forest plot for FS-14 scores.

Pooled data from the 26 studies (Jie and Wang, 2009; Chen et al., 2011; Li et al., 2011; Liu et al., 2011; Zhang et al., 2011; Zhang L. P. et al., 2012; Xu et al., 2013; Xu and Wang, 2013; Liu et al., 2015; Niu et al., 2015; Tan et al., 2015; Zhang et al., 2015; Ye, 2017; Zheng et al., 2017; Du, 2018; Luo, 2018; Liu F. et al., 2019; Liu J. et al., 2019; He, 2019; Shi, 2019; Mao, 2020; Wang, 2020; Chen, 2021; Li et al., 2021; Liu et al., 2021; Wang, 2021) reporting the FS-14 scores showed that CHM clearly decreased the FS-14 scores as an adjuvant or monotherapy for CFS compared with the contrast group (WMD: –1.77; 95%CI: –1.96 to –1.57; *p* < 0.001; *p* for heterogeneity <0.001; I^2^ = 84.4%; Figure 2). The subgroup analysis showed similar results (Table 4).

**TABLE 4:** Subgroup analysis for outcomes.

Subgroup		No. of studies	Effect size (95% CI)	*p*-value	Heterogeneity	*p*-value
Subgroup analyses for FS-14 scores						
CHM vs. WCM		17	WMD −1.84 (−2.23, −1.45)	<0.001	85.9	<0.001
	Duration of intervention ≤ 30 days	5	WMD −1.90 (−2.75, −1.05)	<0.001	85.0	<0.001
	30 days < duration of intervention ≤ 60 days	10	WMD −1.97 (−2.36, −1.58)	<0.001	66.7	<0.01
	Duration of intervention > 60 days	1	WMD −1.88 (−3.13, −0.63)	<0.01	—	—
CHM plus WCM vs. WCM		4	WMD −1.67 (−2.34, −1.00)	<0.001	81.2	<0.01
CHM vs. GET		1	WMD −1.23 (−1.96, −0.50)	<0.01	—	—
CHM vs. placebo		3	WMD −1.84 (−1.98, −1.70)	<0.001	54.8	= 0.109
CHM vs. health guidance		1	WMD −3.14 (−3.83, −2.45)	<0.001	—	—
Subgroup analyses for FAI scores						
CHM vs. WCM		7	WMD −15.49 (−28.39, −2.60)	= 0.019	99.6	<0.001
	Duration of intervention ≤ 30 days	2	WMD -32.15 (−33.76, −30.53)	<0.001	0	= 0.829
	30 days < duration of intervention ≤ 60 days	2	WMD −18.24 (−28.11, −8.38)	<0.001	40.9	= 0.193
	Duration of intervention > 60 days	3	WMD −2.88 (−6.15, 0.38)	= 0.084	87.7	<0.001
CHM plus WCM vs. WCM		1	WMD −12.04 (−16.96, −7.12)	<0.001	—	—
CHM vs. GET		1	WMD −21.80 (−33.42, −10.18)	<0.001	—	—
Subgroup analyses for SCL-90 scores						
CHM vs. WCM		4	WMD −9.52 (−12.08, −6.97)	<0.001	0	= 0.533
CHM vs. placebo		1	WMD −27.71 (−52.10, −3.32)	= 0.026	—	—
Subgroup analyses for SAS scores						
CHM vs. WCM		3	WMD −9.14 (−17.31, −0.97)	= 0.028	95.4	<0.001
CHM plus WCM vs. WCM		2	WMD −4.55 (−7.51, −1.58)	<0.01	68.3	= 0.076
	Duration of intervention ≤ 30 days	1	WMD −5.94 (−7.98, −3.90)	<0.001	—	—
	30 days < duration of intervention ≤ 60 days	1	WMD −2.90 (−5.56, −0.24)	= 0.033	—	—
CHM vs. placebo		1	WMD −6.77 (−7.08, −6.46)	<0.001	—	—
CHM vs. health guidance		1	WMD −6.55 (−8.84, −4.26)	<0.001	—	—
Subgroup analyses for SDS scores						
CHM vs. WCM		2	WMD −5.97 (−10.67, −1.28)	= 0.013	86.6	<0.01
CHM plus WCM vs. WCM		2	WMD −4.17 (−7.07, −1.27)	<0.01	64	= 0.095
	Duration of intervention ≤ 30 days	1	WMD −5.69 (−8.25, −3.13)	<0.001	—	—
	30 days < duration of intervention ≤ 60 days	1	WMD −2.73 (−5.09, −0.37)	= 0.023	—	—
CHM vs. placebo		1	WMD −6.42 (−6.65, −6.19)	<0.001	—	—
CHM vs. health guidance		1	WMD −4.23 (−5.72, −2.74)	<0.001	—	—
Clinical symptom scores						
CHM vs. WCM		18	WMD −6.60 (−7.89, −5.31)	<0.001	96.9	<0.001
	Duration of intervention ≤ 30 days	11	WMD −8.40 (−11.00, −5.80)	<0.001	97.1	<0.001
	30 days < duration of intervention ≤ 60 days	3	WMD −3.37 (−4.77, −1.96)	<0.001	80.5	<0.01
	Duration of intervention > 60 days	1	WMD −5.22 (−5.69, −4.75)	<0.001	—	—
CHM plus WCM vs. WCM		2	WMD −2.82 (−3.45, −2.20)	<0.001	47.3	= 0.168
CHM vs. placebo		3	WMD −3.13 (−3.99, −2.27)	<0.001	95.2	<0.001
CHM vs. health guidance		1	WMD −6.73 (−7.57, −5.89)	<0.001	—	—
Subgroup analyses for IGA						
CHM vs. WCM		7	WMD 0.31 (0.18, 0.43)	<0.001	75.9	<0.001
	Duration of intervention ≤ 30 days	1	WMD 0.24 (0.06, 0.42)	<0.01	—	—
	30 days < duration of intervention ≤ 60 days	3	WMD 0.38 (0.23, 0.54)	<0.001	73.2	= 0.024
	Duration of intervention > 60 days	3	WMD 0.21 (−0.12, 0.53)	= 0.218	86.1	<0.01
CHM vs. placebo		1	WMD 0.26 (0.20, 0.32)	<0.001	—	—
Subgroup analyses for IGG						
CHM vs. WCM		7	WMD 2.21 (0.90, 3.51)	<0.01	94.0	<0.001
	Duration of intervention ≤ 30 days	1	WMD 20.61 (15.97, 25.25)	<0.001	—	—
	30 days < duration of intervention ≤ 60 days	3	WMD 1.30 (0.23, 2.38)	= 0.018	80.5	<0.01
	Duration of intervention > 60 days	3	WMD 0.95 (−0.36, 2.27)	= 0.154	89.1	<0.001
CHM vs. placebo		1	WMD 1.39 (1.22, 1.56)	<0.001	—	—
Subgroup analyses for IGM						
CHM vs. WCM		7	WMD 0.20 (0.10, 0.29)	<0.001	69.3	<0.01
	Duration of intervention ≤ 30 days	1	WMD 0.02 (−0.10, 0.15)	= 0.712	—	—
	30 days < duration of intervention ≤ 60 days	3	WMD 0.24 (0.16, 0.33)	<0.001	25.4	= 0.262
	Duration of intervention > 60 days	3	WMD 0.21 (0.03, 0.38)	= 0.019	68.1	= 0.044
CHM vs. placebo		1	WMD 0.28 (0.24, 0.32)	<0.001	—	—
Subgroup analyses for NK cell						
CHM vs. WCM		2	WMD 0.94 (−1.14, 3.03)	= 0.376	0	= 0.613
CHM vs. Placebo		1	WMD 0.94 (−1.14, 3.03)	<0.001	—	—
Effective Rate						
CHM vs. WCM		59	RR 1.43 (1.33, 1.52)	<0.001	76.6	<0.001
	Duration of intervention ≤ 30 days	25	RR 1.64 (1.43, 1.89)	<0.001	82.6	<0.001
	30 days < duration of intervention ≤60 days	19	RR 1.38 (1.23, 1.54)	<0.001	77	<0.001
	Duration of intervention > 60 days	11	RR 1.28 (1.20, 1.37)	<0.001	0	= 0.590
CHM plus WCM vs. WCM		12	RR 1.20 (1.13, 1.27)	<0.001	0	= 0.762
	Duration of intervention ≤ 30 days	7	RR 1.22 (1.14, 1.32)	<0.001	0	= 0.918
	30 days < duration of intervention ≤ 60 days	3	RR 1.13 (0.99, 1.28)	= 0.062	31.1	= 0.234
CHM vs. GET		1	RR 1.16 (0.98, 1.38)	= 0.093	—	—
CHM plus GET vs. GET		2	RR 1.39 (1.06, 1.83)	= 0.019	56.1	= 0.131
CHM vs. placebo		4	RR 2.54 (2.00, 3.22)	<0.001	0	= 0.943
CHM vs. health guidance		1	RR 6.59 (2.54, 17.12)	<0.001	—	—

FS-14: Fatigue Scale; FAI: Fatigue Assessment Instrument; SCL-90: Self-Rating Scale of mental state; SAS: Self-Rating Anxiety Scale; SDS: Self-Rating Depression Scale; CHM: Chinese herbal medicine; WCM: western conventional medicine; GET: graded exercise therapy; WMD: weighted mean difference; RR: relative risk.

### FAI scores

**FIGURE 3:**
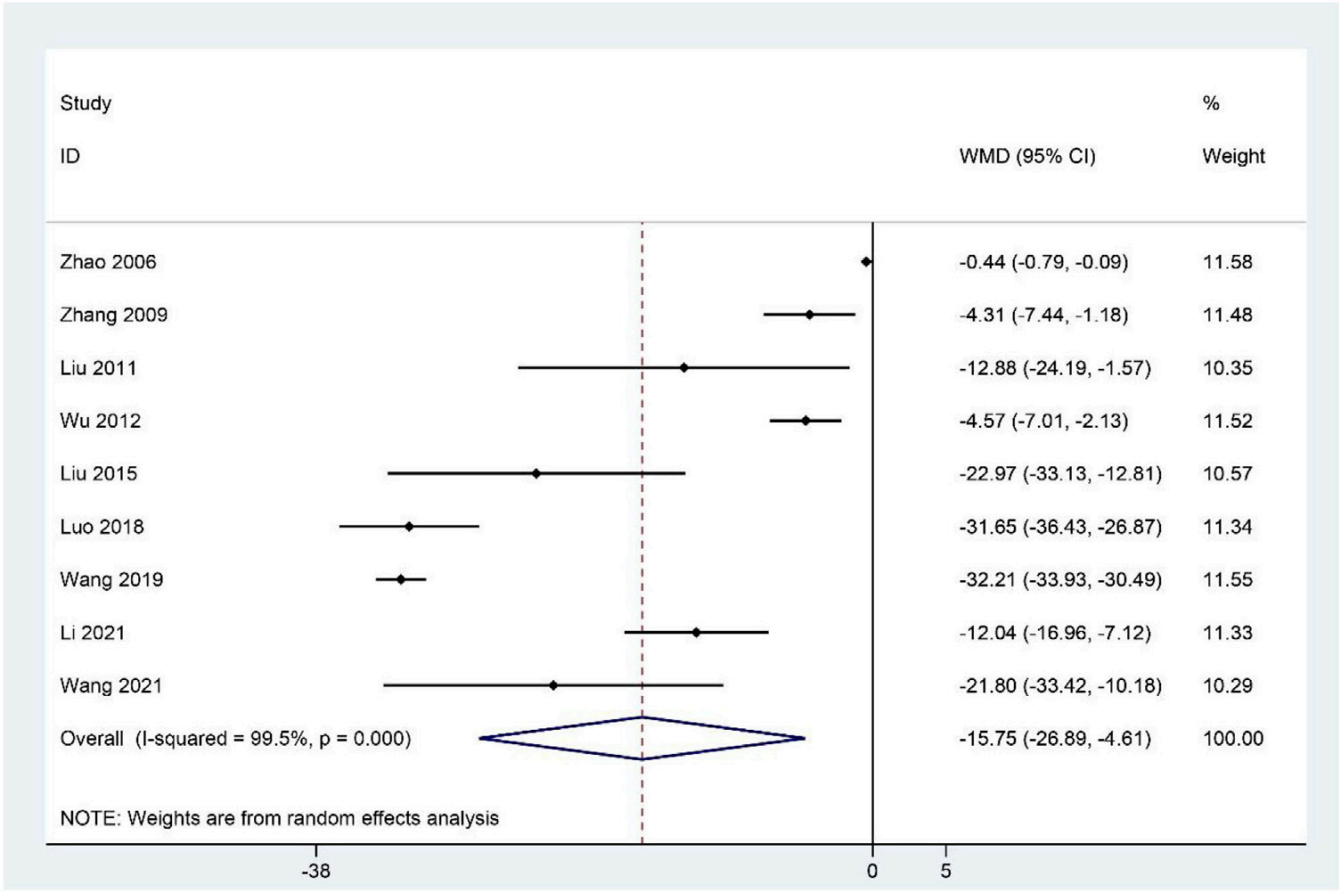
Forest plot for FAI scores.

Meta-analysis of the nine studies (Zhao et al., 2006; Zhang et al., 2009; Liu et al., 2011; Wu et al., 2012; Liu et al., 2015; Luo, 2018; Wang, 2019; Li et al., 2021; Wang, 2021) reporting the FAI scores showed that the treatment group had significantly decreased FAI scores compared to the control group (WMD: –15.75; 95%CI: –26.89 to –4.61; *p <* 0.01; *p* for heterogeneity <0.001; I^2^ = 99.5%; Figure 3). Subgroup analysis revealed no significant difference (WMD: –2.88; 95%CI: –6.15 to 0.38; *p* = 0.084; *p* for heterogeneity <0.001; I^2^ = 87.7%) between CHM and WCM groups when the duration of intervention >60 days, whereas the relationship between the CHM treatment group and lower FAI scores remained constant in the other subgroups (Table 4).

### Secondary outcomes

#### SCL-90 scores

**FIGURE 4:**
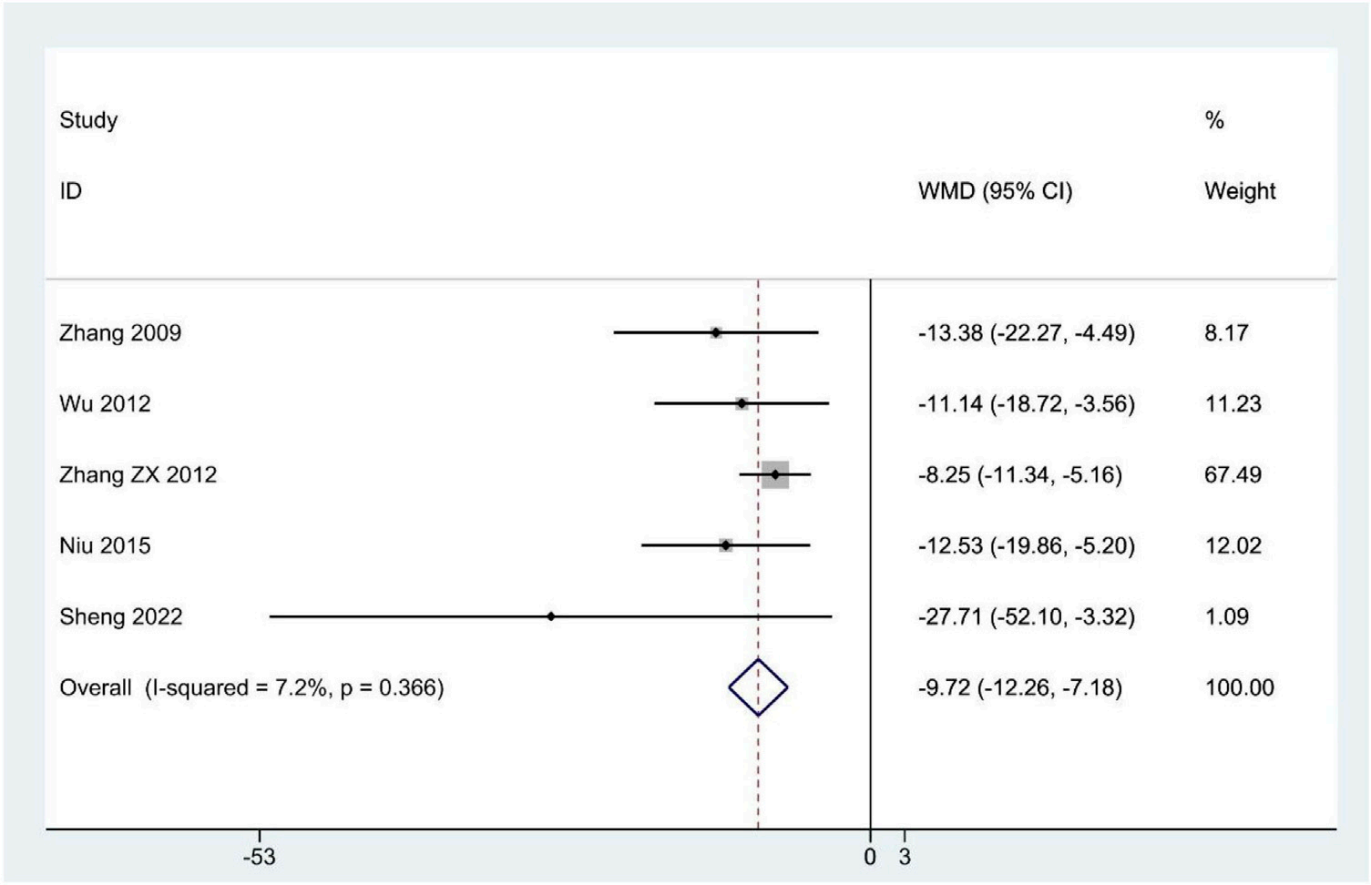
Forest plot for SCL-90 scores.

The SCL-90 scores were reported in five studies (Zhang et al., 2009; Zhang Z. X. et al., 2012; Wu et al., 2012; Niu et al., 2015; Sheng et al., 2022). The pooled results suggested that SCL-90 scores were significantly lower in the CHM group compared to the contrast group (WMD: –9.72; 95%CI: –12.26 to –7.18; *p* < 0.001; *p* for heterogeneity = 0.366; I^2^ = 7.2%; Figure 4), and the subgroup analysis showed similar results (Table 4).

### SAS scores

**FIGURE 5:**
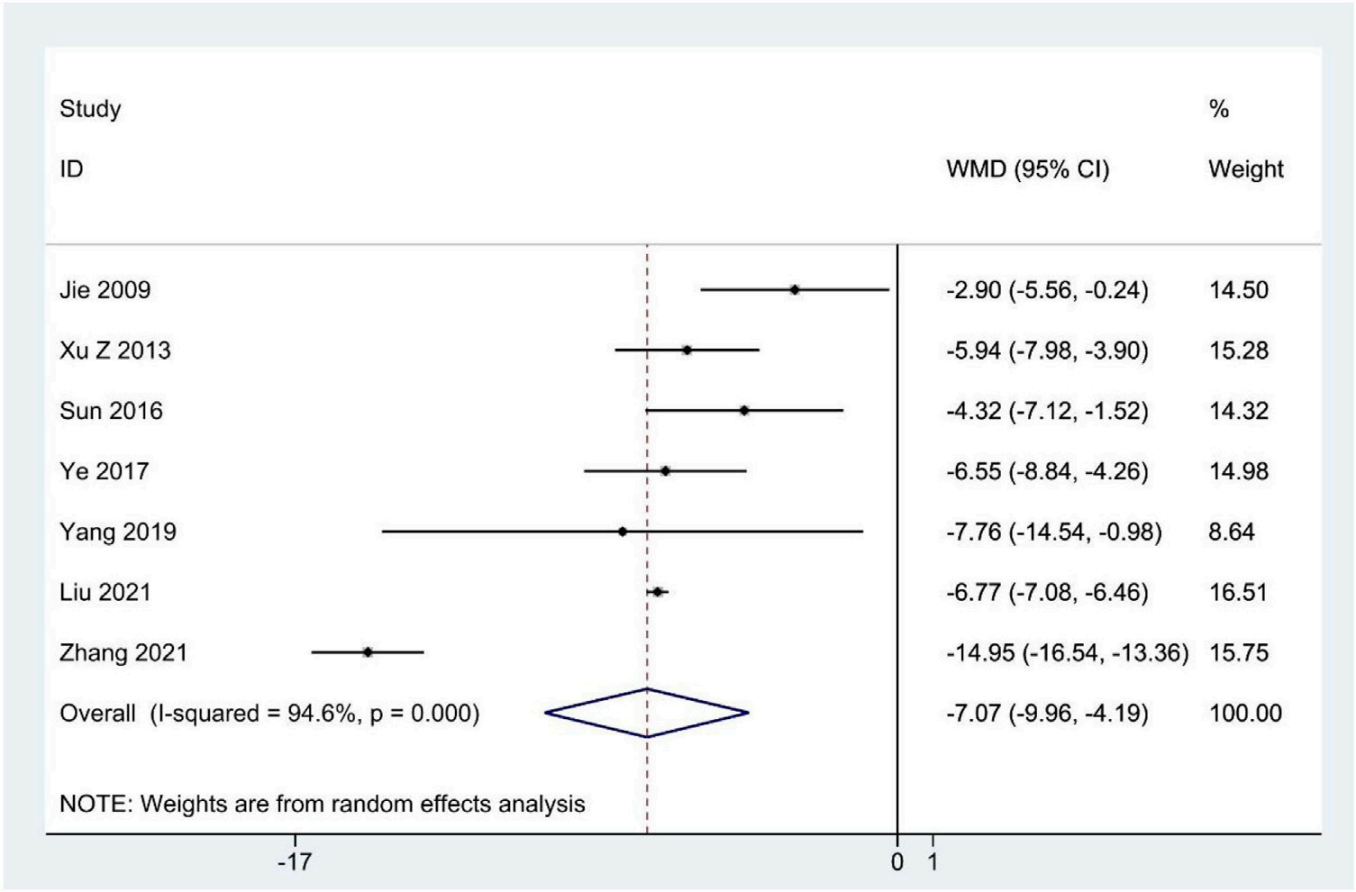
Forest plot for SAS scores.

Seven studies (Jie and Wang, 2009; Xu and Wang, 2013; Sun et al., 2016; Ye, 2017; Yang, 2019; Liu et al., 2021; Zhang, 2021) reported the SAS scores, and meta-analysis indicated that CHM therapy clearly decreased SAS scores compared to the contrast group (WMD: –7.07; 95%CI: –9.96 to –4.19; *p* < 0.001; *p* for heterogeneity <0.001; I^2^ = 94.6%; Figure 5), and subgroup analysis showed that the results remained constant (Table 4).

### SDS scores

**FIGURE 6:**
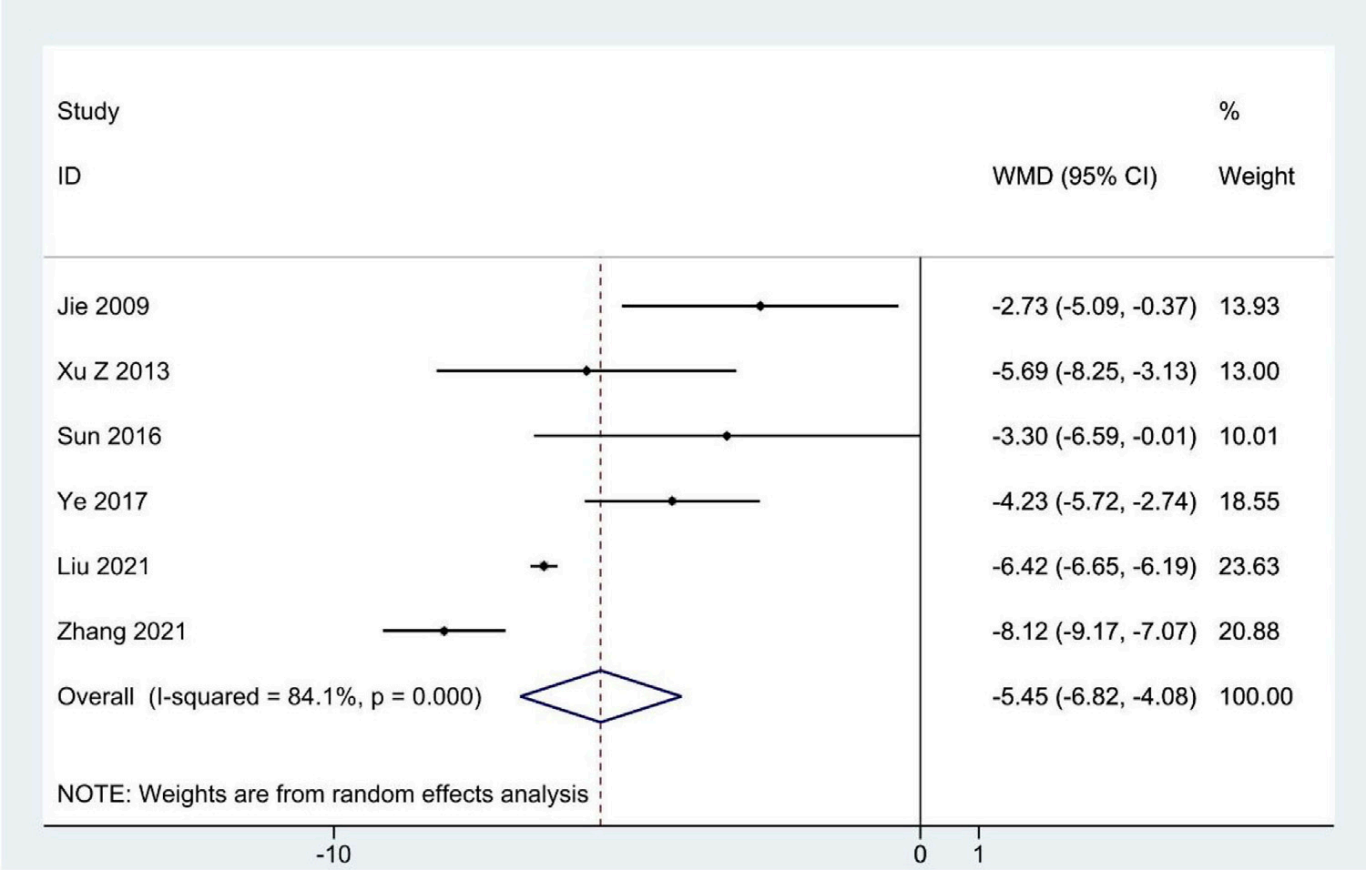
Forest plot for SDS scores.

Meta-analysis of six RCTs reporting the SDS scores (Jie and Wang, 2009; Xu and Wang, 2013; Sun et al., 2016; Ye, 2017; Liu et al., 2021; Zhang, 2021) showed that the experimental group had significantly reduced SDS scores compared to the contrast group (WMD: –5.45; 95%CI: –6.82 to –4.08; *p* < 0.001; *p* for heterogeneity <0.001; I^2^ = 84.1%; Figure 6). Subgroup analysis showed that the results remained constant (Table 4).

### Clinical symptom scores

**FIGURE 7:**
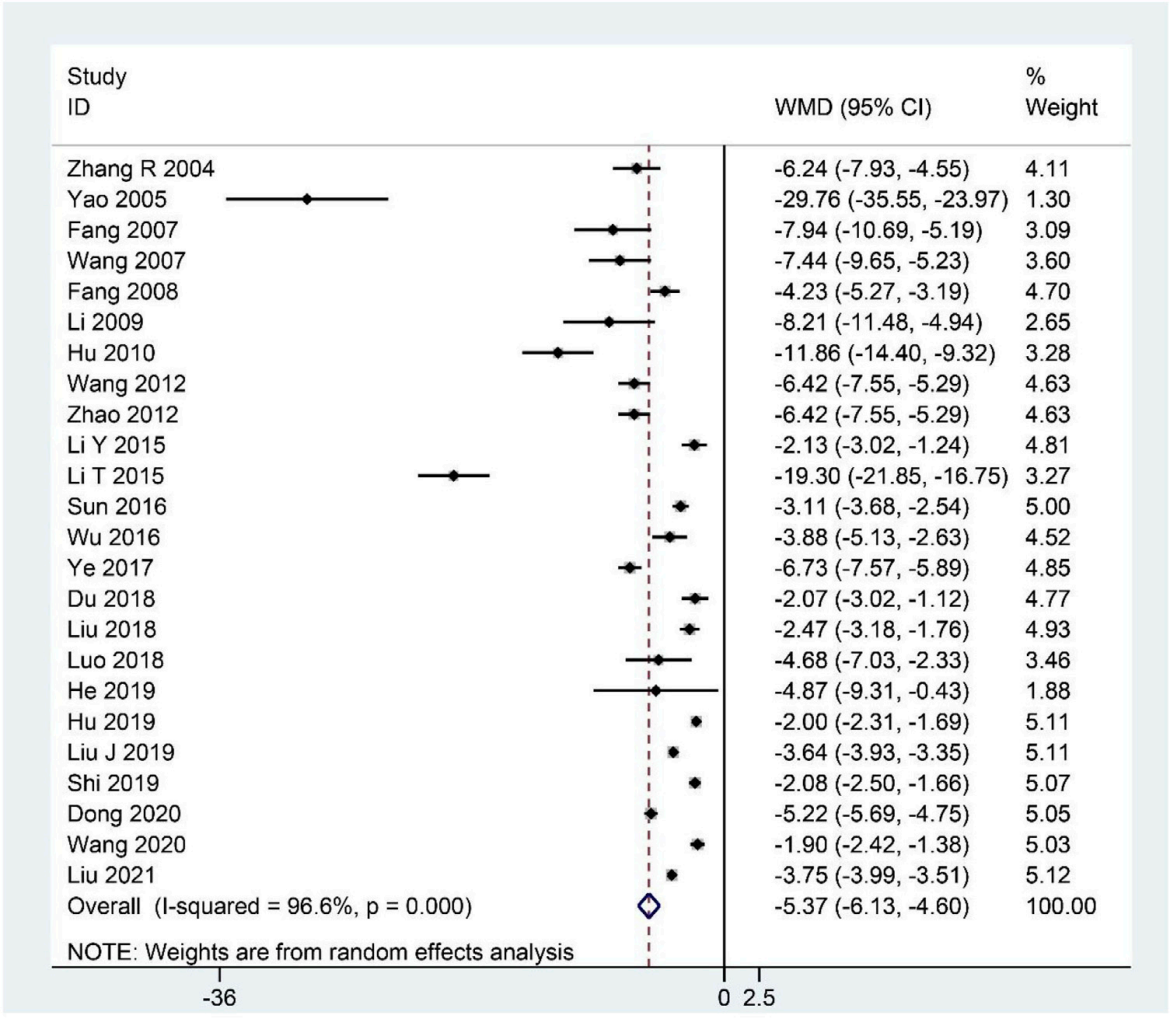
Forest plot for clinical symptom scores.

The summary data of 24 studies (Zhang et al., 2004; Yao and Qiu, 2005; Fang et al., 2007; Wang et al., 2007; Fang et al., 2008; Li, 2009; Hu et al., 2010; Wang, 2012; Zhao, 2012; Li, 2015; Li and Zao, 2015; Sun et al., 2016; Wu et al., 2016; Ye, 2017; Du, 2018; Liu and Cai, 2018; Luo, 2018; Liu J. et al., 2019; He, 2019; Hu, 2019; Shi, 2019; Dong, 2020; Wang, 2020; Liu et al., 2021) demonstrated that CHM, as an adjuvant or monotherapy, significantly decreased the clinical symptom scores compared with the control group (WMD: –5.37; 95%CI: –6.13 to –4.60; *p* < 0.001; *p* for heterogeneity <0.001; I^2^ = 96.6%; Figure 7). Subgroup analysis was performed, showing that the conclusion that CHM is relatively effective in treating CFS remained unchanged in each subgroup (Table 4).

### Immunological indicators

**FIGURE 8:**
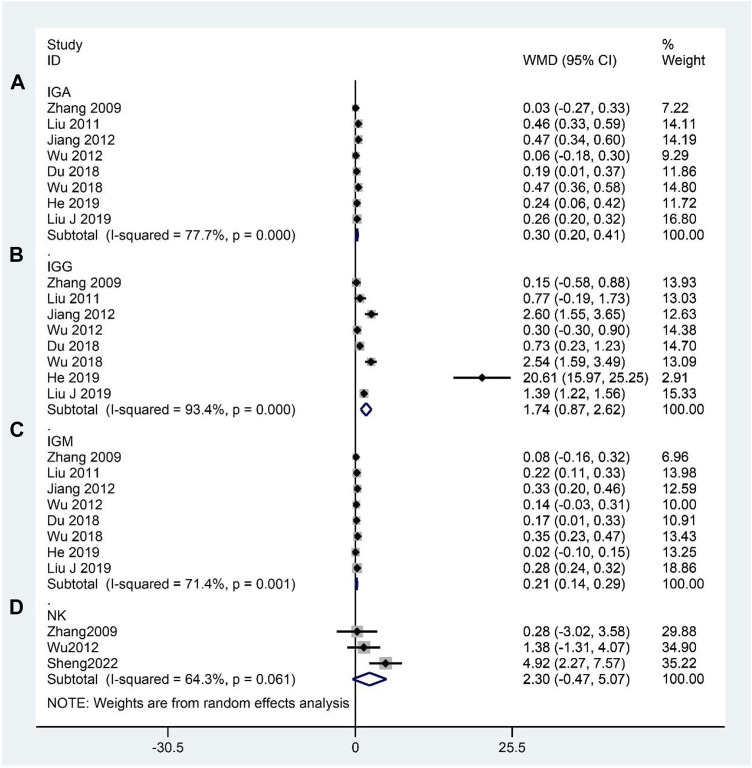
Forest plot for immunological indicators: **(A)** IGA, **(B)** IGG, **(C)** IGM, and **(D)** NK cell.

We identified eight RCTs that reported the IGA, IGG, and IGM levels (Zhang et al., 2009; Liu et al., 2011; Jiang, 2012; Wu et al., 2012; Du, 2018; Wu et al., 2018; Liu J. et al., 2019; He, 2019). Meta-analysis indicated that CHM significantly elevated IGA (WMD: 0.30; 95%CI: 0.20–0.41; *p <* 0.001; *p* for heterogeneity <0.001; I^2^ = 77.7%; Figure 8A); IGG (WMD: 1.74; 95%CI: 0.87–2.62; *p <* 0.001; *p* for heterogeneity <0.001; I^2^ = 93.4%; Figure 8B); and IGM (WMD: 0.21; 95%CI: 0.14–0.29; *p <* 0.001; *p* for heterogeneity <0.01; I^2^ = 71.4%; Figure 8C) compared to the contrast group. Three studies (Zhang et al., 2009; Wu et al., 2012; Sheng et al., 2022) reported the NK cell levels, and the meta-analysis indicated no significant difference between the experimental and control groups (WMD: 2.30; 95%CI: –0.47 to 5.07; *p* = 0.104; *p* for heterogeneity = 0.061; I^2^ = 64.3%; Figure 8D). Subgroup analyses of the IGA and IGG revealed no significant difference (WMD: 0.21; 95%CI: –0.12 to 0.53; *p* = 0.218; *p* for heterogeneity <0.01; I^2^ = 86.1%, WMD: 0.95; 95%CI: –0.36 to 2.27; *p* = 0.154; *p* for heterogeneity <0.001; I^2^ = 89.1%, respectively) between the CHM and WCM groups when the duration of intervention >60 days, and the subgroup analysis of the IGM showed no statistical significance (WMD: 0.02; 95%CI: –0.10 to 0.15; *p* = 0.712; no heterogeneity) between the CHM and WCM groups when the duration of intervention ≤30 days. The rest of the results indicated that the conclusion that CHM can elevate IGA, IGG, and IGM remained constant (Table 4). The subgroup analysis of the NK cell showed no statistical significance (WMD: 0.94; 95%CI: –1.14 to 3.03; *p* = 0.376; *p* for heterogeneity = 0.613; I^2^ = 0.0%) between the CHM and WCM groups, whereas one study comparing CHM with placebo showed that CHM significantly elevated the NK cell levels (WMD: 4.92; 95%CI: 2.27–7.57; *p* < 0.001; no heterogeneity) (Table 4).

### Effective rate

**FIGURE 9:**
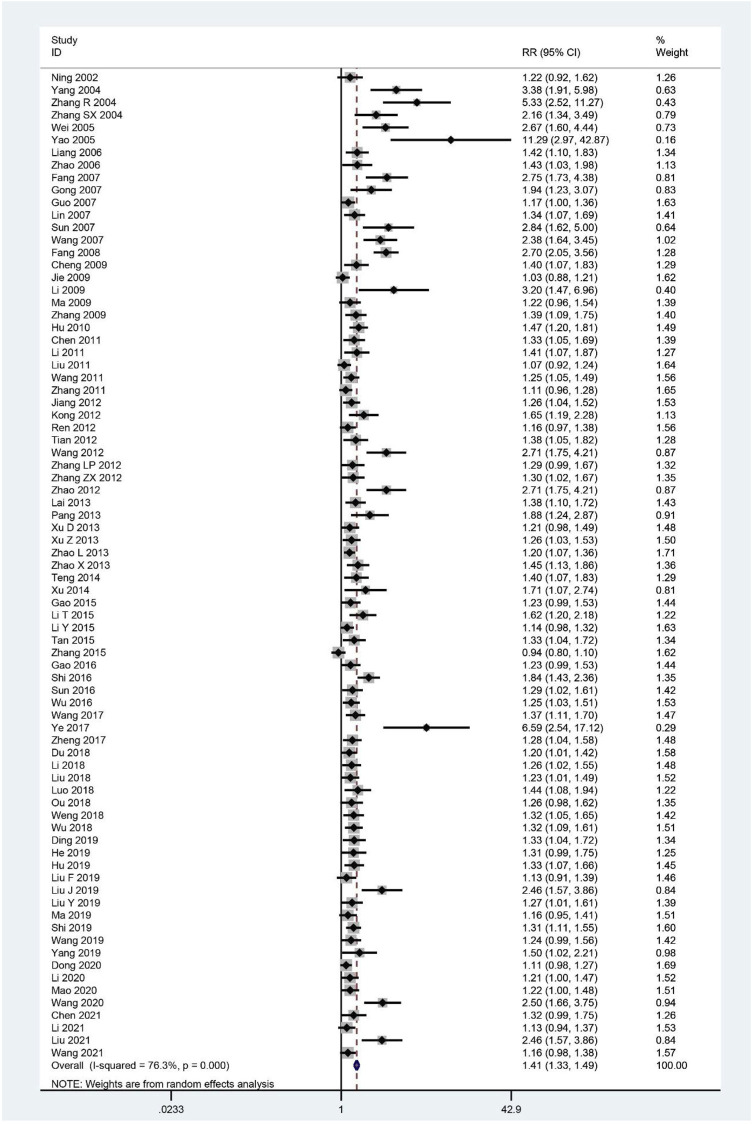
Forest plot for effective rate.

The effective rate was evaluated in 79 trials (Ning and Li, 2002; Yang et al., 2004; Zhang et al., 2004; Zhang and Zhou, 2004; Wei, 2005; Yao and Qiu, 2005; Liang, 2006; Zhao et al., 2006; Fang et al., 2007; Gong, 2007; Guo et al., 2007; Lin, 2007; Sun et al., 2007; Wang et al., 2007; Fang et al., 2008; Cheng, 2009; Jie and Wang, 2009; Li, 2009; Ma, 2009; Zhang et al., 2009; Hu et al., 2010; Chen et al., 2011; Li et al., 2011; Liu et al., 2011; Wang et al., 2011; Zhang et al., 2011; Jiang, 2012; Kong, 2012; Ren and Yu, 2012; Tian and Wang, 2012; Wang, 2012; Zhang Z. X. et al., 2012; Zhang L. P. et al., 2012; Zhao, 2012; Lai and Lei, 2013; Pang and Liu, 2013; Xu et al., 2013; Xu and Wang, 2013; Zhao, 2013; Zhao et al., 2013; Teng et al., 2014; Xu, 2014; Gao and Pang, 2015; Li and Zao, 2015; Li, 2015; Tan et al., 2015; Zhang et al., 2015; Gao and Pang, 2016; Shi and Wu, 2016; Sun et al., 2016; Wu et al., 2016; Wang, 2017; Ye, 2017; Zheng et al., 2017; Du, 2018; Li et al., 2018; Liu and Cai, 2018; Ou et al., 2018; Weng, 2018; Wu et al., 2018; Luo, 2018; Ding, 2019; He, 2019; Hu, 2019; Liu F. et al., 2019; Liu J. et al., 2019; Liu Y. et al., 2019; Ma et al., 2019; Shi, 2019; Wang, 2019; Yang, 2019; Dong, 2020; Li, 2020; Mao, 2020; Wang, 2020; Chen, 2021; Li et al., 2021; Liu et al., 2021; Wang, 2021), and the pooled results showed that it was higher in the treatment group compared to the control group (RR = 1.41; 95%CI: 1.33–1.49; *p <* 0.001; *p* for heterogeneity <0.001; I^2^ = 76.3%; Figure 9). Subgroup analysis showed no significant difference (RR: 1.13; 95%CI: 0.99 to 1.28; *p* = 0.062; *p* for heterogeneity = 0.234; I^2^ = 31.1%) in CHM plus WCM compared with WCM when 30 days < intervention time ≤ 60 days, and only one study compared CHM and GET, showing similar results (RR: 1.16; 95%CI: 0.98–1.38; *p* = 0.093; no heterogeneity), whereas the rest of the results revealed that effectiveness of CHM for CFS remained constant (Table 4).

### Adverse events

Adverse events were reported in 14 studies (Liang, 2006; Gong, 2007; Lin, 2007; Wang et al., 2007; Jie and Wang, 2009; Li, 2009; Li et al., 2011; Xu and Wang, 2013; Zhang et al., 2015; Sun et al., 2016; Wu et al., 2016; Ye, 2017; Li et al., 2018; Liu and Cai, 2018), and two studies (Sun et al., 2007; Wang, 2017) reported that no adverse events occurred. The rest of the studies did not report the presence or absence of adverse events. The adverse events in the CHM group included mild nausea, dry mouth, indigestion, constipation, and fever. The majority of adverse events were mild, and serious adverse events or deaths were not found in the included studies, which suggests that CHM is relatively safe in patients with CFS.

### Sensitivity analysis

We conducted a sensitivity analysis on FS-14, FAI, SAS, SDS, clinical symptom scores, IGA, IGG, IGM, NK cell levels, and effective rate. After we excluded each study one by one, the pooled WMD or RR for the rest of the RCTs did not change significantly, indicating that the result data were robust (Additional file 2).

### Publication bias

The funnel plot showed a symmetric distribution of trials on either side of the funnel, and Egger’s test (*p* = 0.795) was consistent with the funnel plot, indicating that no significant publication bias existed in this meta-analysis (Additional file 3).

### Description of the CHM

Our study evaluated 69 kinds of Chinese herbal formulas, including 54 decoctions, five granules, three oral liquids, three pills, two ointments, one capsule, and one herbal porridge. The most frequently used herbs in all formulations contained Chai Hu (*Bupleurum falcatum* L.); Gan Cao (*Glycyrrhiza glabra* L.); Bai Zhu (*Atractylodes macrocephala* Koidz.); Dang Gui [*Angelica sinensis* (Oliv.) Diels]; Huang Qi (*Astragalus mongholicus* Bunge); Dang Shen [*Codonopsis pilosula* (Franch.) Nannf.]; Bai Shao (*Paeonia lactiflora* Pall.); Fu Ling [*Poria cocos* (Schw.) Wolf]; Chen Pi (*Citrus × Aurantium* L.); Shu Di (*Rehmannia glutinosa* (Gaertn.) DC.); Chuan Xiong (*Conioselinum anthriscoides* “Chuanxiong”); Yu Jin (*Curcuma aromatica* Salisb.); Shan Yao (*Dioscorea oppositifolia* L.); Yuan Zhi (*Polygala tenuifolia* Willd.); Ban Xia [*Pinellia ternata* (Thunb.) Makino]; Gou QI (*Lycium chinense* Mill.); Da Zao (*Ziziphus jujuba* Mill.); Zhi Qiao (*Citrus × Aurantium* L.); Suanzaoren (*Ziziphi Spinosae Semen*); Huang Qin (*Scutellaria baicalensis* Georgi); Ren Shen (*Panax ginseng* C.A.Mey.); and Wu Wei Zi [*Schisandra chinensis* (Turcz.) Baill.] (Table 5).

**TABLE 5:** Frequently used herbs in included studies.

Chinese name	Accepted scientific name	English name	Family	Number of studies (%)
Gan Cao	*Glycyrrhiza glabra* L.	Liquorice root	Leguminosae	53 (63%)
Bai Zhu	*Atractylodes macrocephala* Koidz	largehead atractylodes rhizome	Asteraceae	50 (60%)
Huang Qi	*Astragalus mongholicus* Bunge	Milkvetch root	Leguminosae	50 (60%)
Chai Hu	*Bupleurum falcatum* L.	Chinese thorowax root	Umbelliferae	48 (57%)
Fu Ling	*Poria cocos* (Schw.) Wolf	Indian bread	Polyporaceae	48 (57%)
Dang Gui	*Angelica sinensis* (Oliv.) Diels	Chinese angelica	Umbelliferae	45 (54%)
Dang Shen	*Codonopsis pilosula* (Franch.) Nannf.	Tangshen	Campanulaceae	37 (44%)
Bai Shao	*Paeonia lactiflora* Pall.	Debark peony root	Ranunculaceae	30 (36%)
Chen Pi	*Citrus × aurantium* L.	Dried tangerine peel	Rutaceae	25 (30%)
Ren Shen	*Panax ginseng* C.A.Mey	Ginseng	Araliaceae	23 (27%)
Shu Di	*Rehmannia glutinosa* (Gaertn.) DC.	Prepared rehmannia root	Scrophulariaceae	23 (27%)
Chuan Xiong	*Conioselinum anthriscoides* “Chuanxiong”	Chuanxiong	Umbelliferae	20 (24%)
Yu Jin	*Curcuma aromatica* Salisb.	Turmeric root tuber	Zingiberaceae	19 (23%)
Ban Xia	*Pinellia ternata* (Thunb.) Makino	Pinellia tuber	Araceae	16 (19%)
Yuan Zhi	*Polygala tenuifolia* Willd	Milkwort root	Polygalaceae	15 (18%)
Gou QI	*Lycium chinense* Mill.	Barbary wolfberry fruit	Solanaceae	14 (17%)
Shan Yao	*Dioscorea oppositifolia* L.	Common yam rhizome	Dioscoreaceae	13 (15%)
SuanZao Ren	*Ziziphi Spinosae Semen*	Spine date seed	Rhamnaceae	13 (15%)
Zhi Qiao	*Citrus × aurantium* L.	Bitter orange	Rutaceae	13 (15%)
Huang Qin	*Scutellaria baicalensis* Georgi	Baical skullcap root	Lamiaceae	13 (15%)
Da Zao	*Ziziphus Jujuba* Mill.	Chinese date	Rhamnaceae	11 (13%)
Wu Wei Zi	*Schisandra chinensis* (Turcz.) Baill.	Chinese magnoliavine fruit	Magnoliaceae	11 (13%)

## Discussion

Medically unexplained chronic fatigue, including idiopathic chronic fatigue and CFS, is an unexplained adverse condition characterized by fatigue accompanied by behavioral, emotional, social, and cognitive imbalances. Approximately 10% of the general population suffers from chronic fatigue, which significantly reduces their quality of life and their ability to work. This is an important health care issue, presenting major challenges for its sufferers and health services. At present, a clear therapeutic approach is still lacking, but the use of CHM in patients with chronic fatigue is receiving increasing attention from physicians.

### Summary of the evidence

A total of 84 RCTs, including 6,944 individuals, were identified for analysis. The findings demonstrated that CHM as adjuvant therapy or monotherapy for CFS could decrease the FS-14, FAI, SCL-90, SAS, SDS, and clinical symptom scores and improve IGA, IGG, IGM, and the effective rate.

Two internationally recognized scales were used to quantitatively assess fatigue. The FS-14 developed by Trudie Chalde et al. in 1993 consists of 14 items, each of which is a fatigue-related question, and it mainly reflects the changes in fatigue symptoms from two different perspectives (physical fatigue and mental fatigue), thus reflecting the real level of fatigue of patients in a more comprehensive way. The FAI includes 27 fatigue-related questions. Subjects rate each item based on their own performance over the previous 2 weeks, which can accurately and quantitatively evaluate the degree and characteristics of fatigue. In this study, CHM treatment significantly reduced FS-14 and FAI scores, indicating that it improved fatigue symptoms.

Patients with CFS commonly suffer from negative emotions such as anxiety, depression, paranoia, and obsessive-compulsive disorder. The degree of negative emotions is mainly assessed by professional mental status assessment scales such as SCL-90, SAS, and SDS. The present meta-analysis shows that CHM treatment can relatively improve negative emotions in patients with chronic fatigue.

The clinical effective rate and clinical symptom scores were used to evaluate the efficacy of CHM in the treatment of CFS because the severity of clinical symptoms is used to determine whether the disease is in remission. The clinical efficiency rate in patients treated with CHM alone or with CHM plus other treatments (e.g., WCM, GET, or health guidance) was 90% (2,961/3,308). The clinical efficiency rate in patients treated only with WCM, GET, health guidance, or placebo was 62% (1,956/3,149). Thus, CHM treatment clearly increased the efficiency rate and reduced the clinical symptom scores compared to WCM, GET, health guidance, or placebo, thus showing that CHM is effective to some extent for CFS.

Numerous studies have revealed that CFS is associated with immune system dysfunction (Matsuda et al., 2009; Guenther et al., 2015; Hornig et al., 2015; Montoya et al., 2017; Sotzny et al., 2018). Most CFS patients are prone to physical weakness and fatigue due to low immune function. Moreover, when the body tissue is in a state of fatigue for a long time, it will consume and destroy the immune system, which will eventually lead to low immune function. Immunoglobulins (IGA, IGG, and IGM) are important parts of humoral immunity. A study found that IGA, IGG, and IGM levels are significantly lower in patients with CFS than in healthy subjects (Hou et al., 2015). Our meta-analysis showed that the treatment group had elevated IGA, IGG, and IGM levels compared to the control group. In addition, immunological indicators also include NK cells and T lymphocyte subsets (CD4^+^, CD8^+^, and CD4+/CD8+). Hou’s study showed that NK cell activity, CD4^+^, and CD8^+^ were all significantly reduced in CFS patients (Hou et al., 2015). The results of our meta-analysis did not find any obvious effects of CHM on NK cell activity in CFS patients. However, three trials (Zhang et al., 2009; Wu et al., 2012; Sheng et al., 2022) suggested that CFS patients’ NK cell activity was higher in the CHM treatment group. We cannot reject the positive effect of CHM on the NK cell activity of CFS patients based on the negative results of this meta-analysis, which may be due to the lack of appropriate courses of treatment and limited sample sizes. Furthermore, a study showed that CHM dramatically improved NK cell activities, T cell proliferation, CD4 +/CD8 + ratio, and CD4 + counts in CFS rats, suggesting that CHM can improve the immune function of patients with CFS (Chi et al., 2015). Taken together, CHM may prevent CFS by modulating immune function, but further research is needed to confirm this.

Furthermore, only 14 studies referred to minor adverse reactions, and there were no serious adverse events, showing that CHM generally appears safe and effective for treating CFS. Thus, the present evidence supports that CHM can potentially be recommended for use in CFS patients.

### Strengths and limitations

Our study included a large number of RCTs and large sample sizes (84 RCTs with 6,944 patients) and used more internationally recognized outcome measures to assess the effectiveness of CHM for CFS from different aspects. These outcome indicators included not only subjective indicators (FS-14, FAI, SCL-90, SAS, SDS, clinical symptom scores, and effective rate), but also the objective immune indicators IGA, IGG, IGM, NK cell levels, and adverse events. In addition, we included many new trials that were not included in the previous reviews and meta-analyses to provide a comprehensive update. Furthermore, the sensitivity analysis demonstrated that the results of the current meta-analysis are relatively robust, and we found no evidence of publication bias in this meta-analysis by funnel plot and Egger’s test.

Some limitations must be considered. First, although we included RCTs, some methodological limitations still existed in most studies. Specifically, 44 trials supplied sufficient information on the randomization process, only five RCTs described allocation concealment, only three trials reported double blinding of patients and physicians, and only eight trials described blinding of participants. These methodological flaws might generate bias, so our results should be interpreted cautiously. Second, there is significant clinical heterogeneity due to the variations in composition and dosage of CHM and different dosage forms of CHM (e.g., decoction, granule, oral liquid, pill, ointment, and capsule). Finally, all trials were conducted in China, which may limit the generalizability of the findings presented here. Therefore, further international multicenter RCTs are needed to popularize the results globally. Furthermore, we conducted subgroup analyses to explore the sources of heterogeneity based on the different intervention duration and measures. The result showed that the heterogeneity was lower after grouping according to the results of subgroup analyses, indicating that differences in intervention duration and measures may also be the underlying source of heterogeneity.

### Implications for research

Based on the above limitations, some recommendations are suggested for further studies. First, further rigorously designed trials with high methodological quality are urgently needed. We advise designing and reporting RCTs of CFS strictly according to the CONSORT 2010 statement (Schulz et al., 2010) and the CONSORT Extension for Chinese Herbal Medicine Formulas 2017 (Cheng et al., 2017). Random sequence generation, allocation concealment, and blinding should all be strictly implemented in future studies. Second, an efficacy evaluation system in line with the characteristics of CHM should be set up, and sensitive and practical indicators of CHM should be explored. Third, adverse effects were not reported in many studies. Therefore, the presence or absence of adverse events should be reported in future studies based on the standard format of adverse reactions established by Bian et al. (2010), and clinical trials and studies with longer follow-up times should be conducted to confirm the long-term safety of CHM for CFS.

### Implications for practice

The evidence available from our study suggested the effectiveness and safety of CHM therapy for CFS. The most commonly used herbs included *Bupleurum falcatum* L., *Glycyrrhiza glabra* L., *Atractylodes macrocephala* Koidz., *Angelica sinensis* (Oliv.) Diels, *Astragalus mongholicus* Bunge, *Codonopsis pilosula* (Franch.) Nannf., *Paeonia lactiflora* Pall., *Poria cocos* (Schw.) Wolf, *Citrus × aurantium* L., *Rehmannia glutinosa* (Gaertn.) DC., *Conioselinum anthriscoides* “Chuanxiong,” *Curcuma aromatica* Salisb., *Dioscorea oppositifolia* L., *Polygala tenuifolia* Willd., *Pinellia ternata* (Thunb.) Makino, *Lycium chinense* Mill., *Ziziphus jujuba* Mill., *Citrus × aurantium* L., *Ziziphi Spinosae Semen*, *Scutellaria baicalensis* Georgi, *Panax ginseng* C.A.Mey., and *Schisandra chinensis* (Turcz.) Baill. This list can facilitate further exploration of the therapeutic principles of these drugs for CFS in order to further develop herbal prescriptions to improve the efficacy and safety of the treatment of CFS. In addition, the efficacy of CHM depends on the accurate dialectical treatment, and the prescription of CHM should be based on the precise dialectical diagnosis of CFS. Thus, individualized herbal prescriptions can be implemented in future clinical practice by selecting appropriate drugs among the frequently used drugs.

The possible mechanisms of CHM for CFS are as follows. 1) Adjusting the immune dysfunction: a study found that Young Yum Pill, a proprietary herbal drug, could improve immune organ (thymus and spleen) indices, the mitogenic response of lymphocytes, and numbers of T-cell subsets (Yin et al., 2021). Buzhong Yiqi decoction, Kuibi decoction, and Danggui Buxue decoction significantly inhibit tumor necrosis factor-a, IL-6, IL-10, and transforming growth factor-b1 in CFS patients (Shin et al., 2004; Chen et al., 2010; Miao et al., 2022). Furthermore, Renshen Yangrong decoction can ameliorate lower NK cell activity, and extracts of Ginseng can also boost natural killer cell function and the cellular immunity of patients with CFS (Ogawa et al., 1992; See et al., 1997). 2) Antioxidant effects: superoxide dismutase (SOD) and glutathione peroxidase (GSH-Px) are two major components of the antioxidative system, and their function is to detoxify reactive oxygen species. Danggui Buxue decoction, Ginsenoside, and Jujube polysaccharide conjugate could improve SOD and GSH-Px activities and decrease MDA levels (Chi et al., 2015; Miao et al., 2022). Additionally, Quercetin, *Withania somnifera* (L.) Dunal, *Hypericum perforatum* L., and *Ginkgo biloba* L. have also been reported to possess beneficial antioxidants for CFS (Logan and Wong., 2001; Singh et al., 2002). 3) Improving metabolic dysfunction: Chi et al.’s study confirmed that SCP treatment affects metabolic pathways, including the TCA cycle and alanine, aspartate, and glutamate metabolism (Chi et al., 2016). Danggui Buxue decoction might regulate serine, glycine, and threonine metabolism to improve energy supply and ameliorate the CFS-weakened immunity (Miao et al., 2022). In addition, HEP2-a increased the creatine level to improve the arginine and proline metabolism (Chi et al., 2017). 4) Regulating the abnormal activity of the HPA axis: Chi inferred that HEP2-a indirectly affected the HPA axis abnormality of CFS by increasing the noradrenaline level (Chi et al., 2017).

## Conclusion

In conclusion, the current evidence suggests that CHM, either as adjuvant therapy or monotherapy, decreases FS-14, FAI, SCL-90, SAS, SDS, and clinical symptom scores and enhances IGA, IGG, IGM, and effective rate. However, NK cell levels did not change significantly. In addition, the included studies did not report serious adverse events, suggesting that CHM is relatively safe in patients with CFS. Our findings on commonly used CHM may help investigate their value and further clinical application for CFS. Our study suggests that CHM seems to be effective and safe in the treatment of CFS. However, given the poor quality of the included studies, more international multi-centered, double-blinded, placebo-controlled, well-designed clinical trials are needed in future research.
