# Supplementary material for: Chinese herbal medicine for the treatment of chronic fatigue syndrome: A systematic review and meta-analysis
Source: Front Pharmacol. 2022 Sep 29;13:958005. doi: 10.3389/fphar.2022.958005 (PMC9557005; doi:10.3389/fphar.2022.958005)
Supplement: Supplementary file 1 [file DataSheet2.pdf]

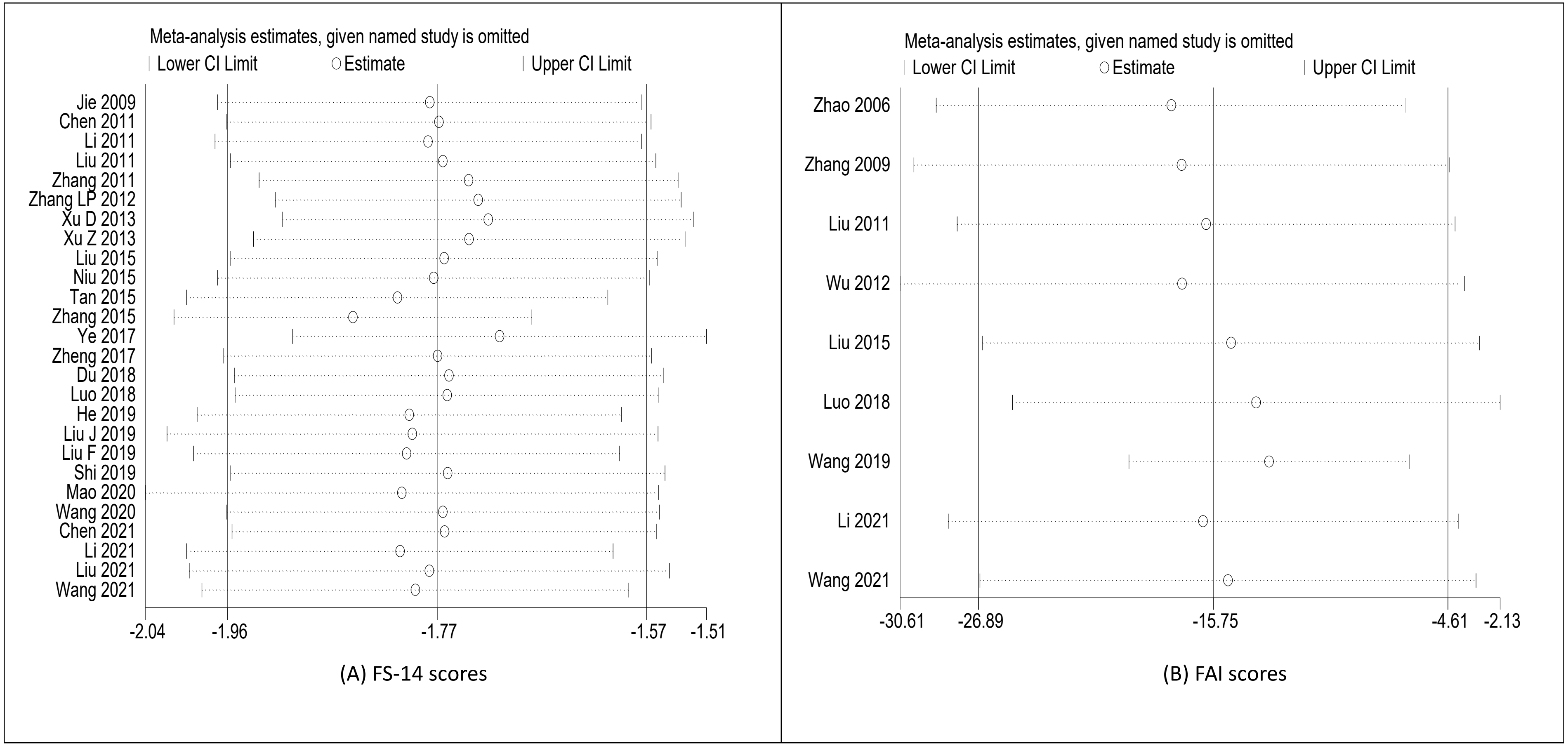
Meta-analysis estimates, given named study is omitted
Lower CI Limit
Estimate
Upper CI Limit
Jie 2009
Chen 2011
Li 2011
Liu 2011
Zhang 2011
Zhang LP 2012
Xu D 2013
Xu Z 2013
Liu 2015
Niu 2015
Tan 2015
Zhang 2015
Ye 2017
Zheng 2017
Du 2018
Luo 2018
He 2019
Liu J 2019
Liu F 2019
Shi 2019
Mao 2020
Wang 2020
Chen 2021
Li 2021
Liu 2021
Wang 2021
-2.04
-1.96
-1.77
-1.57
-1.51
(A) FS-14 scores
Meta-analysis estimates, given named study is omitted
Lower CI Limit
Estimate
Upper CI Limit
Zhao 2006
Zhang 2009
Liu 2011
Wu 2012
Liu 2015
Luo 2018
Wang 2019
Li 2021
Wang 2021
-30.61
-26.89
-15.75
-4.61
-2.13
(B) FAI scores

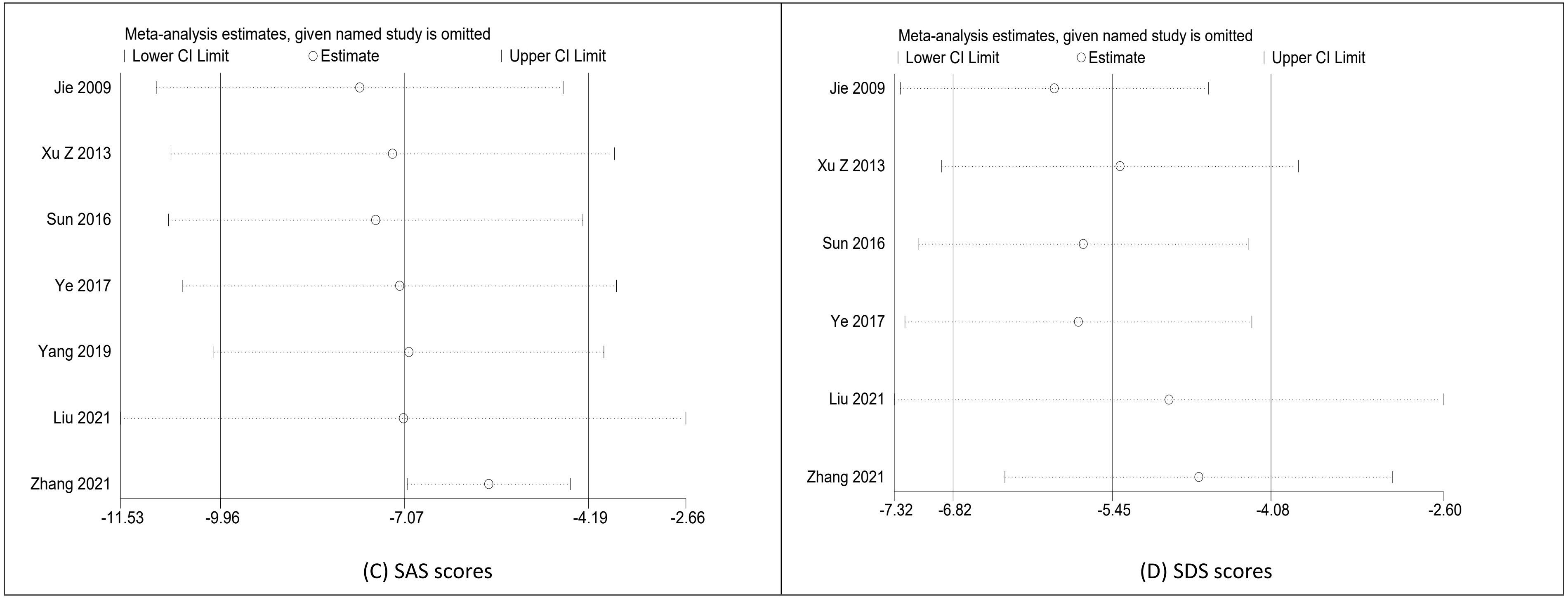

Meta-analysis estimates, given named study is omitted
Lower CI Limit
Estimate
Upper CI Limit
Jie 2009
Xu Z 2013
Sun 2016
Ye 2017
Yang 2019
Liu 2021
Zhang 2021
-11.53
-9.96
-7.07
-4.19
-2.66
(C) SAS scores
Meta-analysis estimates, given named study is omitted
Lower CI Limit
Estimate
Upper CI Limit
Jie 2009
Xu Z 2013
Sun 2016
Ye 2017
Liu 2021
Zhang 2021
-7.32
-6.82
-5.45
-4.08
-2.60
(D) SDS scores

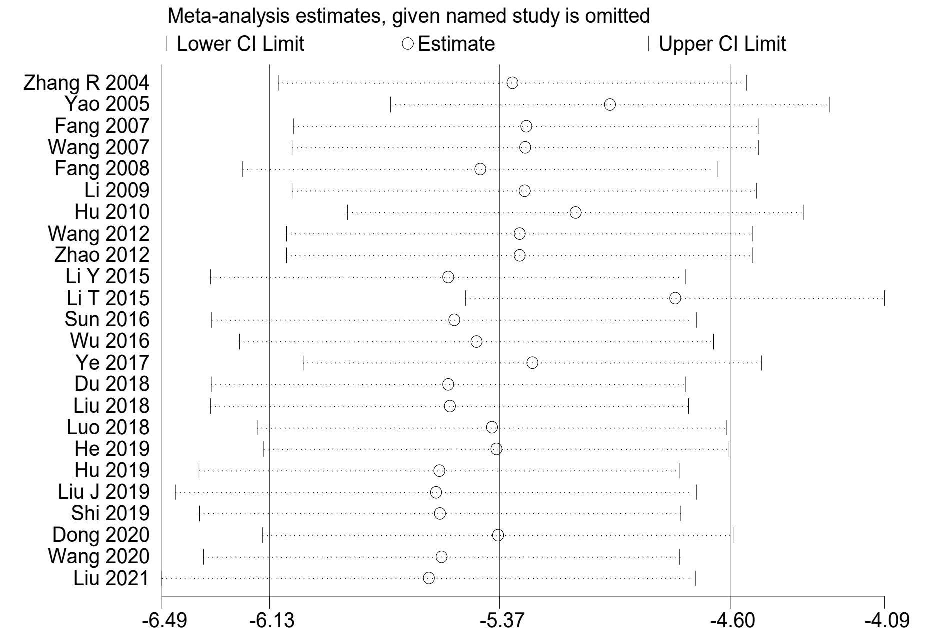

(E) Clinical symptom scores

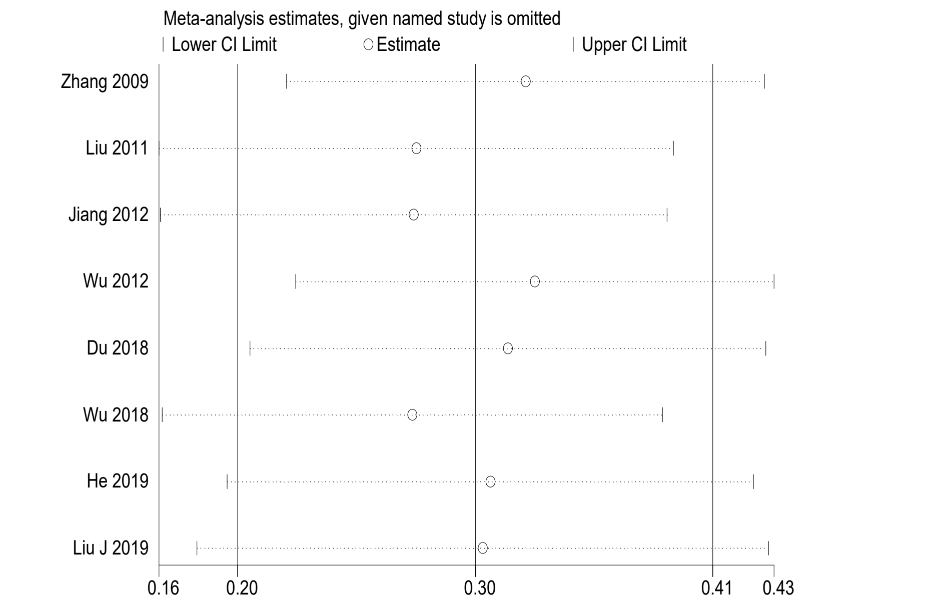

(F) IGA

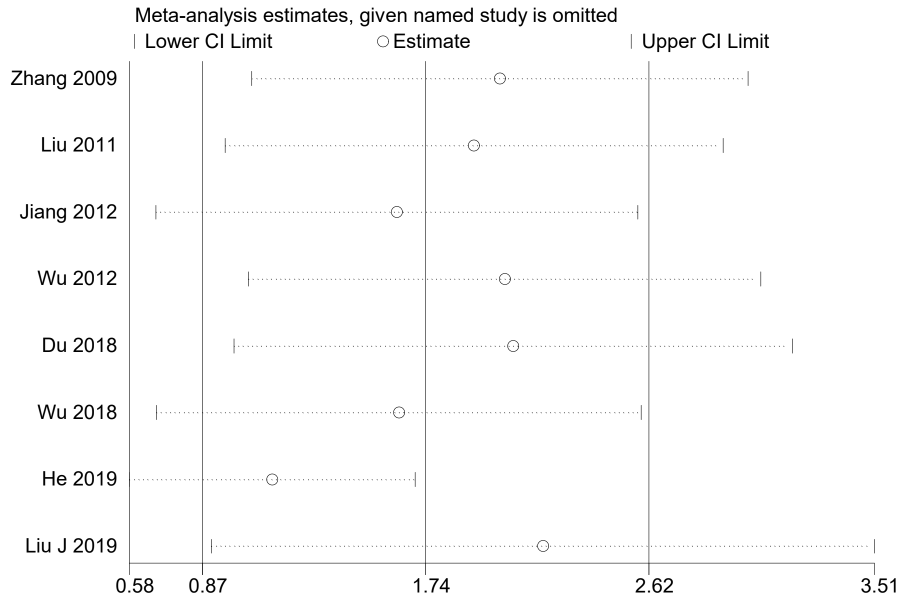
Meta-analysis estimates, given named study is omitted
Lower CI Limit
Estimate
Upper CI Limit
Zhang 2009
Liu 2011
Jiang 2012
Wu 2012
Du 2018
Wu 2018
He 2019
Liu J 2019
0.58
0.87
1.74
2.62
3.51

(G) IGG

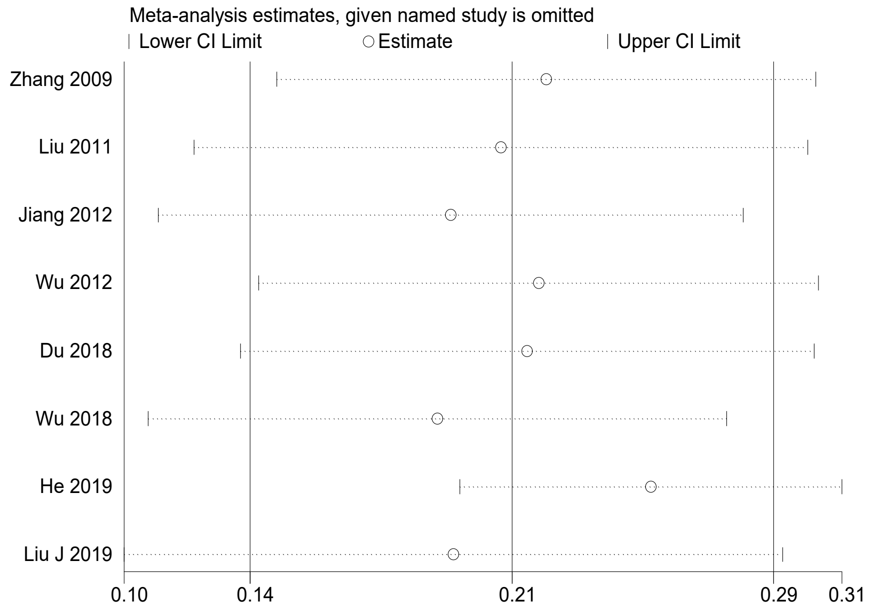
Meta-analysis estimates, given named study is omitted
Lower CI Limit
Estimate
Upper CI Limit
Zhang 2009
Liu 2011
Jiang 2012
Wu 2012
Du 2018
Wu 2018
He 2019
Liu J 2019
0.10
0.14
0.21
0.29
0.31

(H) IGM

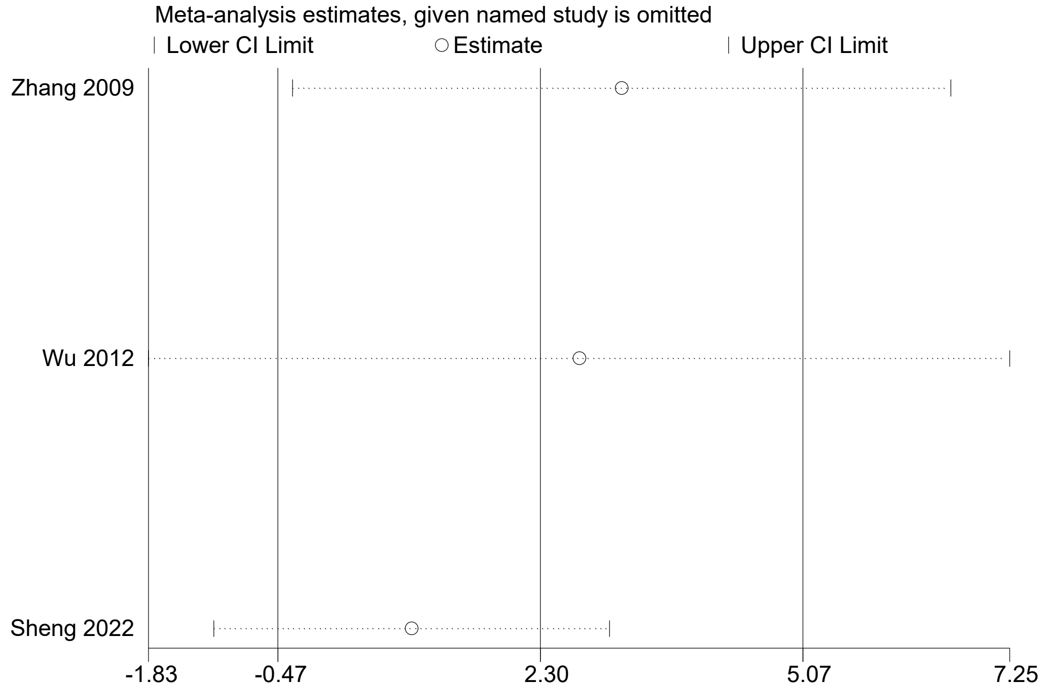

Meta-analysis estimates, given named study is omitted
Lower CI Limit
Estimate
Upper CI Limit
Zhang 2009
Wu 2012
Sheng 2022
-1.83
-0.47
2.30
5.07
7.25

(I) NK cell

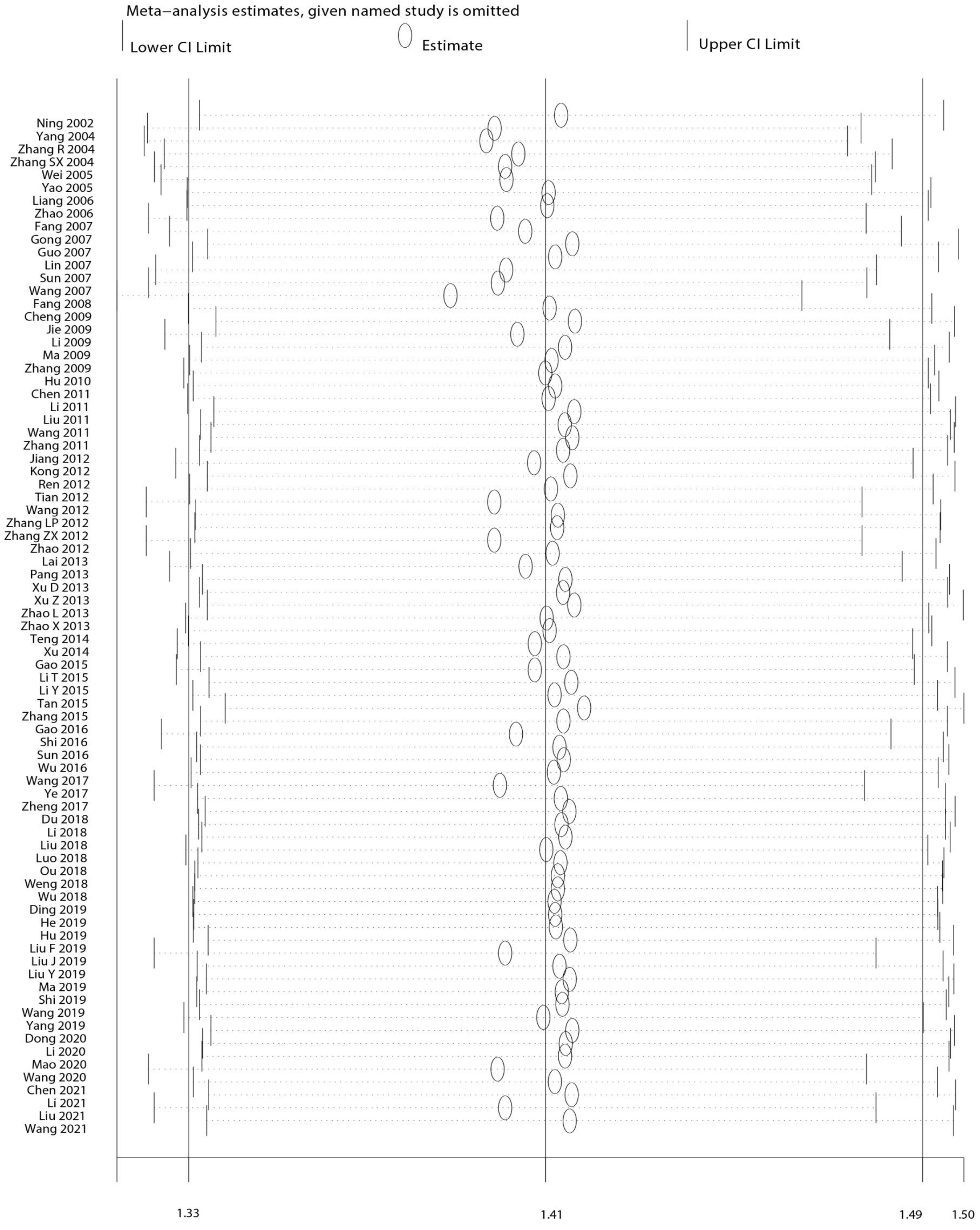

(J) Effective rate

Additional file 2 Sensitivity analysis.

(A) FS-14 scores, (B) FAI scores, (C) SAS scores, (D) SDS scores, (E) Clinical symptom scores, (F) IGA, (G) IGG, (H) IGM, (I) NK Cell, (J)Effective rate.
