# Supplementary figures and images for: Chinese herbal medicine for the treatment of chronic fatigue syndrome: A systematic review and meta-analysis
Source: Front Pharmacol. 2022 Sep 29;13:958005. doi: 10.3389/fphar.2022.958005 (PMC9557005; doi:10.3389/fphar.2022.958005)

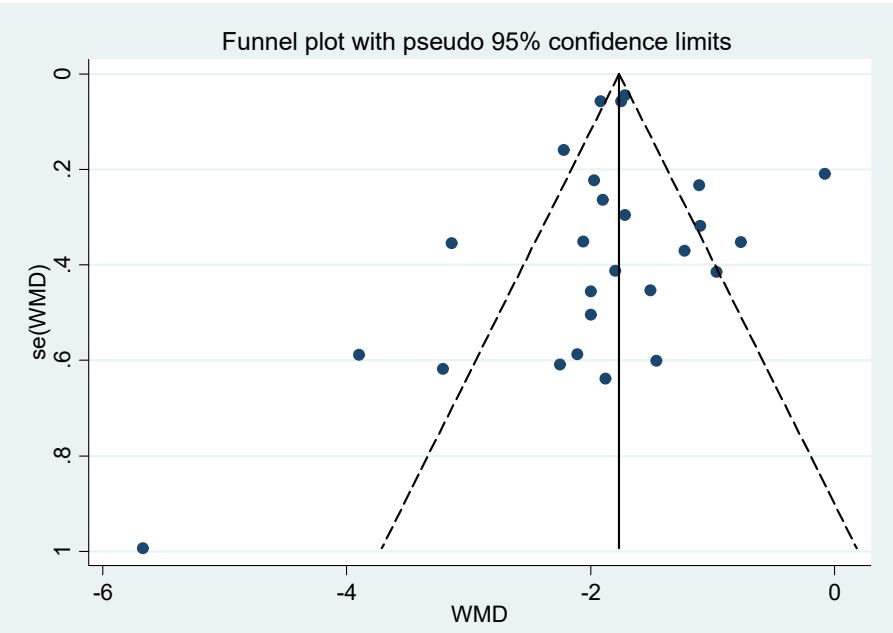

(A) Funnel plot

Egger's test

| Std_Eff | Coef.     | Std. Err. | t      | P> t  | [95% Conf. Interval] |           |
|---------|-----------|-----------|--------|-------|----------------------|-----------|
| slope   | -1.748983 | .0937743  | -18.65 | 0.000 | -1.942523            | -1.555442 |
| bias    | -.1755746 | .6673077  | -0.26  | 0.795 | -1.55283             | 1.201681  |

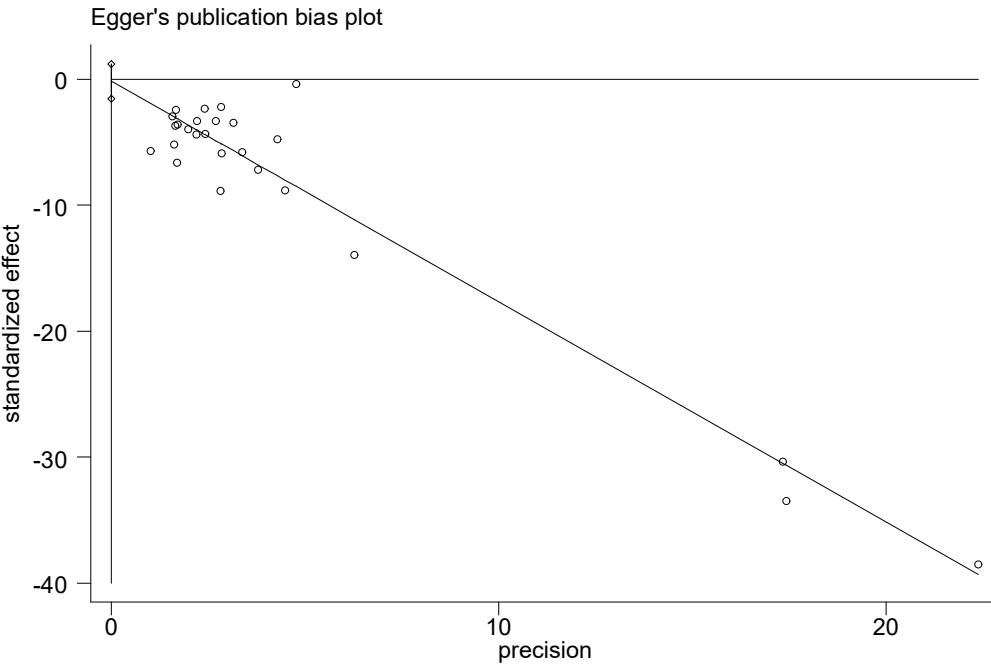

(B) Egger's test

Supplement: Supplementary file 2 [file DataSheet3.pdf]
