# Supplementary material for: Chinese herbal medicine for the treatment of chronic fatigue syndrome: A systematic review and meta-analysis
Source: Front Pharmacol. 2022 Sep 29;13:958005. doi: 10.3389/fphar.2022.958005 (PMC9557005; doi:10.3389/fphar.2022.958005)
Supplement: Supplementary file 3 [file DataSheet1.pdf]

**Online Appendix 1 Details of Search Strategy Source: PubMed; Searched on: May 2022; Results: 123**

| Search | Query |
|---|---|
| #1 | "Fatigue Syndrome, Chronic"[Mesh] |
| #2 | Fatigue Syndrome, Chronic[Title/Abstract] |
| #3 | chronic fatigue syndrome[Title/Abstract] |
| #4 | CFS[Title/Abstract] |
| #5 | Chronic Fatigue Disorder[Title/Abstract] |
| #6 | Fatigue Disorder, Chronic[Title/Abstract] |
| #7 | Myalgic Encephalomyelitis[Title/Abstract] |
| #8 | ME [Title/Abstract] |
| #9 | Encephalomyelitis, Myalgic[Title/Abstract] |
| #10 | Systemic Exertion Intolerance Disease[Title/Abstract] |
| #11 | Chronic Fatigue and Immune Dysfunction Syndrome[Title/Abstract] |
| #12 | #1 OR #2 OR #3 OR #4 OR #5 OR #6 OR #7 OR #8 OR #9 OR #10 OR #11 |
| #13 | chinese herbal medicine[Title/Abstract] |
| #14 | Chinese traditional[Title/Abstract] |
| #15 | oriental traditional[Title/Abstract] |
| #16 | traditional Chinese medicine[Title/Abstract] |
| #17 | traditional Chinese medicinal materials[Title/Abstract] |
| #18 | chinese herb* [Title/Abstract] |
| #19 | herbal medicine[Title/Abstract] |
| #20 | herbal or decoction[Title/Abstract] |
| #21 | Tang[Title/Abstract] |
| #22 | pill* [Title/Abstract] |
| #23 | wan[Title/Abstract] |
| #24 | Powder[Title/Abstract] |
| #25 | Formula[Title/Abstract] |
| #26 | Granule*[Title/Abstract] |
| #27 | Capsule[Title/Abstract] |
| #28 | Particles[Title/Abstract] |
| #29 | Ointment[Title/Abstract] |
| #30 | Prescription[Title/Abstract] |
| #31 | Receipt[Title/Abstract] |
| #32 | #13 OR #14 OR #15 OR #16 OR #17 OR #18 OR #19 OR #20 OR #21 OR #22 OR #23 OR #24 OR #25 OR #26 OR #27 OR #28 OR#29 OR#30 OR#31 |
| #33 | random* controlled trial[Title/Abstract] |
| #34 | RCT[Title/Abstract] |
| #35 | random* [Title/Abstract] |
| #36 | placebo[Title/Abstract] |
| #37 | #33 OR #34 OR #35 OR #36 |
| #38 | #12 AND #32 AND #37 |

**Source: Cochrane Library; Searched on: May 2022; Results: 457**

| Search | Query |
|---|---|
| #1 | MeSH descriptor: [Fatigue Syndrome, Chronic] explode all trees |
| #2 | (Fatigue Syndrome, Chronic):ti,ab,kw |
| #3 | (chronic fatigue syndrome):ti,ab,kw |
| #4 | (CFS):ti,ab,kw |
| #5 | (Chronic Fatigue Disorder):ti,ab,kw |
| #6 | (Fatigue Disorder, Chronic):ti,ab,kw |
| #7 | (Myalgic Encephalomyelitis):ti,ab,kw |
| #8 | (ME):ti,ab,kw |
| #9 | (Encephalomyelitis, Myalgic):ti,ab,kw |
| #10 | (Systemic Exertion Intolerance Disease):ti,ab,kw |
| #11 | (Chronic Fatigue and Immune Dysfunction Syndrome):ti,ab,kw |
| #12 | #1 OR #2 OR #3 OR #4 OR #5 OR #6 OR #7 OR #8 OR #9 OR #10 OR #11 |
| #13 | (Chinese herbal medicine):ti,ab,kw |
| #14 | (Chinese traditional):ti,ab,kw |
| #15 | (Oriental traditional):ti,ab,kw |
| #16 | (Traditional Chinese medicine):ti,ab,kw |
| #17 | (Traditional Chinese medicinal materials):ti,ab,kw |
| #18 | (Chinese herb*):ti,ab,kw |
| #19 | (Herbal medicine):ti,ab,kw |
| #20 | (Herbal or decoction):ti,ab,kw |
| #21 | (Tang):ti,ab,kw |
| #22 | (Pill*):ti,ab,kw |
| #23 | (Wan):ti,ab,kw |
| #24 | (Powder):ti,ab,kw |
| #25 | (Formula):ti,ab,kw |
| #26 | (Granule*):ti,ab,kw |
| #27 | (Capsule):ti,ab,kw |
| #28 | (Particles):ti,ab,kw |
| #29 | (Ointment):ti,ab,kw |
| #30 | (Prescription):ti,ab,kw |
| #31 | (Receipt):ti,ab,kw |
| #32 | #13 OR #14 OR #15 OR #16 OR #17 OR #18 OR #19 OR #20 OR #21 OR #22 OR #23 OR #24 OR #25 OR #26 OR #27 OR #28 OR#29 OR#30 OR#31 |
| #33 | (Random* controlled tria):ti,ab,kw |
| #34 | (RCT):ti,ab,kw |
| #35 | (Random* ):ti,ab,kw |
| #36 | (Placebo):ti,ab,kw |
| #37 | #33 OR #34 OR #35 OR #36 |
| #38 | #12 AND #32 AND #37 |

**Source: Embase; Searched on: May 2022; Results: 194**

| Search | Query |
|---|---|
| #1 | 'Fatigue Syndrome, Chronic'/exp |

| | |
|---|---|
| #2 | 'Fatigue Syndrome, Chronic':ab,ti |
| #3 | 'chronic fatigue syndrome':ab,ti |
| #4 | 'CFS':ab,ti |
| #5 | 'Chronic Fatigue Disorder':ab,ti |
| #6 | 'Fatigue Disorder, Chronic':ab,ti |
| #7 | 'Myalgic Encephalomyelitis':ab,ti |
| #8 | 'ME':ab,ti |
| #9 | 'Encephalomyelitis, Myalgic':ab,ti |
| #10 | 'Systemic Exertion Intolerance Disease':ab,ti |
| #11 | 'Chronic Fatigue and Immune Dysfunction Syndrome':ab,ti |
| #12 | #1 OR #2 OR #3 OR #4 OR #5 OR #6 OR #7 OR #8 OR #9 OR #10 OR #11 |
| #13 | 'Chinese herbal medicine' |
| #14 | 'Chinese traditional':ab,ti |
| #15 | 'Oriental traditiona'l:ab,ti |
| #16 | 'Traditional Chinese medicine':ab,ti |
| #17 | 'Traditional Chinese medicinal materials':ab,ti |
| #18 | 'Chinese herb*':ab,ti |
| #19 | 'Herbal medicine':ab,ti |
| #20 | 'Herbal or decoction':ab,ti |
| #21 | 'Tang':ab,ti |
| #22 | 'Pill*':ab,ti |
| #23 | 'Wan':ab,ti |
| #24 | 'Powder':ab,ti |
| #25 | 'Formula':ab,ti |
| #26 | 'Granule*:ab,ti |
| #27 | 'Capsule':ab,ti |
| #28 | 'Particles':ab,ti |
| #29 | 'Ointment':ab,ti |
| #30 | 'Prescription':ab,ti |
| #31 | 'Receipt':ab,ti |
| #32 | #13 OR #14 OR #15 OR #16 OR #17 OR #18 OR #19 OR #20 OR #21 OR #22 OR #23 OR #24 OR #25 OR #26 OR #27 OR #28 OR#29 OR#30 OR#31 |
| #33 | 'random* controlled tria':ab,ti |
| #34 | 'RCT':ab,ti |
| #35 | 'Random*':ab,ti |
| #36 | 'Placebo':ab,ti |
| #37 | #33 OR #34 OR #35 OR #36 |
| #38 | #12 AND #32 AND #37 |

**Source: Web of Science; Searched on: May 2022; Results: 351**

| Search | Query |
|---|---|
| #1 | TS="Fatigue Syndrome, Chronic" |
| #2 | TS="Fatigue Syndrome, Chronic" |
| #3 | TS="chronic fatigue syndrome” |

| | |
|---|---|
| #4 | TS="CFS " |
| #5 | TS="Chronic Fatigue Disorder" |
| #6 | TS="Fatigue Disorder, Chronic" |
| #7 | TS="Myalgic Encephalomyelitis" |
| #8 | TS="ME" |
| #9 | TS="Encephalomyelitis, Myalgic" |
| #10 | TS="Systemic Exertion Intolerance Disease" |
| #11 | TS="Chronic Fatigue and Immune Dysfunction Syndrome" |
| #12 | #1 OR #2 OR #3 OR #4 OR #5 OR #6 OR #7 OR #8 OR #9 OR #10 OR #11 |
| #13 | TS="Chinese herbal medicine" |
| #14 | TS="Chinese traditional" |
| #15 | TS="Oriental traditional" |
| #16 | TS="Traditional Chinese medicine" |
| #17 | TS="Traditional Chinese medicinal materials" |
| #18 | TS="Herbal medicine" |
| #19 | TS="Chinese herb* " |
| #20 | TS="Herbal or decoction" |
| #21 | TS="Tang" |
| #22 | TS="pill*" |
| #23 | TS="wan" |
| #24 | TS="Powder" |
| #25 | TS="Formula" |
| #26 | TS="Granule*" |
| #27 | TS="Capsule" |
| #28 | TS="Particles" |
| #29 | TS="Ointment" |
| #30 | TS="Prescription" |
| #31 | TS="Receipt" |
| #32 | #13 OR #14 OR #15 OR #16 OR #17 OR #18 OR #19 OR #20 OR #21 OR #22 OR #23 OR #24 OR #25 OR #26 OR #27 OR #28 OR#29 OR#30 OR#31 |
| #33 | TS="Random* controlled trial" |
| #34 | TS="RCT" |
| #35 | TS="Random*" |
| #36 | TS="Placebo" |
| #37 | #33OR #34 OR #35 OR #36 |
| #38 | #12 AND #32 AND #37 |

**Search in Chinese: CNKI; Results: 250**

TKA=(manxingpilaozonghezheng+manxingpilaozonghezheng+jitongxingnaojisuiyan+manxingpilaomianyigongnengzhangaizonghezheng+pilaozonghezheng) AND TKA=(tang+san+wan+keli+fang+zhongyao+zhongcaoyao+pian+fa+gao+jiaonang+yin+ye+ji) AND FT=(suiji) NOT TKA=(zhenci + zhen + zhenjiu + jiu + jjnzhan + jizhi + shu + aiyinxingpifa + baguan + xuewei + qi + jinyan + shuhoupifa + tuina)

**Search in Chinese: Wanfang Database; Results: 267**

Zhuti:(manxingpilaozonghezheng+manxingpilaozonghezheng+jitongxingnaojisuiyan+manxingpilaomianyigongnengzhangaizonghezheng+pilaozonghezheng) * Zhuti:(tang+san+wan+keli+fang+zhongyao+zhongcaoyao+pian+fa+gao+jiaonang+yin+ye+ji)*Quanbu:(suiji) NOT Zhuti: (zhenci + zhen + zhenjiu + jiu + jjnzhan + jizhi + shu + aiyinxingpifa + baguan + xuewei + qi + jinyan + shuhoupifa + tuina)

**Search in Chinese: VIP database; Results: 158**

(M=manxingpilaozonghezheng+manxingpilaozonghezheng+jitongxingnaojisuiyan+manxingpilaomianyigongnengzhangaizonghezheng+pilaozonghezheng)*(M=tang+san+wan+keli+fang+zhongyao+zhongcaoyao+pian+fa+gao+jiaonang+yin+ye+ji)*(M=suiji) NOT (M=zhenci + zhen + zhenjiu + jiu + jjnzhan + jizhi + shu + aiyinxingpifa + baguan + xuewei + qi + jinyan + shuhoupifa + tuina)

**The US Clinical Trials Registry: 3**

Condition or disease: Fatigue Syndrome, Chronic OR Fatigue Syndrome, Chronic OR CFS OR Chronic Fatigue Syndrome OR Myalgic Encephalomyelitis OR ME OR Encephalomyelitis, Myalgic OR Chronic Fatigue Disorder OR Fatigue Disorder, Chronic OR Systemic Exertion Intolerance Disease

Intervention/treatment:Chinese herbal medicine OR Chinese traditional OR Oriental traditional OR traditional Chinese medicine OR traditional Chinese medicinal materials OR chinese herb OR herbal medicine OR herbal OR decoction OR tang OR pill OR wan OR powder OR Formula

**The Chinese Clinical Trials Registry : 26**

Subject of registration: manxingpilaozonghezheng
